# Why is manganese so valuable to bacterial pathogens?

**DOI:** 10.3389/fcimb.2023.943390

**Published:** 2023-02-03

**Authors:** Jan Čapek, Branislav Večerek

**Affiliations:** Laboratory of post-transcriptional control of gene expression, Institute of Microbiology of the Czech Academy of Sciences, Prague, Czechia

**Keywords:** manganese, metallostasis, pathogenesis, mismetallation, iron, oxidative stress

## Abstract

Apart from oxygenic photosynthesis, the extent of manganese utilization in bacteria varies from species to species and also appears to depend on external conditions. This observation is in striking contrast to iron, which is similar to manganese but essential for the vast majority of bacteria. To adequately explain the role of manganese in pathogens, we first present in this review that the accumulation of molecular oxygen in the Earth’s atmosphere was a key event that linked manganese utilization to iron utilization and put pressure on the use of manganese in general. We devote a large part of our contribution to explanation of how molecular oxygen interferes with iron so that it enhances oxidative stress in cells, and how bacteria have learned to control the concentration of free iron in the cytosol. The functioning of iron in the presence of molecular oxygen serves as a springboard for a fundamental understanding of why manganese is so valued by bacterial pathogens. The bulk of this review addresses how manganese can replace iron in enzymes. Redox-active enzymes must cope with the higher redox potential of manganese compared to iron. Therefore, specific manganese-dependent isoenzymes have evolved that either lower the redox potential of the bound metal or use a stronger oxidant. In contrast, redox-inactive enzymes can exchange the metal directly within the individual active site, so no isoenzymes are required. It appears that in the physiological context, only redox-inactive mononuclear or dinuclear enzymes are capable of replacing iron with manganese within the same active site. In both cases, cytosolic conditions play an important role in the selection of the metal used. In conclusion, we summarize both well-characterized and less-studied mechanisms of the tug-of-war for manganese between host and pathogen.

## 1 Introduction

In recent decades, ample evidence has accumulated that manganese (Mn) is justifiably the subject of a tug-of-war between the human host and pathogens. If the pathogen’s access to manganese is not restricted during infection by the human host, the pathogen gains the upper hand in the struggle for survival. Surprisingly, only a few enzymes are known to be strictly manganese-dependent; others, possibly more numerous, use manganese only conditionally. Why are manganese-dependent enzymes relatively rare, and what causes some enzymes to use manganese only conditionally?

**Figure 1:**
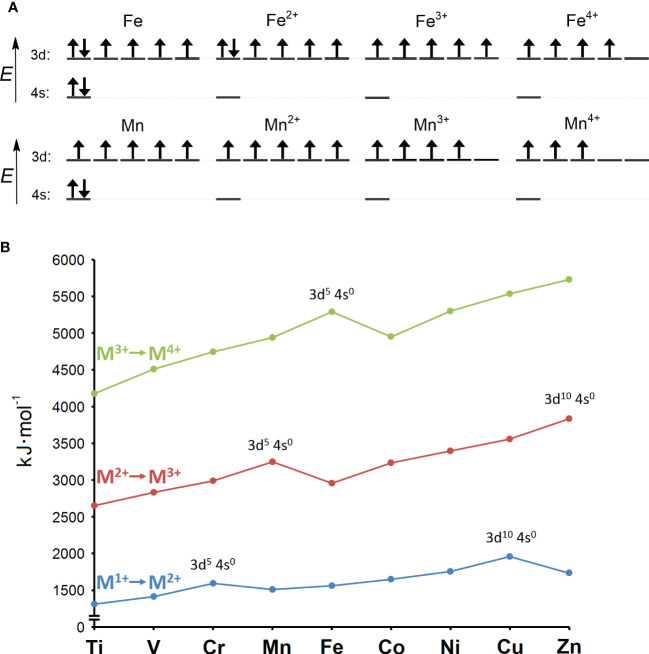
**(A)** Electron configurations of iron and manganese atoms and their +2, +3, and +4 oxidation states. The configurations of the +2 and +3 oxidation states for iron and manganese correspond to the starting ions in the ionization scheme shown in panel **(B). (B)** Second (M^1+^ to M^2+^; in blue), third (M^2+^ to M^3+^; in red) and fourth (M^3+^ to M^4+^; in green) ionization energies of the first-row transition metals. The ionization energy is the work required to remove an electron from an atom or ion, and it increases toward higher atomic numbers within a single valence shell. This rule is violated by half-filled (3d^5^4s^0^) and fully filled d-orbitals (3d^10^4s^0^), which have a higher ionization energy than the following atom or ion with a higher atomic number. This is because symmetric half-filled or fully filled orbitals are stabilized by released exchange and pairing energies, which ultimately impedes their further oxidation. Mn^2+^ is stabilized and therefore difficult to oxidize because it has symmetric 3d^5^4s^0^ configuration, while Fe^2+^ has asymmetric 3d^6^4s^0^ configuration and is therefore easier to oxidize. On the other hand, the half-filled 3d^5^4s^0^ configuration of Fe^3+^ renders this ion more resistant to further oxidation compared to the asymmetric 3d^4^4s^0^ configuration of Mn^3+^. Adapted from Lang and Smith (2003).

**Figure 2:**
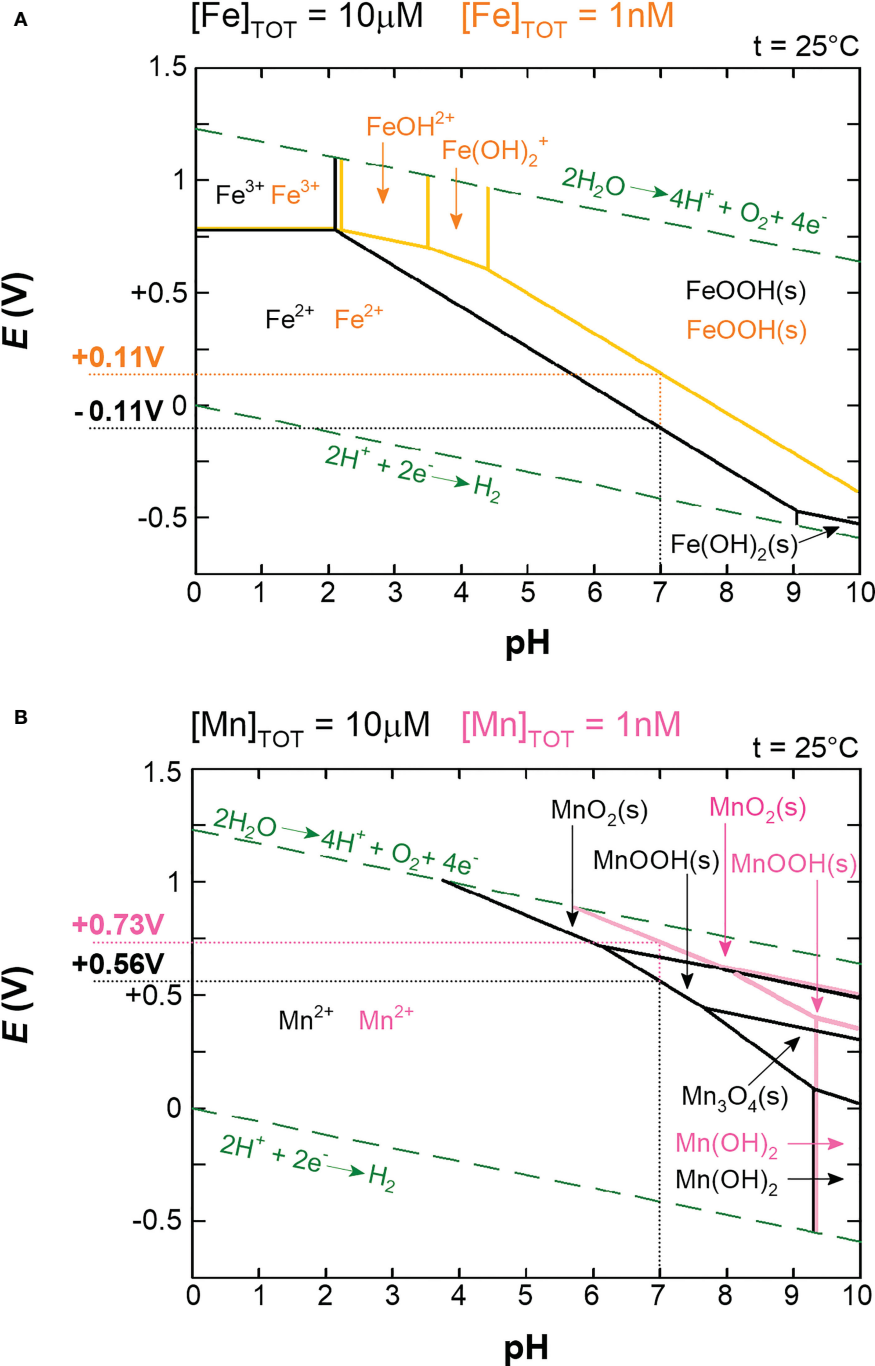
Pourbaix diagrams for iron **(A)** and manganese **(B)**. In both diagrams, precipitates (s) are probably suppressed by complexation in living cells. The green dashed lines indicate the stability of the water. The solid lines (black, orange, and pink) represent redox and acid-base reactions, and on these lines the two species are in equilibrium. The areas delineated from the equilibria indicate which oxidation state is predominant. Two concentrations are shown for each metal: 10μM (black lines) and 1nM (orange lines for iron and pink lines for manganese). 10μM concentration roughly corresponds to intracellular free pools in aerobically grown cells and 1nM roughly corresponds to oxygen-rich environmental conditions (Anbar, 2008; Beauchene et al., 2017). Actual redox potentials (*E*) for iron and manganese at corresponding concentrations and at pH 7 were subtracted from the graphs and are shown next to the *y*-axis. Note that the standard redox potentials (*E*°’) are measured at standard conditions (1M concentrations of reactants and pH 7) and are related to the actual redox potentials (*E*) *via* the Nernst equation (Eq. 1). For simplicity, we use the term redox potential to refer to the actual redox potential (*E*) unless explicitly stated otherwise. $\text{Eq. }1:\;E=E^{0’}-\frac{RT}{zF}\text{ln}\frac{\left[\text{reduced substrate}\right]}{\left[\text{oxidised substrate}\right]}$ *R* = 8.314 J·K^−1^·mol^−1^, *F* = 96 485.332 C·mol^−1^, *T* is the temperature in kelvins, *z* is the number of electrons transferred, and in parentheses are the concentrations (more precisely, the activities) of the oxidized and reduced forms of the substrate. Pourbaix diagrams were constructed using the software MEDUSA and the associated database HYDRA (https://www.kth.se/che/medusa/downloads accessed on 1. June 2022).

The answers to these questions can be better understood by considering the major change in ocean composition caused by Earth’s oxygenation. The presence of molecular oxygen (O_2_) is a consequence of the invention of oxygenic photosynthesis by cyanobacteria more than 2.5 billion years ago (Sánchez-Baracaldo and Cardona, 2020). Before that, the Earth was essentially devoid of O_2_. Under anoxic conditions, the concentration of the soluble (bioavailable) form of iron (Fe^2+^) is estimated to have been 10^-5^ M, in contrast to bioavailable manganese (Mn^2+^), with an estimated concentration of less than 10^-8^ M. In modern oxygen-rich oceans, the concentration of Fe^2+^ is 10^-9^ M and that of Mn^2+^ is slightly more than 10^-10^ M (Anbar, 2008). In summary, when prokaryotic life emerged the concentration difference between Fe^2+^ and Mn^2+^ was more than 1,000 times, whereas today it is less than 10 times. This observation reflects the crucial difference between Fe^2+^ and Mn^2+^, namely their readiness to be oxidized. Qualitatively, the symmetric 3d^5^ electronic configuration of Mn^2+^, in contrast to the asymmetric 3d^6^ configuration of Fe^2+^, reveals why Mn^2+^ is not as easily oxidized as Fe^2+^ (**Figure 1**). In quantitative terms, the redox potential (*E*) is used in a redox reaction to indicate whether a particular chemical substance tends to lose an electron, *i.e.*, to be oxidized (lower *E*) or gain an electron, *i.e.*, to be reduced (higher *E*). Pourbaix diagrams are used to show the dependence of redox potential on concentration and pH (**Figure 2**). **Figure 2** shows that Mn^2+^ is much more resistant to oxidation compared to Fe^2+^, implying that Mn^2+^ was probably not redox active in anoxic oceans, while redox cycling between Fe^2+^ and Fe^3+^ was available for the first life forms. Surprisingly, apart from processes involving electron transfers and slight differences in polarizability (Jensen, 1978), Fe^2+^ and Mn^2+^ are apparently perceived by biological systems to be very similar and difficult to distinguish, as competitive interactions for the same binding sites are frequently reported (Hantke, 1987; Beyer and Fridovich, 1991; Boyer et al., 2002; Fitsanakis et al., 2010; Cotruvo and Stubbe, 2012; Dudev and Lim, 2014). Nevertheless, according to the Irving-Williams series (Irving and Williams, 1948), Mn^2+^ is less competitive than Fe^2+^, and if such competition occurred in primordial anoxic oceans, one could speculate that Mn^2+^ had a low chance of being taken up by microbes instead of Fe^2+^. Therefore, Mn^2+^ could not be utilized in redox reactions prior to O_2_ accumulation, and since it is otherwise similar to Fe^2+^ but occurs in much lower concentrations, the likelihood of manganese being used in the biosphere was probably rather low.

One of the first biological reactions based entirely on manganese was likely water splitting in the photosystem II, in which manganese cluster is oxidized by trapped photons (Barber, 2017). The oxidized manganese cluster in turn oxidizes water to release O_2_, which in turn dramatically expanded the range of redox chemistry and made the redox cycling between Mn^2+^ and Mn^3+^ accessible to non-photosynthetic organisms as well (Lingappa et al., 2019). The low redox potential of iron, appreciated by microbes in anoxic oceans, became a burden in aerated areas as Fe^2+^ oxidizes to Fe^3+^, which precipitates, leading to a decrease in bioavailable iron. In addition, random oxidation of Fe^2+^ contributes significantly to oxidative stress (see Section 2). In contrast, Mn^2+^ is more stable under oxygen-rich conditions compared to Fe^2+^, so its concentrations did not decrease as dramatically. This opened the possibility for Mn^2+^ to effectively compete with Fe^2+^ in some redox-inactive enzymes (discussed in detail in Section 4.2). Such substitution is beneficial as it confers resistance to oxidative stress to the enzymes. In addition, the newly accessible redox cycling of manganese has been exploited by some enzymes, which have thus formed alternates to their iron-dependent counterparts (see Section 4.1). Prokaryotes had to learn to cope with O_2_ and its reactivity toward Fe^2+^, and during this process the increased use of manganese instead of iron could lead to better robustness in aerated habitats. However, it appears that the oxygenation of Earth occurred too late in the evolutionary history of metabolism that was already tailored to iron, and one might speculate that this could be the reason why purely manganese-dependent enzymes lacking an iron-dependent alternate are very rare. In aerobes or facultative anaerobes that use iron as a cofactor, nature has succeeded in maintaining their metabolism largely undisturbed as long as they are not exposed to oxidative stress. This is the case with bacterial pathogens, which have to cope with an oxidative burst triggered by the host’s innate immune system.

## 2 Iron, a Trojan horse of oxidative stress

**Figure 3:**
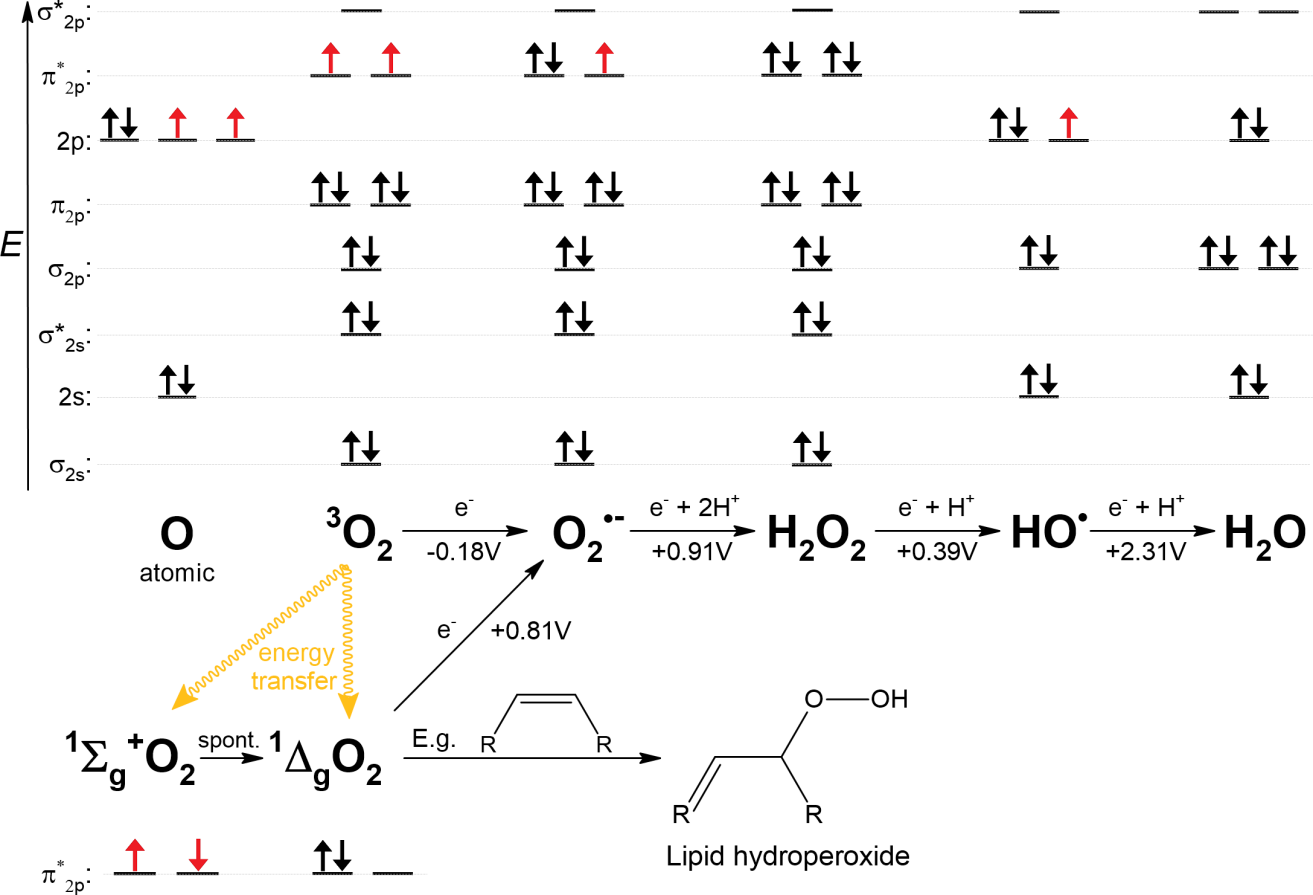
Various oxygen-containing species formed either by energy transfer or by reduction of a single electron from O_2_. ^1^Σ_g_^+^O_2_ is short-lived and rapidly converts to ^1^Δ_g_O_2_, the biologically relevant form. Standard redox potentials at pH 7 (*E*°’) are shown below the arrows, as summarized by Koppenol et al. (2010). Fe^2+^ is a plausible candidate as a single-electron donor at all indicated reductions. The electron configurations of the valence shells are shown above the corresponding species; in the case of singlet oxygen, only anti-bonding π* orbitals are shown. Unpaired electrons are shown in red. Hybridization of orbitals was not considered for this plot. For example, in the case of a water molecule, non-bonding electron pairs in 2s and 2p orbitals would form energetically equal lone electron pairs.

The electronic configuration of O_2_ shows that in its ground state (triplet state denoted ^3^O_2_), it is a di-radical, which means that two individual electrons are in separate orbitals with the same spins (**Figure 3**). Most stable molecules have paired electrons with oppositely oriented spins in their orbitals. This precludes ^3^O_2_ from directly accepting two electrons, since one of the electrons would have to change its spin orientation to fit the electronic configuration of ^3^O_2_ (Fridovich, 2013). This barrier is called a spin restriction and its great importance is best illustrated by a comparison with singlet O_2_ (designated ^1^O_2_), in which, in its more stable form, both electrons are paired in one degenerated π* orbital (**Figure 3**). Since ^1^O_2_ is not limited by spin restriction it is very reactive and, for example, rapidly forms addition products (hydroperoxides) with double bonds in unsaturated lipids (Girotti, 1990; Edge and Truscott, 2018). From now on, O_2_ refers to its triplet state. Unless catalyzed by special enzymes such as terminal oxidase (Babcock and Wikstrom, 1992), O_2_ can undergo only a single-electron reduction. However, the first single-electron reduction of O_2_ is an unfavorable step, as shown by the negative standard redox potential of O_2_ (**Figure 3**). Overall, O_2_ is generally a benign molecule when in contact with organic material due to spin limitation and resistance to accept the first electron. However, the greater the amount of free Fe^2+^ in the cytosol, the easier the oxidation of Fe^2+^ by O_2_ (**Figure 2**). Consistent with this, it has been observed in the facultative anaerobe *Escherichia coli* that upon shift from an anaerobic to an aerobic environment, the concentration of free cytosolic Fe^2+^ decreases, presumably to control its unwanted oxidation by O_2_ (Beauchene et al., 2017), or that increased oxidative damage is induced when iron is hyper-accumulated due to a genetic disorder of iron homeostasis (Touati et al., 1995).

The superoxide anion radical ( ${\text{O}}_{2}^{\mathrm{•-}}$ ) is the product of the single-electron reduction of O_2_. The standard redox potential of ${\text{O}}_{2}^{\mathrm{•-}}$ is much higher compared to O_2_ (**Figure 3**), but its reactivity is quite limited. ${\text{O}}_{2}^{\mathrm{•-}}$ can also accept only one electron, but it is negatively charged and therefore repelled by electron-dense targets; moreover, it requires protonation because the formation of a peroxide anion ( ${\text{O}}_{2}^{\mathrm{2-}}$ ) is unlikely, but protonation is hindered by the acidity of ${\text{O}}_{2}^{\mathrm{•-}}$ (p*K*a = 4.8). Although ${\text{O}}_{2}^{\mathrm{•-}}$ should be a strong oxidant from a simplified thermodynamic point of view, it is not an effective oxidant due to aforementioned limitations and therefore can only participate in a limited number of reactions *in vivo* (Koppenol and Butler, 1977; Koppenol et al., 2010; Winterbourn, 2020). Nevertheless, positively charged Fe^2+^ can bind ${\text{O}}_{2}^{\mathrm{•-}}$ , thereby stabilizing it by acting as a Lewis acid, whereupon the low redox potential of iron causes reduction of ${\text{O}}_{2}^{\mathrm{•-}}$ . Thus, intracellular Fe^2+^, both free and bound in enzymes, is a susceptible target for oxidation by ${\text{O}}_{2}^{\mathrm{•-}}$ (Benov, 2001; Gu and Imlay, 2013). Remarkably, the damaging effect of ${\text{O}}_{2}^{\mathrm{•-}}$ during the oxidative burst is enhanced by the presence of nitric oxide (NO^**·**^), which reacts rapidly with ${\text{O}}_{2}^{\mathrm{•-}}$ to form highly reactive peroxynitrite (ONOO^-^) (Beckman and Koppenol, 1996).

When ${\text{O}}_{2}^{\mathrm{•-}}$ is reduced and protonated, hydrogen peroxide (H_2_O_2_) is formed. H_2_O_2_ can be safely disposed of by reduction with two electrons, and when protonated at the same time, yields two water molecules, but when it reacts with Fe^2+^, only one electron is transferred to H_2_O_2_. By accepting one electron, H_2_O_2_ decomposes into two different molecules, the hydroxide anion (HO^−^) and the hydroxyl radical (HO^•^). HO^•^ is highly reactive without any limitation, the resulting cell damage is pleiotropic and ranges from lipid peroxidation to DNA aberrations (Halliwell and Gutteridge, 1992).

Although they behave differently in cells, ${\text{O}}_{2}^{\mathrm{•-}}$ , H_2_O_2_ and HO^•^ have been grouped under the term reactive oxygen species (ROS). On the other hand, all three species can be generated from O_2_ by reaction with Fe^2+^. Fe^2+^ fulfils the criteria for the reduction of O_2_ and ${\text{O}}_{2}^{\mathrm{•-}}$ and the strong oxidative effect of H_2_O_2_ in combination with Fe^2+^ was already described in the 1890s by Henry Fenton (Koppenol, 1993). The exact mechanism yielding HO^^•^^ as the product was formulated later by Haber and Weiss, but today this reaction is known as the Fenton reaction (Haber et al., 1934). At this point, it should be noted that the free Fe^2+^ pool is carefully controlled depending on the degree of oxidative stress (see Section 3) and under aerobic conditions represents only about 1% of the total iron content, as measured in *E. coli* (Keyer and Imlay, 1996). However, the majority of cellular iron is bound either oxidized in storage proteins or reduced in enzymes, and it has been reported that this latter (reduced) bound iron pool contributes significantly to the severity of oxidative stress (Imlay, 2013).

The underlying principle of oxidative stress lies in the unfortunate circumstance that Fe^2+^ is a cornerstone of enzymatic activity, while at the same time it is one of the few substances that can react with O_2_ and ${\text{O}}_{2}^{\mathrm{•-}}$ and serve as a single-electron reductant of H_2_O_2._ Other examples of single electron donors that can enhance oxidative stress include flavin prosthetic groups or quinones, which are used in low potential electron transfers but are not covered in this review (Imlay, 2013).

## 3 Exposure to oxidative stress impairs iron homeostasis

Pathogens evolved specific mechanisms to cope with both intrinsically and extrinsically induced oxidative burst in the host (Rada and Leto, 2008). Thus, the control of iron homeostasis is critical for pathogens. Iron is required to keep vital metabolic pathways going, but also serves as an accelerator of the formation of ROS. Therefore, mechanisms that reduce free cytosolic Fe^2+^ are as important as iron acquisition pathways. From the perspective of iron sequestration, prokaryotic ferritin (Ftn), bacterioferritin (Bfr), and DNA-binding protein from starved cells (Dps) are proteins that belong to the same ferritin superfamily and play an important role in storing excess iron (Honarmand Ebrahimi et al., 2015). Bfr and Ftn are induced when excess iron needs to be stored. However, Dps is usually subject to tighter regulation and is expressed, for example, during stationary phase or oxidative stress. This suggests that Dps represents one of the crucial adaptations to cope with the paradoxical behavior of iron under oxidative stress conditions (Altuvia et al., 1994; Bradley et al., 2020). Bfr and Ftn physiologically use O_2_ to oxidize Fe^2+^ in order to sequester it (Honarmand Ebrahimi et al., 2015). However, Dps is characterized by exceptional ferroxidase activity, which favors H_2_O_2_ as an oxidant of Fe^2+^ to form insoluble Fe^3+^-containing mineral, which in turn is safely deposited in the cavity of Dps dodecamer (Zhao et al., 2002). Moreover, in many species Dps also binds DNA and therefore, through its catalytic activity, removes Fe^2+^ and H_2_O_2_ from the site of the cell where the Fenton reaction could cause the most severe damage (Orban et al., 2022). The ferroxidase center in Dps consists of di-iron cluster and it is the close proximity of two ferrous ions that allows the reduction of H_2_O_2_ with two electrons yielding two water molecules and thereby suppresses the production of HO^•^ that would otherwise occur in the presence of a single Fe^2+^ ion (Zhao et al., 2002). Moreover, if HO^•^ is accidentally produced, the damage is directly taken by Dps. Indeed, it has been shown that the conserved tryptophan and tyrosine residues positioned near the ferroxidase center in Dps are converted to the radical forms after Fe^2+^ oxidation, thus trapping the dangerous radical before it can diffuse into the cytosol (Bellapadrona et al., 2010). A Dps ortholog, MrgA, has been identified in Gram-positive bacteria (Chen and Helmann, 1995). Overall, the activity of Dps suggests that bacteria need to reduce free cytosolic iron even further below the level achieved by standard means of iron storage performed by Bfr and/or Ftn (Mancini and Imlay, 2015; Sen et al., 2020).

Although the ability to sequester iron is important for bacterial pathogens, this activity should not interfere with the metallation of iron-dependent enzymes necessary for survival and proliferation. When concentrations of ROS are elevated during infection, pathogens most likely must compromise to maintain the pool of free cytosolic iron at a level that allows metallation of important iron-dependent enzymes and, on the other hand, potentiates oxidative stress to a tolerable extent. Under such conditions, however, it is difficult to maintain a stable pool of free cytosolic iron because it is constantly disturbed by oxidizing Fe^2+^ that is present as a cofactor in mononuclear iron enzymes and [4Fe-4S] clusters (Imlay, 2013). Upon oxidation, [4Fe-4S] clusters release either Fe^2+^ or Fe^3+^ (depending on the oxidant), and mononuclear iron enzymes release Fe^3+^, which, however, is converted back to Fe^2+^ by cellular reductants. This released iron further promotes oxidative damage (Keyer and Imlay, 1996; Imlay, 2013). Whether the corresponding enzymes suffer permanently from loss of activity depends on whether H_2_O_2_ or ${\text{O}}_{2}^{\mathrm{•-}}$ attacks the enzyme and in what quantity, and in addition, the composition of the active site plays an important role. A sulphur-containing ligand in the active site can quench one cycle of the Fenton reaction, but if continuously exposed to ROS, the active site will be irreparably oxidized (Anjem and Imlay, 2012). Simultaneously with the release of Fe^3+^, a small fraction of mononuclear iron enzymes without sulphur-containing ligands in the active site is irreversibly inactivated by treatment with H_2_O_2_ due to the Fenton reaction (Sobota and Imlay, 2011), whereas inactivation by ${\text{O}}_{2}^{\mathrm{•-}}$ was found to be reversible (Gu and Imlay, 2013). In addition, de-metallated mononuclear iron enzymes run the risk of being re-metallated by a non-cognate metal that confers much lower activity (*e.g*., zinc, see Section 4.2) (Gu and Imlay, 2013). Thus, excessive sequestration of free iron would impact on metabolic pathways, just as in the case when enzymes are oxidatively deprived of Fe^2+^ (and later further damaged). There is no simple solution to this paradox. Pathogens and other bacteria have evolved some strategies to suppress the deleterious behavior of iron under excessive oxidative stress conditions. One of these is that they rely, at least in part, on the replacement of iron by manganese in two different groups of enzymes discussed in Section 4.

The need for manganese as an iron substitute during oxidative stress is most evident in bacteria that do not normally rely on manganese and are sometimes referred to as iron-centric. *E. coli*, *Salmonella enterica*, and *Shigella flexneri*, like most Gram-negative bacteria, belong to the group of iron-centric bacteria. The discovery of how their key proton-dependent manganese importer (MntH) is regulated has underpinned the role of manganese as an agent against iron starvation and/or oxidative stress (Kehres et al., 2000; Anjem et al., 2009). MntH is under the control of at least three transcriptional regulators: Ferric uptake regulator (Fur), OxyR, and MntR. Fur is a canonical repressor that senses free cytosolic iron content and, when sufficient, represses subordinate genes, including *mntH*. OxyR, typical of Gram-negative bacteria, is a global regulator that controls appropriate response to oxidative stress and induces expression of *dps* in addition to *mntH* and other subordinate genes. Dps lowers the level of free cytosolic iron, ultimately leading to the inactivity of Fur, and thus acts together with OxyR to induce *mntH*. As a result, the likelihood of iron being replaced by manganese increases. Once a sufficient amount of manganese has been imported, MntR binds manganese, blocking further transcription of *mntH*. Alternatively, expression of *mntH* is attenuated when oxidative stress conditions are past (loss of OxyR induction), allowing an increase in free cytosolic iron, leading to *mntH* repression by Fur:Fe^2+^ (Kehres et al., 2000; Kehres et al., 2002; Runyen-Janecky et al., 2006; Anjem et al., 2009; Seo et al., 2015). Other bacteria may have a higher requirement for manganese to meet their specific needs, *e.g*., sporulation, iron-free lifestyle, or non-enzymatic protection against ${\text{O}}_{2}^{\mathrm{•-}}$ , some of these specific examples are briefly mentioned in Section 4.1.1 (Bosma et al., 2021). In species with a higher requirement for manganese, usually Gram-positive bacteria, the import of this metal is usually not intertwined with iron homeostasis, but occurs in a simpler manner and is regulated solely by the concentration of manganese in the cytosol (Huang et al., 2017; Bosma et al., 2021). Nevertheless, the strategy of iron sequestration by Dps seems to be ubiquitous (Orban et al., 2022), supporting the idea that during oxidative stress the free cytosolic iron concentration decreases, allowing other metals, *e.g*. manganese, to take its place in active sites. Of note, the exception are lactobacilli that accumulate manganese to extreme amounts (up to 35mM) and although encoding Dps, the Dps does not sequester iron while its DNA binding is preserved (Archibald and Duong, 1984; Orban et al., 2022).

## 4 How can manganese replace iron in enzymes?

**Figure 4:**
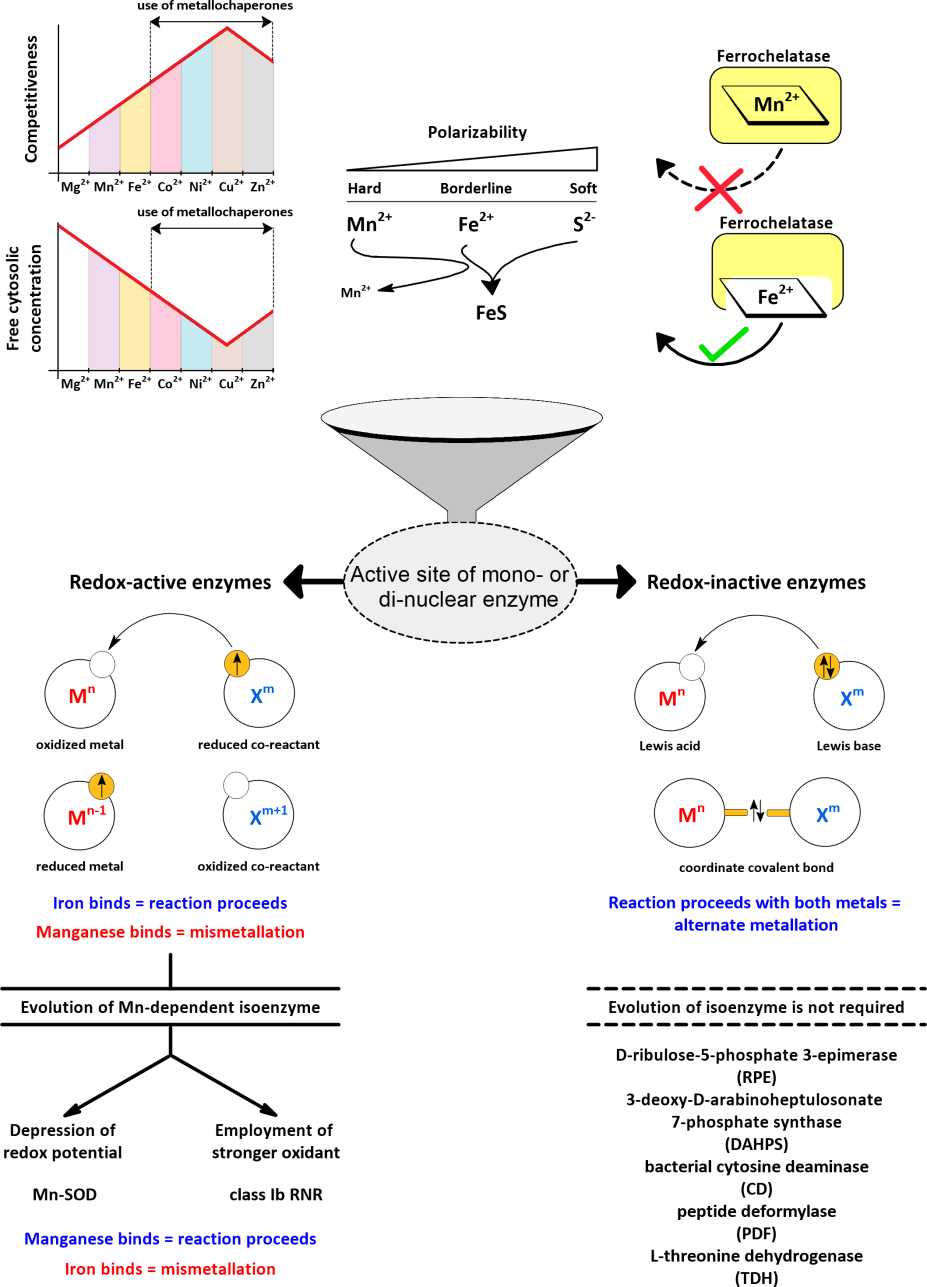
Active sites of mono- and dinuclear enzymes are susceptible to metallation by iron and manganese. The more competitive metals of the Irving-Williams series (Irving and Williams, 1948) are maintained at lower free concentrations in the cytosol by the action of metallochaperones and transport processes to allow iron and manganese to participate in the metallation of active sites (Robinson and Glasfeld, 2020). The differential polarizability of Mn^2+^ and Fe^2+^ serves to distinguish these metals by sulphur-containing ligands (Pearson, 1963). Ferrochelatase can accidentally accommodate manganese instead of iron, yet no Mn^2+^-loaded porphyrin is released (Medlock et al., 2009). In redox-inactive enzymes, both metals, iron and manganese, can support catalysis, and manganese additionally provides resistance to oxidative stress. In redox-active enzymes, manganese is not a suitable substitute for iron due to its high redox potential, so specific manganese-dependent isoenzymes need to evolve. In this review, examples of different strategies are examined for how a manganese-dependent isoenzyme overcomes the high redox potential of manganese.

Divalent metal ions generally have two biological functions: They mediate electron transfers due to changes in their oxidation state (redox-active) and/or serve as Lewis acids (redox-inactive). When a metal acts only as a Lewis acid, its oxidation state does not change, but its vacant orbitals are used to accept one or more lone pairs of electrons, which can temporarily help stabilize a negatively charged intermediate in a reaction or activate a reactant (*e.g*., a carbonyl group or a water molecule). As cofactors in enzymes, iron and manganese can play both roles. The respective mode of action, redox-active or redox-inactive (Lewis acid), defines two different ways in which manganese can replace iron and *vice versa* (**Figure 4**).

Mn^2+^ is more difficult to oxidize compared to Fe^2+^ (**Figures 1**, **2**). Consequently, Mn^2+^ is more reliable when it comes to increased oxidative stress, but it also means that for most redox-active enzymes, a simple substitution of iron by manganese will not work. However, it is known that, for example, ligands that chelate the metal have an important effect on its intrinsic redox potential (Hosseinzadeh and Lu, 2016), (Sections 4.1.1.1 and 5.2). Thus, by modulating the microenvironment of the enzyme’s active site, the redox potential of manganese can be lowered to allow its oxidation under the same steady-state conditions. Alternatively, enzyme can acclimate to a higher redox potential of manganese by using a stronger oxidant. In either case, a manganese-dependent isoenzyme must evolve to use manganese as a substitute for iron in a reaction that involves a change in the oxidation state of the metal. In this review, we focus on the isoenzymes of superoxide dismutase (SOD) and ribonucleotide reductase (RNR) in pathogens as examples of enzymes that have evolved two different strategies of how to use manganese in place of iron in a redox-active reaction (**Figure 4**). Because the incorrect metal in a particular isoenzyme leads to a severe reduction or complete loss of activity (referred to here by the term “mismetallation”), we have focused on mechanisms that might help in the insertion of the cognate metal. It appears that there are other redox-active manganese-dependent isoenzymes that may be involved in pathogenesis. Manganese-dependent catalase has recently been implicated in the pathogenesis of some bacteria (Permpoonpattana et al., 2013; Diaz-Ochoa et al., 2016; Wan et al., 2017), and some pathogens encode oxygen-dependent coproporphyrinogen III oxidase (HemF), which is sometimes present as an isoenzyme of oxygen-independent coproporphyrinogen III oxidase (HemN) (Grage, 2005). Some reports suggest that HemF may be manganese-dependent in bacteria (Breckau et al., 2003; Mancini and Imlay, 2015). Recently, redox-active enzymes containing unusual hetero-di-metal cofactors with iron and manganese ions have been identified and found to be important for *Chlamydia trachomatis* and *Mycobacterium tuberculosis*. In *C. trachomatis*, this hetero-di-metal cofactor is present in its RNR (Section 4.1.2.3) and in an oxygenase that plays a key role in the biosynthesis of the folate cofactor (Manley et al., 2022). In *M. tuberculosis*, the hetero-di-metal cofactor is harbored by an enzyme belonging to the R2-like ligand-binding oxidase (R2lox) family, which is closely related to the RNR family, but the physiological role of R2lox enzymes remains unknown (Andersson and Hogbom, 2009). Nevertheless, R2lox was identified as one of the 10 most upregulated proteins in virulence strains compared to vaccine strains of *M. tuberculosis* (Schmidt et al., 2004). What intrinsic mechanisms are required for the correct metallation of these enzymes and/or how the bacterial cell facilitates correct metallation is still under investigation (Kisgeropoulos et al., 2020). In addition to the water-oxidizing complex, several other redox-active manganese-dependent isoenzymes are known, such as homoprotocatechuate-2,3-dioxygenase, lipoxygenase, oxalate decarboxylase, and oxalate oxidase, but their involvement in pathogenesis is very unlikely (Zhu and Richards, 2017). Thus, the evolution of such enzymes seems to be a rather rare event. The explanation could be that the origin of life in an anoxic environment tailored metabolism to lower redox potentials, which are suitable for iron but extremely difficult to achieve for manganese.

But if iron or manganese serve only as Lewis acids, is non-cognate metallation still undesirable? On the contrary, we are just beginning to understand that for redox-inactive enzymes, the incorporation of manganese instead of iron is actually physiological and even beneficial in the presence of oxidative stress or iron scarcity (discussed in Section 4.2). In redox-inactive reactions, the redox potential differences between iron and manganese do not affect the reaction, so that direct exchange of these metals is possible with retention of full or substantial part of the catalytic activity, provided, however, that sulphur-containing ligands are not dominant in the coordination of the metal (see below). “Mismetallation” is a suitable term for redox-active enzymes because its negative undertone implies a loss of activity, but for redox-inactive enzymes we suggest that “alternate” metallation would be a more appropriate term. In order to use manganese in place of iron in redox reactions, manganese-dependent isoenzymes had to evolve, but can we specify what type of redox-inactive enzymes that rely on iron should physiologically benefit from the alternate metallation with manganese?

As far as we know, all heme-containing enzymes are redox-active, which precludes this group of enzymes from being alternately metallated without losing their function. Any metal other than iron, if it can be incorporated into the porphyrin ring by ferrochelatase, will result in mismetallated enzymes (Dailey, 1987; Labbé et al., 1999; Majtan et al., 2011). This narrows down the possible candidates for alternate metallation into two groups of enzymes that can use iron as a Lewis acid: Enzymes with iron-sulphur clusters or with iron ions coordinated only by amino acid residues (mono- and dinuclear iron enzymes). Iron in iron-sulphur clusters is coordinated predominantly by sulphur ligands in the form of sulfide or thiolate; in addition, iron is supplied to the nascent iron-sulphur clusters already complexed by thiolates (*e.g*., IscA) (Yang et al., 2006). Pearson developed the concept called Hard and soft acids and bases (HSAB), which classifies sulfides and thiolates as soft (polarizable) Lewis bases, Fe^2+^ as a borderline acid, and Mn^2+^ as a hard (non-polarizable) acid (Pearson, 1963; Jensen, 1978). According to the HSAB concept, soft bases tend to bind to soft acids and hard acids tend to bind to hard bases, so sulfide or thiolate choose the softer acid Fe^2+^. It should be noted, however, that the effects of sulphur-containing ligands are additive and therefore single cysteine in the active site does not generally favor either metal, as we are aware of examples where manganese was able to replace iron in the active site with single cysteine residue (Anjem and Imlay, 2012). Using the iron-specific repressor (DtxR) from *Corynebacterium diphtheriae*, it was shown that mutation of two conserved sulphur-containing ligands to acidic residues suppresses selectivity toward Fe^2+^ in favor of Mn^2+^ (Guedon and Helmann, 2003). Consistent with the HSAB concept, a data mining study suggests that cysteine is very rare in active sites coordinating Mn^2+^ (Zheng et al., 2008). Other functional groups in coordinating residues containing nitrogen or oxygen are classified as borderline to hard bases and do not seem to show obvious preferences (Zheng et al., 2008). In summary, iron-sulphur enzymes are inherently resistant to manganese binding mainly due to the combined selectivity of sulphur-containing ligands.

Overall, when considering manganese as an iron substitute, only redox-inactive mono- and dinuclear iron enzymes are plausible candidates to benefit from alternate metallation (**Figure 4**). One might think that the extent of such substitution is marginal, since iron is not frequently reported to play the role of a Lewis acid. Zinc or magnesium are more common Lewis acids, and the latter is often shown to be substitutable by manganese (Andreini et al., 2008). However, iron can easily oxidize during aerobic purification processes, resulting in its dissociation from the active site and its replacement by other aerobically stable metals (*e.g*., zinc). For the same reason, iron may appear to have low activity under aerobic conditions in kinetic assays (Rajagopalan and Pei, 1998; Imlay, 2014). Therefore, it is reasonable to consider the use of iron as a Lewis acid in mono- and dinuclear enzymes to be underestimated and, consequently, also the replacement of iron with manganese in these enzymes. Moreover, alternate metallation does not require isoenzymes whose existence would be conspicuous in genomes, and thus the full extent of this process is probably not fully known.

### 4.1 Substitution of iron by manganese in redox-active enzymes

#### 4.1.1 Superoxide dismutase

Convergent evolution of three unrelated enzymes has resulted in three distinct families of SODs: the nickel (Ni-) SODs; the copper-only (Cu-) and copper-zinc (CuZn-) SODs; and the iron (Fe-) and manganese (Mn-) SODs (Frye et al., 2022). SODs carry out the disproportionation of 2 ${\text{O}}_{2}^{\mathrm{•-}}$ to O_2_ and H_2_O_2_, a clever approach in aerobic environments since no cellular reductants are consumed, unlike superoxide reductases (SORs) that perform the reduction of ${\text{O}}_{2}^{\mathrm{•-}}$ at the expense of NADH. In the case of SORs, no O_2_ is generated and the second ${\text{O}}_{2}^{\mathrm{•-}}$ is not needed to complete the conversion; also, it should be noted that at low O_2_ tension cellular reductants are not in short supply. This comparison should indicate that SORs are more suited to microaerophilic environments, whereas SODs are most effective under aerobic conditions, and thus these two enzymes are generally not found simultaneously in the bacterial cytosol (Martins et al., 2019). CuZn-SODs are used in the cytosol of eukaryotes but not in the cytosol of prokaryotes, which translocate them to the periplasm (Gram-negative bacteria) or secrete them (Gram-positive bacteria), Cu-SODs appear to be extracellular only, and Ni-SODs are found mainly in the cytosol of marine prokaryotes (Wu et al., 1998; Robinett et al., 2018; Sutherland et al., 2021; Frye et al., 2022). From a pathogen perspective, Fe-SOD and Mn-SOD are the only cytosolic enzymes that eliminate ${\text{O}}_{2}^{\mathrm{•-}}$ under aerobic conditions and will therefore be the focus here.

Fe-SOD is probably the oldest; SODs using other metals emerged later (Case, 2017), probably in response to declining iron concentrations (see Introduction). Mn-SOD evolved directly from Fe-SOD, and thus the example of Mn-SOD can be used to demonstrate what it means to replace an iron-dependent redox-active enzyme with a manganese-dependent counterpart.

##### 4.1.1.1 Mn-SOD exerts stronger depression of redox potential on bound metal

**Figure 5:**
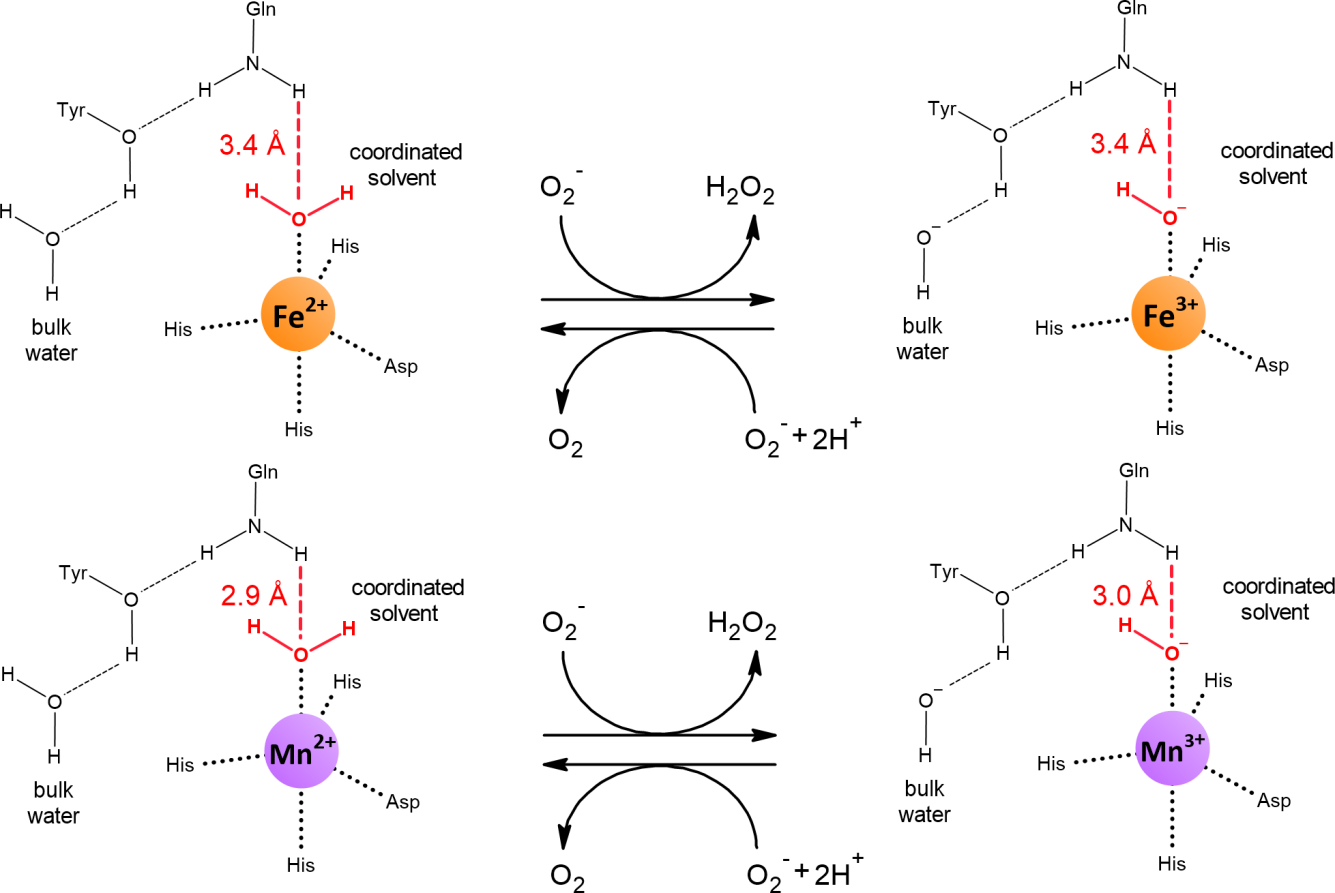
Reduced and oxidized states of Fe-SOD and Mn-SOD. Dashed lines represent hydrogen bonds. The amide group of the side chain of the conserved glutamine is a hydrogen bond donor for the coordinated solvent (in red). The shorter the hydrogen bond, the more the redox potential of the bound metal decreases. The distances were obtained from the crystal structures of *E. coli* Fe^2+^-SOD (PDB: 1ISA), Fe^3+^-SOD (PDB: 1ISB), Mn^2+^-SOD (PDB: 1IX9) and Mn^3+^-SOD (PDB: 1IXB).

The redox potential of manganese is higher than that of iron (**Figure 2**). Therefore, the active site of Mn-SOD must lower the redox potential of manganese to the similar value that iron has in Fe-SOD for both isozymes to perform the same reaction. At first glance, it is difficult to figure out how Mn-SOD achieves the effect of lowering the redox potential of manganese because the active sites of Fe-SOD and Mn-SOD are virtually superimposable. It is important that one of the ligands coordinating the metal is a hydroxide or a water molecule. During the catalytic cycle, this solvent molecule donates a proton and accepts a proton. Whether a hydroxide or a water molecule is present at any given time is directly related to the oxidation state of the metal (**Figure 5**). The hydroxide lowers the redox potential of the metal and therefore promotes the +3 oxidation state, while the metal tends to have the +2 oxidation state when a water molecule is present as a ligand. This important solvent molecule is anchored by the coordinate bond with the metal and by the hydrogen bond extending from the amide group of the side chain of the glutamine residue. The shorter the hydrogen bond between glutamine and the solvent molecule, the more easily the hydroxide is formed and the more the redox potential of the metal is lowered. In accordance with the need for a stronger reduction of the redox potential of manganese, the solvent-coordinating glutamine is located closer to the solvent molecule in Mn-SOD than in Fe-SOD. As a result, the redox potentials of both enzymes are closer together, 0.3 V for Mn-SOD and 0.1 V for Fe-SOD in *E. coli*, than would be the case for free ions in aqueous solution (**Figure 2**). The position of the solvent-coordinating glutamine is fixed, so that when iron is bound instead of manganese, the same redox tuning applies. However, since iron already has a naturally low redox potential, further depression causes the redox potential of iron in Mn-SOD to be so low that the Fe^2+^ oxidation state can no longer be reached. Similarly, when manganese is bound instead of iron in Fe-SOD, the solvent-coordinating glutamine is too far away to sufficiently support stabilization of the hydroxide, so that the Mn^3+^ oxidation state is inaccessible (Vance and Miller, 2001; Miller, 2008; Miller, 2012).

**Figure 6:**
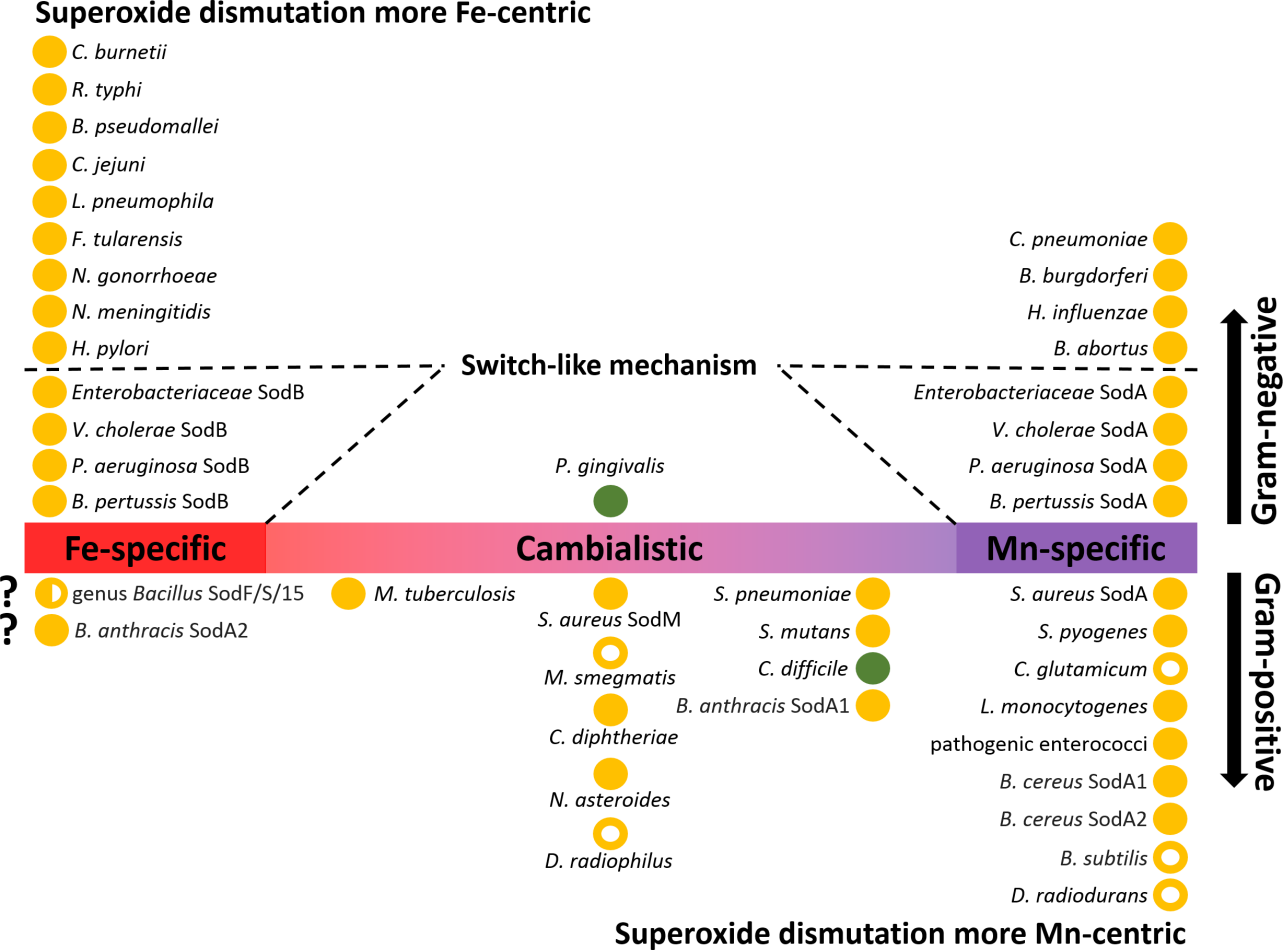
Distribution of Fe/Mn family superoxide dismutases (SODs) in Gram-negative and Gram-positive bacteria. SODs present in bacterial pathogens (closed circles) and selected non-pathogenic bacteria (open circles) are ranked according to their metal specificity (iron-specific, manganese-specific and cambialistic). If bacteria or groups of bacteria possess more than one SOD isoenzyme, these are indicated after the name of the species. Aerobic bacteria are depicted in orange, while strict anaerobes are depicted in green. Gram-negative bacteria that use a switch-like riboregulatory mechanism are depicted with dashed lines. In addition to the species mentioned in the text, the following bacterial species that code for SOD enzymes are shown in the figure: *Neisseria gonorrhoeae* and *N. meningitidis* (Archibald and Duong, 1986; Seib et al., 2004), *Haemophilus influenzae* (Kroll et al., 1998), *Porphyromonas gingivalis* (Hiraoka et al., 2000), *Coxiella burnetii* (Heinzen et al., 1992), *Rickettsia typhi* (McLeod et al., 2004), *Burkholderia pseudomallei* (Loprasert et al., 2000), *Campylobacter jejuni* (Atack and Kelly, 2009), *Legionella pneumophila* (Sadosky et al., 1994), *Francisella tularensis* (Bakshi et al., 2006), *Helicobacter pylori* (Ernst et al., 2005), *Chlamydia pneumoniae* (Yu et al., 2004), *Borrelia burgdorferi* (Aguirre et al., 2013), *Brucella abortus* (Sriranganathan et al., 1991), *Streptococcus pneumoniae*, *S. mutans*, and *S. pyogenes* (Gerlach et al., 1998; De Vendittis et al., 2010; Eijkelkamp et al., 2014), *Mycobacterium tuberculosis* and *M. smegmatis* (Yamakura et al., 1995; Bunting et al., 1998; Padilla-Benavides et al., 2013), *Corynebacterium diphtheriae* (Britton et al., 1978), and *C. glutamicum* (El Shafey et al., 2008), *Listeria monocytogenes* (Britton et al., 1978) *Enterococcus faecalis* (Verneuil et al., 2006; Wasselin et al., 2021), *Nocardia asteroides* (Beaman et al., 1983), and *Deinococcus radiophilus* (Yun and Lee, 2004).

SODs from the Fe/Mn family are not always restricted to the use of iron or manganese. Strict specificity can be seen as an extreme case of evolution where catalytic activity has been maximized and flexibility has been completely sacrificed for specific metal use. However, between the strictly iron- and manganese-dependent SODs, there is a whole range of less catalytically active SODs that show some preference for one of the two metals or whose activity is the same for both metals. These SODs are termed cambialistic (**Figure 6**) (Sheng et al., 2014; Krauss et al., 2015; Barwinska-Sendra et al., 2020). Using two isozymes of *Staphylococcus aureus*, one cambialistic and one manganese-dependent, it was shown that their metal specificities (and thus their redox tuning) can be interchanged by the mutual exchange of two nonpolar residues in close spatial proximity to the active site. In the same study, no changes were observed in the hydrogen bonds coordinating the solvent molecule (Barwinska-Sendra et al., 2020).

##### 4.1.1.2 Mn-SOD requires that bacteria facilitate its correct metallation

The case of specialized SODs shows that proper metallation is crucial for the function of a redox-active enzyme, as was generally noted in Section 4. However, this also implies that without specific means to provide the cognate metal and without selective intrinsic affinity for the cognate metal, two redox-active isoenzymes that rely on different metals cannot be expressed simultaneously in one cell compartment. In bacteria, this appears to be a problem, especially for the iron/manganese pair in Fe/Mn-SOD metallation (Mizuno et al., 2004; Aguirre and Culotta, 2012). Correct metallation of SODs is critical because the binding of the metal to SOD is essentially irreversible and thus incorrect metal cannot be replaced, leading to permanent inactivation of the enzyme (Whittaker et al., 2006).

**Figure 7:**
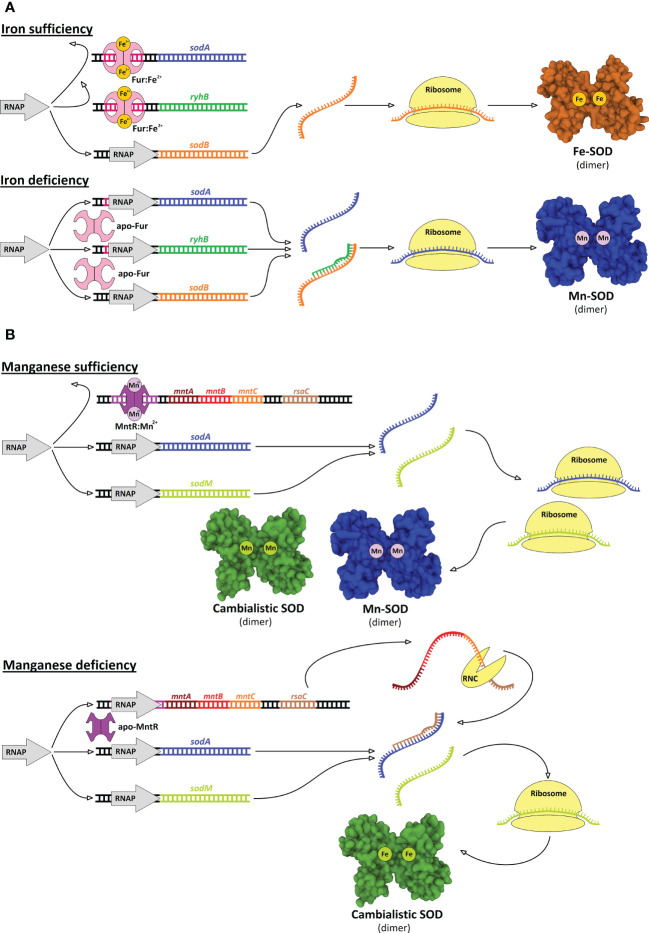
Two distinct mechanisms based on transcriptional and post-transcriptional regulatory mechanisms controlling expression of SODs. **(A)** In *E coli* and several other Gram-negative bacteria, small RNA RyhB and availability of iron play a central role in control of alternate expression of Fe-SOD and Mn-SOD. When iron is sufficient, Fur repressor loaded with iron inhibits expression of *ryhB* gene and *sodA* encoding Mn-SOD, while *sodB* gene encoding Fe-SOD is transcribed by RNA polymerase (RNAP) and Fe-SOD is produced. When iron is at scarce, apo-Fur does not repress *ryhB* and *sodA*, hence Mn-SOD is produced. Simultaneously, transcribed RyhB binds to *sodB* transcript and blocks its translation, thus, Fe-SOD is not produced (Masse and Gottesman, 2002; Vecerek et al., 2003). **(B)** In Gram-positive *S. aureus* small RNA RsaC and manganese availability control expression of *sodA* and *sodM* genes. When manganese is sufficient, MntR repressor loaded with manganese inhibits expression of *mntABC* operon including *rsaC* gene, while both *sodA* and *sodM* genes, encoding Mn-SOD and cambialistic SOD, respectively, are expressed and respective SODs are produced. When manganese is at scarce, apo-MntR does not repress *mntABC* operon including *rsaC*, note that RsaC is cleaved out from 3’-end of the operonic transcript by RNase III (RNC). RsaC binds to *sodA* transcript and inhibits its translation so that only cambialistic SOD is produced (Lalaouna et al., 2019).

Typically, Gram-negative bacteria have low manganese requirements (sometimes referred to as iron-centric) (Bosma et al., 2021) but some of these bacteria have both SOD isoenzymes (**Figure 6**). It is likely that both the Fe-SOD and Mn-SOD isoforms confer greater robustness to pathogens in the face of metal fluctuations, especially when exposed to excessive oxidative stress. However, the advantage of having two SOD isoforms only comes into play if they can be expressed alternately depending on the free cytosolic Fe:Mn ratio. Indeed, a riboregulatory switch-like mechanism has been elucidated in *E. coli* by which the bacterium turns off the expression of Fe-SOD while inducing the expression of Mn-SOD (**Figure 7A**). The switch consists of the Fur repressor and a *trans*-encoded small RNA RyhB (Masse and Gottesman, 2002). Under iron-rich conditions, *sodB* encoding Fe-SOD is expressed, and Fe^2+^-loaded Fur prevents transcription of RyhB and s*odA* gene encoding Mn-SOD. Under iron-limiting conditions, apo-Fur does not repress the expression of either RyhB or *sodA*. Expressed RyhB binds to *sodB* mRNA, and the resulting RNA duplex is degraded, leading to inhibition of *sodB* translation (Vecerek et al., 2003; Afonyushkin et al., 2005). Simultaneously, the suppression of *sodA* is abolished, leading to the resumption of SOD activity in the cell (Masse et al., 2005; Jacques et al., 2006). Analogous switch-like mechanisms enable the transition from iron- to manganese-dependent SOD activity in several pathogenic species (**Figure 6**), including *S.* Typhimurium (Tsolis et al., 1995), *S. flexneri* (Oglesby et al., 2005), *S. dysenteriae* (Murphy and Payne, 2007), *Klebsiella pneumoniae* (Huang et al., 2012; Najmuldeen et al., 2019), *Vibrio cholerae* (Kimoto et al., 2001; Mey et al., 2005), *Yersinia pestis* (Pieper et al., 2010), and *Pseudomonas aeruginosa* (Hassett et al., 1997; Reinhart et al., 2015). In *Bordetella pertussis*, the isoenzymes Fe-SOD and Mn-SOD have been experimentally identified, and *sodA* expression has been found to be regulated by Fur (DeShazer et al., 1994; Graeff-Wohlleben et al., 1997), suggesting that this mechanism also functions in this re-emerging pathogen. On the other hand, there are numerous pathogens that do not rely on the switch and encode single cytosolic SOD, with most pathogens encoding Fe-SOD rather than Mn-SOD (**Figure 6**), consistent with the low requirement for manganese in Gram-negative bacteria. It would be interesting to see whether Fe/Mn-SODs from Gram-negative pathogens containing only one isoform have not evolved at least minor cambialism, similar to Gram-positive bacteria (see below).

A riboregulatory mechanism, frequently present in Gram-negative bacteria, can be found also in Gram-positive bacteria, for example, in *S. aureus* (see below). In most Gram-positive pathogens, Fe-SOD is not present while Mn-SOD is preserved. But, unlike Gram-negative pathogens, it has not achieved strict selectivity for manganese and rather retains some activity with iron to varying degrees (cambialism), presumably to avoid losing SOD activity in the face of manganese scarcity (**Figure 6**). Consistent with this strategy, Gram-positive bacteria appear to be naturally more dependent on manganese (Bosma et al., 2021) and thus the Fur binding site is not located upstream of *sodA* genes, so the Mn-SOD is the housekeeping isoenzyme and its expression may instead be controlled by MntR (Harvie et al., 2005; Wang et al., 2011; Lalaouna et al., 2019; Peng et al., 2021). In *S. aureus*, the *sodA* gene was duplicated and evolved into the second gene (*sodM*) encoding SOD, which is cambialistic (Barwinska-Sendra et al., 2020). How the expression of *sodA* and *sodM* is controlled in *S. aureus* was explained by the identification of a *trans*-encoded small RNA called RsaC located at the 3’-UTR of the polycistronic operon encoding the manganese importer (**Figure 7B**). The entire operon (RsaC + manganese importer) is under the control of MntR. In manganese-deficient *S. aureus*, MntR abolishes repression of the locus, and RsaC is expressed, processed, and in turn pairs with *sodA* mRNA, thereby blocking Mn-SOD translation (Lalaouna et al., 2019). The RsaC-mediated mechanism is more straightforward compared to the RyhB-mediated mechanism because Mn-SOD is formed in the presence of manganese and not only in the absence of iron; moreover, unlike Fe-SOD, cambialistic SodM expression does not need to be turned off.

The complicated composition of SODs and their physiological functions are found in the genus *Bacillus*. Members of the pathogenic *B. cereus* group (*B. cereus*, *B. anthracis*, and *B. thuringiensis*) have an additional SOD isoform compared with nonpathogenic species, *e.g*., *B. subtilis*. This additional isoenzyme is encoded by the s*odA2* gene. Otherwise, all members of the genus *Bacillus* possess a *sodA1* gene, gene encoding SOD from the Fe/Mn family (*e.g*., *sodF*, *sodS*, *sod15*), which is thought to use iron, and finally *sodC* encoding CuZn-SOD, which, in *B. subtilis*, has lost its original function (Inaoka et al., 1999; Banci et al., 2005; Passalacqua et al., 2006; Wang et al., 2011; Zhang et al., 2020). Based on the sequence similarity and resistance to H_2_O_2_ treatment, some studies suggested that *sodA1* and *sodA2* are coding for Mn-SOD (Wang et al., 2007; Zhang et al., 2020); however, Tu et al. (2012) showed in *B. anthracis* that SodA1 can utilize iron to a lesser extent, but the highest activity is achieved with manganese, and that in their experiments (performed in iron-rich LB media), SodA2 only binds iron. Surprisingly, the authors failed to metallate apo-SodA2 with manganese, so they could not assess the activity of SodA2 with manganese. Whether SodA2 is actually Fe-SOD or whether it is a cambialistic SOD is still an open question. The rich arsenal of SODs in the genus *Bacillus* is used during sporulation. Spores are highly resistant to external perturbations and although they are considered biochemically dormant, the outermost layer, the spore coat, contains several enzymes. It has long been known that the addition of manganese triggers and accelerates sporulation (Charney et al., 1951; Vasantha and Freese, 1979). Recent analyzes of *B. anthracis* and *B. cereus* spores revealed that mainly Fe/Mn family SODs are localized on their surface, and knock-out experiments also showed compensatory effects for missing SOD/s by the remaining isoforms including CuZn-SOD (Cybulski et al., 2009; Zhang et al., 2020). Of note, Mn-SOD in *B. subtilis* also associates with the spore coat (Henriques et al., 1998). *Clostridium difficile* encodes only one Mn-SOD with mild cambialistic feature (Li et al., 2014; Li et al., 2015). Similar to members of the genus *Bacillus*, Mn-SOD has been detected in *C. difficile* on the surface of its spores (Permpoonpattana et al., 2013).

##### 4.1.1.3 SOD activity can be substituted by low molecular weight manganese complexes

Despite the crucial importance of cytosolic SOD enzymes, some representatives of the lactic acid bacteria do not possess these enzymes but can grow aerobically (Archibald and Fridovich, 1981). Furthermore, the important pathogen *N. gonorrhoeae* contains only Fe-SOD, but this has been shown to be poorly expressed *in vitro*, presumably due to the abundant manganese in the medium (Tseng et al., 2001). These species employ an unusual strategy to compensate for the lack of SOD activity in the cytosol by accumulation of manganese to high intracellular levels. Moreover, it has been shown that bacteria that normally possess Mn-SOD, when deprived of this enzyme, also accumulate manganese in the corresponding mutants (Inaoka et al., 1999; Al-Maghrebi et al., 2002). Loss of cytoplasmic SOD in *Saccharomyces cerevisiae*, the CuZn-SOD isoform, results in aerobic growth defects that can be corrected by increased intracellular manganese (Lapinskas et al., 1995; McNaughton et al., 2010). Indeed, Mn^2+^ in complexes with phosphate has been shown *in vitro* to disproportionate ${\text{O}}_{2}^{\mathrm{•-}}$ in a manner similar to SOD (Barnese et al., 2012). Although *Deinococcus radiodurans* has Mn-SOD, it appears to use the strategy of accumulation of manganese complexes to further passively enhance its protection against oxidative stress, which contributes to *D. radiodurans*’ resistance to irradiation (Daly et al., 2004). The nonenzymatic removal of ${\text{O}}_{2}^{\mathrm{•-}}$ by low molecular weight manganese complexes is an intriguing biological adaptation that allows circumvention of the need for cytoplasmic SOD activity and may indicate how microbes survived oxygenation on Earth before evolving their own enzymatic defenses. Nevertheless, in bacteria that naturally possess Mn-SOD, caution should be exercised in determining whether *in vitro* or genetically induced hyperaccumulation of manganese is relevant to the situation *in vivo* in wild-type strains. The accumulation of Mn^2+^ must be compensated by accumulation of a negatively charged counterion in order to preserve osmotic pressure and electro-neutrality of the cells (McNaughton et al., 2010). While phosphates were generally reported to compensate accumulated manganese, *D. radiodurans* accumulates small peptides (Daly et al., 2010). This and other aspects such as flexibility of expression, higher catalytic turnover (Anjem et al., 2009), much lower requirement for manganese, and lower risk of mismetallation of other redox-active enzymes and metalloregulatory proteins by manganese suggest that enzymatic elimination of ${\text{O}}_{2}^{\mathrm{•-}}$ is superior to the somewhat cumbersome nonenzymatic defense strategy, as reflected by the near-ubiquitous presence of cytosolic SODs in contrast to the rare strategy of abnormally high intracellular manganese.

#### 4.1.2 Ribonucleotide reductase

**Figure 8:**
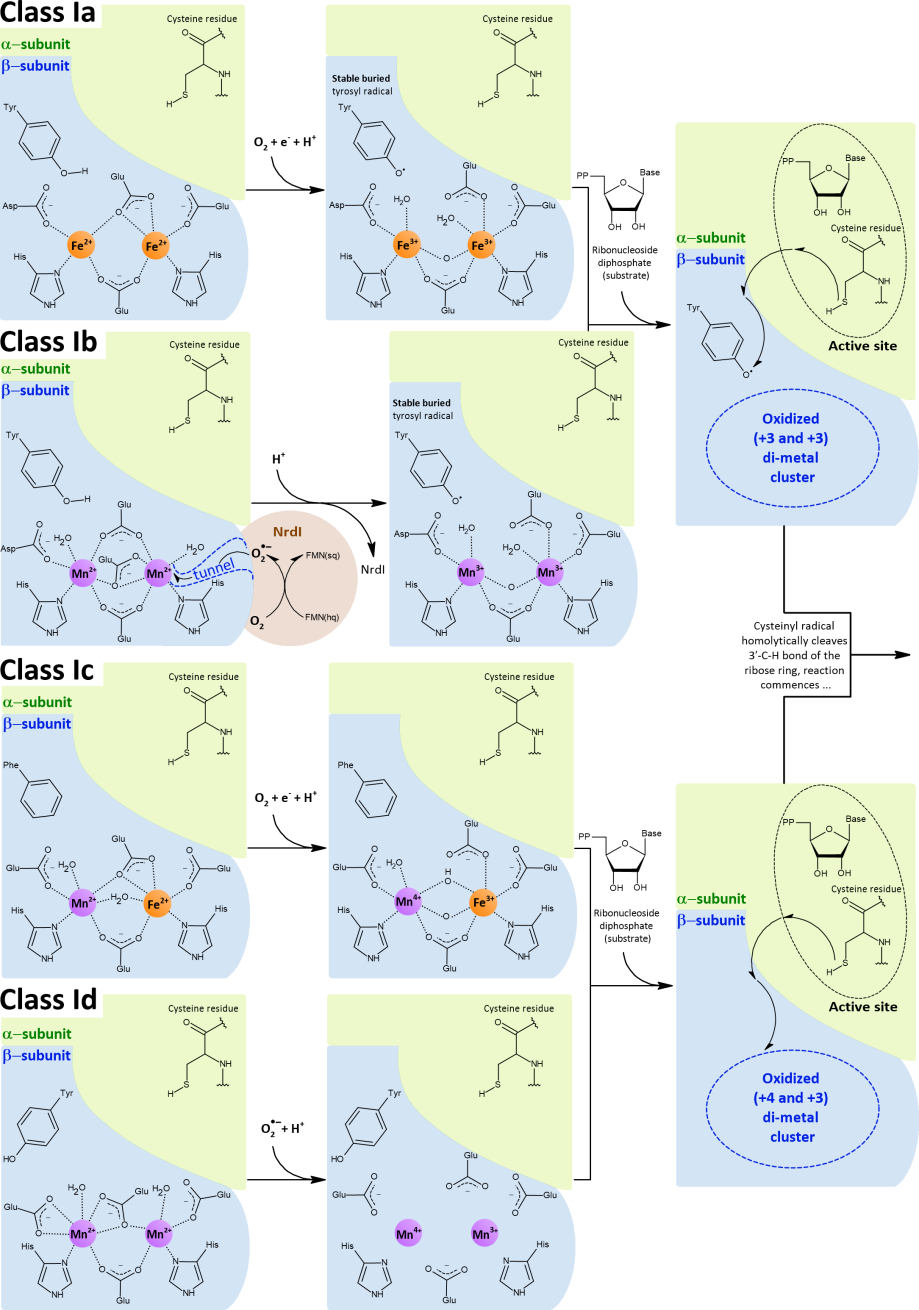
Class I RNRs operate aerobically only and have two integral subunits: the α-subunit, which contains the active site with catalytically crucial cysteine residue, and the β-subunit, which contains the di-metal cluster that generates the cysteine-oxidizing radical. Depending on the subclass, the di-metal cluster is occupied by either two iron ions (Ia), two manganese ions (Ib, Id) or by one iron and one manganese ions in a hetero-di-metal cluster (Ic). The di-iron (Ia) and hetero-di-metal (Ic) clusters are oxidized by external O_2_, whereas the di-manganese clusters (Ib, Id) are oxidized by ${\text{O}}_{2}^{\mathrm{•-}}$ . In the case of class Ib, ${\text{O}}_{2}^{\mathrm{•-}}$ is generated by the flavoprotein NrdI and is subsequently directed to the cluster through a closed pathway. Class Id was proposed to scavenge ${\text{O}}_{2}^{\mathrm{•-}}$ from the cytosol. In classes Ia and Ib, the oxidized di-metal cluster abstracts one electron from tyrosine residue and, concomitantly, a proton is transferred away from this tyrosine residue. Consequently, a tyrosyl radical is formed. This tyrosyl radical is buried deep inside the β-subunit and inaccessible to O_2_. Once the substrate is bound in the α-subunit, the radical is transferred from the tyrosine over a long distance *via* several other residues (represented by the arrows) and finally lands on the catalytic cysteine, which can finally initiate the reaction (Cotruvo and Stubbe, 2011; Cotruvo et al., 2013). In class Ic, phenylalanine replaces the tyrosine residue, and thereby the radical formally resides directly on the hetero-di-metal cluster (Hogbom et al., 2004; Jiang et al., 2007). Also in class Id, the radical formally resides on the di-metal cluster, however the tyrosine residue is present but oriented away from this cluster (Rose et al., 2018). The di-metal clusters of Ia and Ib were drawn based on *E. coli* crystal structures (PDB: 1PIY, 1MXR, 3N37), except for the oxidized di-manganese cluster, which was drawn based on the structure of *Corynebacterium ammoniagenes* (PDB: 3MJO) (Hogbom et al., 2003; Voegtli et al., 2003; Boal et al., 2010; Cox et al., 2010). Reduced class Ic hetero-di-metal cluster was drawn based on the *C. trachomatis* crystal structure (PDB: 4M1I), whereas oxidized class Ic hetero-di-metal cluster is adapted from measurements and calculations performed in Kwak et al. (2013). Reduced class Id di-manganese cluster was drawn based on the *Leeuwenhoekiella blandensis* crystal structure (PDB: 6SF5), to our knowledge the crystal structure of the oxidized state has not been reported.

Ribonucleotide reductases (RNRs) are an important group of enzymes because they convert ribonucleoside tri(di)phosphates into corresponding 2’-deoxyribonucleosides, the building blocks of DNA. All RNRs share the same structural fold of their α-subunits, where the active site containing the catalytically important cysteine residue is located. This residue is converted into a cysteinyl radical, which in turn homolytically cleaves the 3’-C-H bond of the ribose ring. The intermediate thus activated is dehydrated and subsequently reduced by two electrons, yielding the desired product. The crucial cysteinyl radical is generated by another radical. The mechanism of how a cysteine-oxidizing radical is formed (activation) is what distinguishes each class of RNRs (I, II, and III). Importantly, all members of class I RNRs are O_2_-dependent and can be divided into five subclasses, Ia, Ib, Ic, Id, and Ie. In subclasses Ia-Id (**Figure 8**), a di-metal cluster is used to generate a cysteine-oxidizing radical, whereas subclass Ie has been proposed to be metal-independent. Enzymes of subclass Ia contain a di-iron cluster (NrdA - α-subunit, NrdB - β-subunit) and enzymes of subclass Ib contain a di-manganese cluster (NrdE - α-subunit, NrdF - β-subunit) (Ruskoski and Boal, 2021). Subclasses Ia and Ib are the most common and provide a good example of how manganese can be used in place of iron in an isoenzyme. The other subclasses (Ic, Id, Ie) are less studied and are therefore mentioned here only in passing (Section 4.1.2.3).

##### 4.1.2.1 Class Ib RNRs use a stronger oxidant to overcome the high redox potential of manganese

Similar to SODs, class Ia and class Ib RNRs represent two groups of isoenzymes that catalyze the same reaction, using either iron (class Ia) or manganese (class Ib). Unlike specialized Mn-SODs, class Ib RNRs can function with iron *in vitro*, and this ability long obscured the fact that manganese is actually the cognate metal (Cotruvo and Stubbe, 2012). How then is it possible that manganese can be used for the same reaction? The di-metal cluster is critical for activation (formation of the cysteine-oxidizing radical, **Figure 8**), which is decoupled from the reaction with the ribonucleoside diphosphate itself. The separate activation step in the case of class I RNRs allows the use of different strategies involving different metals to generate the cysteine-oxidizing tyrosyl radical. In the activation step, the di-metal cluster serves to oxidize the hydroxyl group of the tyrosine residue, but prior to this task, the di-metal cluster must be oxidized. Because of the intrinsic redox potential difference between iron and manganese (**Figure 2**) and since the lowering of the redox potential of manganese is not possible in class Ib RNRs, a stronger oxidant must be used. While class Ia RNRs use O_2_ (*E*°’ = -0.18 V) to oxidize their di-iron cluster, class Ib members rely on ${\text{O}}_{2}^{\mathrm{•-}}$ (*E*°’ = +0.91 V) to oxidize their di-manganese cluster. Indeed, the manner in which the oxidant ${\text{O}}_{2}^{\mathrm{•-}}$ is generated was an important breakthrough in establishing that manganese is a cognate metal for class Ib RNRs. A flavoprotein NrdI binds to the β-subunit (**Figure 8**), reduces O_2_ to ${\text{O}}_{2}^{\mathrm{•-}}$ , and ${\text{O}}_{2}^{\mathrm{•-}}$ is subsequently channeled through a tunnel directly to the di-manganese cluster (Cotruvo and Stubbe, 2011; Zhang and Stubbe, 2011; Cotruvo et al., 2013).

##### 4.1.2.2 Cytosolic environment and likely intrinsic properties of RNRs contribute to proper metallation

It is known that iron-loaded class Ib RNRs have lower activity than a native configuration with manganese and NrdI (Cotruvo and Stubbe, 2012). Although the class Ib RNR of *S. sanguinis* is only 3.5-fold less active with iron compared with manganese *in vitro*, aerobic growth and virulence are completely abolished after deletion of the *nrdI* gene (Makhlynets et al., 2014; Rhodes et al., 2014), and Martin and Imlay (2011) showed that the class Ib RNR of *E. coli* cannot support growth when manganese is not available. Nevertheless, other activities such as DNA repair require the supply of 2’-deoxyribonucleosides, and thus we speculate that even a mismetallated class Ib RNR with lower activity is better than no activity at all. Gram-positive pathogens starving for manganese, for example, might survive for a time without dividing until they regain access to manganese. Conversely, it has been observed *in vitro* that similar to class Ib RNRs, class Ia RNRs can also be mismetallated, but in this case with complete loss of activity (Atta et al., 1992; Cotruvo and Stubbe, 2011). No metallochaperones are known to control accommodation of the correct metals. Altogether, these observations raise the urgent question of how mismetallation is suppressed *in vivo*.

**Figure 9:**
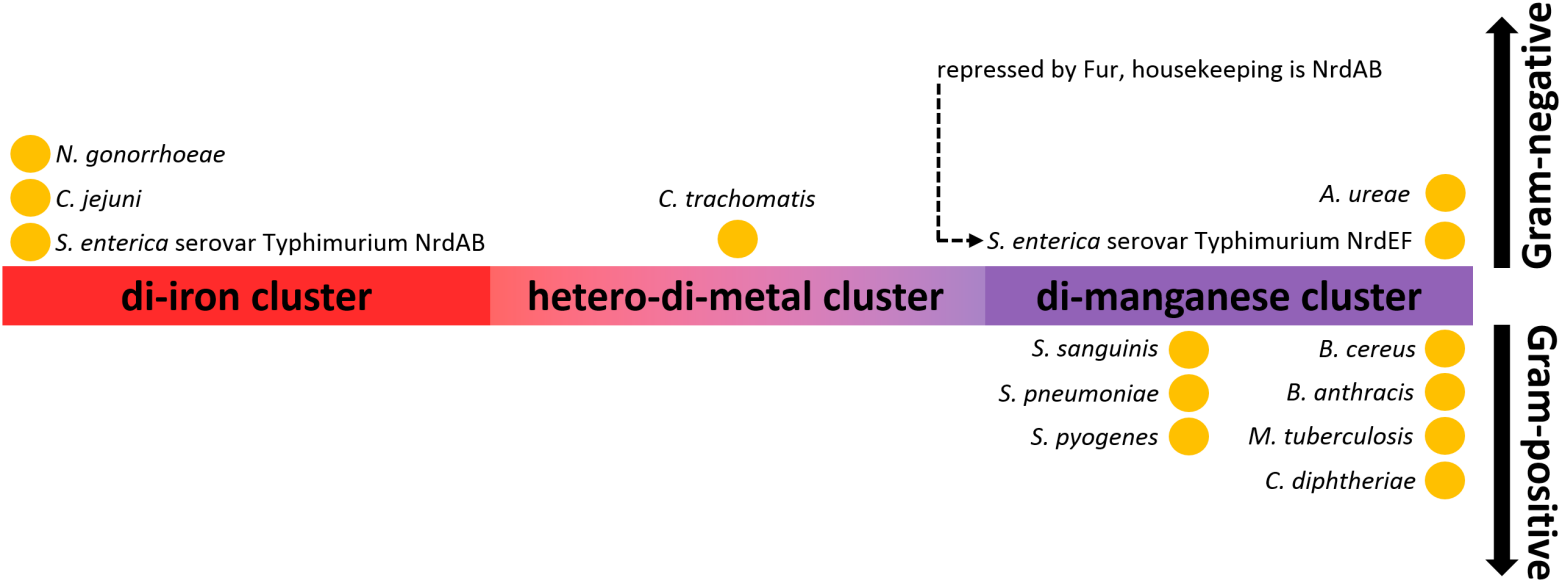
Distribution of class I RNRs based on their metal-dependency in pathogens. *N. gonorrhoeae* (Narasimhan et al., 2022), *C. jejuni* (Alqurashi et al., 2021), *S.* Typhimurium (Eriksson et al., 1998; Cotruvo and Stubbe, 2010; Panosa et al., 2010; Cotruvo and Stubbe, 2011; Martin and Imlay, 2011), *C. trachomatis* (Jiang et al., 2007), *A. ureae* (Rose et al., 2019), *S. sanguinis* (Makhlynets et al., 2014), *S. pneumoniae* (Jayachandran et al., 2021), *S. pyogenes* (Roca et al., 2008)*, B. cereus* (John et al., 2022), *B*. *subtilis* (Zhang and Stubbe, 2011)*, B. anthracis* (Grāve et al., 2020)*, M. tuberculosis* (Hammerstad et al., 2014). Although the RNR of *C*. *diphtheriae* has not been directly characterized, it is very likely that it is manganese-dependent, as two close non-pathogenic relatives, *C*. *glutamicum* and *C*. *ammoniagenes*, have been demonstrated to be manganese-dependent RNRs (Abbouni et al., 2009; Cox et al., 2010).

To date, only a handful of RNRs have been well-characterized with respect to their cognate metal, but their distribution may indicate that Gram-positive pathogens prefer the manganese-dependent isoform, whereas Gram-negative pathogens prefer the iron-dependent isoform (**Figure 9**). The classification into Gram-negative and Gram-positive pathogens follows the same pattern as in the case of SODs, *i.e*., typical Gram-negative bacteria have a lower requirement for manganese (iron-centric) compared with Gram-positive bacteria (Bosma et al., 2021). Among pathogens, only members of the *Enterobacteriaceae* appear to have both iron- and manganese-dependent isoforms. In the case of *Enterobacteriaceae*, the Fur-binding site is located upstream of the operon encoding class Ib RNR, so similar to Mn-SOD, expression of class Ib RNR occurs only in the presence of iron scarcity, minimizing mismetallation (Martin and Imlay, 2011). Surprisingly, a riboregulatory mechanism functionally comparable to the action of RyhB has not been described, so that class Ia RNR expression in *E. coli* continues in the presence of iron starvation, although this results in a largely inactive population of these enzymes (Cotruvo and Stubbe, 2011).

Grāve et al. (2020) demonstrated that the enzyme intrinsically selects for manganese in both binding sites when metal-free class Ib RNR from *B. anthracis* is presented with equimolar concentrations of Mn^2+^ and Fe^2+^, whereas no intrinsic selectivity was observed in the case of class Ib RNRs from *S. sanguinis* (Jayachandran et al., 2021). Since the active site consists of two metal binding sites, the question arises to what extent the individual binding sites cooperate to accommodate the cognate metals. The idea that cooperativity helps in the proper metallation of di-metal clusters has been proposed for MntR and R2lox, but so far it does not appear that Ia and Ib RNRs would take advantage of cooperativity, as both metal-binding sites seem to be preorganized without metals (Bollinger et al., 1997; Griese et al., 2013; McGuire et al., 2013; Grāve et al., 2020). Another difference from SODs is that the binding of metals to RNRs appears to be reversible (Cotruvo and Stubbe, 2012). The exchange of metals in binding sites is abolished once the cluster is oxidized. Therefore, it can be speculated that even if a large fraction of class Ia RNRs is mismetallated by manganese, only the correctly metallated cluster with iron ions is oxidized by O_2_ and used for repetitive catalysis. Therefore, even under unfavorable cytosolic conditions, the equilibrium would shift toward the correctly metallated cluster.

##### 4.1.2.3 Novel classes Ic, Id, and Ie of RNRs in bacterial pathogens

In *C. trachomatis*, the critical tyrosine is replaced by phenylalanine (class Ic RNRs) and therefore no radical can be formed at this residue. Instead, Mn^4+^ and Fe^3+^ constitute an oxidized form of a hetero-di-metal cluster in class Ic RNRs, which has one electron less compared to oxidized di-metal clusters in class Ia and Ib RNRs. Overall, the radical is formally located directly on the hetero-di-metal cluster. Thermodynamic calculations indicate that Mn^4+^/Fe^3+^ represents the best configuration in terms of redox potential and relative stability of the intermediates compared to Mn^4+^/Mn^3+^ and Fe^4+^/Fe^3+^ clusters; accordingly, the highest catalytic activity is achieved when manganese and iron are stoichiometrically bound. The hetero-di-metal cluster can be directly activated by O_2_ *in vitro* (Hogbom et al., 2004; Jiang et al., 2007; Roos and Siegbahn, 2009; Roos and Siegbahn, 2011).

Although considerable effort has been made to demonstrate the advantage of placing the radical on the hetero-di-metal cluster when the critical tyrosine is absent (Roos and Siegbahn, 2009; Roos and Siegbahn, 2011), another subclass (Id) has recently emerged, which has been found to generate a radical directly at its di-manganese cluster, yielding Mn^4+^/Mn^3+^ oxidation states. The conserved tyrosine residue is present in the class Id RNR, but its hydroxyl group is oriented away from the di-manganese cluster. Similar to class Ib RNRs, the di-manganese cluster in class Id RNRs is oxidized by ${\text{O}}_{2}^{\mathrm{•-}}$ , but not by O_2_. Surprisingly, no accompanying ortholog to NrdI is encoded on genomes along with genes encoding class Id RNRs (Rose et al., 2018). It has been suggested that class Id RNRs are activated by scavenging ${\text{O}}_{2}^{\mathrm{•-}}$ from the cytosol (Rose et al., 2018). However, this raises a number of uncertainties about how these enzymes compete with highly potent SODs or whether such a strategy is reliable enough *in vivo* given the importance of RNR function. Class Id RNR is also present in an opportunistic pathogen, *Actinobacillus ureae* (Rose et al., 2019).

RNRs in *Mycoplasma* pathogens and one of the two RNRs in *S. pyogenes* belong to class Ie RNRs that lack three otherwise conserved metal-coordinating residues, and it has been proposed that these enzymes have adopted metal-free catalysis. The critical tyrosine residue is conserved within the class Ie RNR, however an unusual derivative of this residue, dihydroxyphenylalanine, was found to carry the radical. Class Ie RNRs are closely related to class Ib RNRs and accordingly require NrdI for activation (Blaesi et al., 2018; Srinivas et al., 2018).

### 4.2 Substitution of iron by manganese in redox-inactive enzymes: alternate metallation

As discussed in Section 4, only redox-inactive mono- and dinuclear iron enzymes can benefit from alternate metallation. It should be noted that the effect of alternate metallation has only been studied in *E. coli* by Imlay’s group. *E. coli* is an iron-centric bacterium, and therefore manganese homeostasis is subordinate to regulation by iron (as briefly discussed in Section 3). Consequently, the effects of alternate metallation with manganese may be more pronounced in iron-centric *E. coli* than in typical Gram-positive bacteria, which may already have manganese as a primary metal in some of the redox-inactive mononuclear enzymes discussed below.

In aerobically cultured wild-type bacteria, quite high ${\text{O}}_{2}^{\mathrm{•-}}$ and/or H_2_O_2_ concentrations must be achieved to overwhelm the well-equipped stress defenses, and this can unfortunately lead to collateral damage caused by formed HO^•^ radicals that ultimately mask the primary ${\text{O}}_{2}^{\mathrm{•-}}$ and/or H_2_O_2_ targets. In *E. coli*, all genes responsible for detoxification of ${\text{O}}_{2}^{\mathrm{•-}}$ and H_2_O_2_ have been identified and well characterized (Seaver and Imlay, 2001), which, after their deletion, allowed careful analysis of the primary targets of mild endogenously generated oxidative stress. This approach revealed the physiological consequence of alternate metallation, as the addition of manganese to stressed cells provided protection for some sensitive mononuclear redox-inactive enzymes.

The first experiments performed with an *E. coli* mutant lacking both *sod* genes showed that this strain, when grown aerobically in defined media, has difficulty processing substrates of the tricarboxylic acid (TCA) cycle, exhibits auxotrophy for branched-chain (BCAA) and aromatic amino acids, and some milder defects in the synthesis of other amino acids. Aconitase and fumarase (both involved in the TCA cycle) and dihydroxyacid dehydratase (BCAA synthesis) utilize an undercoordinated iron atom within the [4Fe-4S] cluster as a Lewis acid. This iron atom can be easily oxidized by ROS, resulting in a decrease in enzyme activity (Carlioz and Touati, 1986; Kuo et al., 1987; Gardner and Fridovich, 1991; Flint et al., 1993). In response, bacteria can switch to a different isoenzyme that is [4Fe-4S]-free, while inducing a more robust Suf (compared to Isc) iron-sulphur assembly and repair machinery (Imlay, 2006; Jang and Imlay, 2010). Some early evidence from *in vitro* experiments suggests that mononuclear enzymes with an undercoordinated iron atom suffer from the same vulnerability (Porter and Austin, 1993). However, the biological consequences of this phenomenon have only recently been studied in detail both *in vitro* and *in vivo*. The conclusions are that the undercoordinated iron atom in mononuclear enzymes is indeed susceptible to oxidative damage. However, unlike [4Fe-4S] proteins, it is much easier to deal with this susceptibility because the iron can be easily replaced by manganese, provided that these enzymes are redox-inactive.

**Figure 10:**
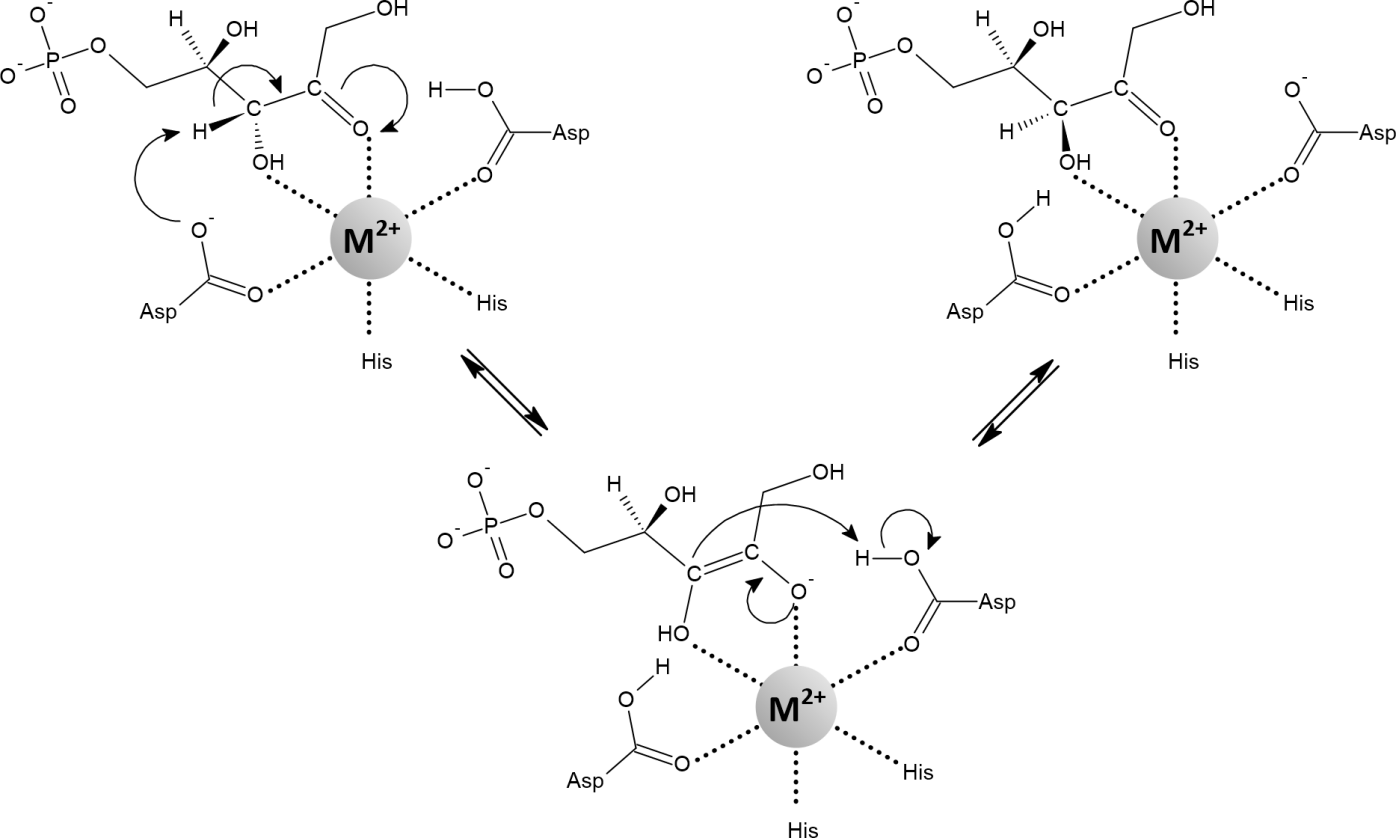
Mechanism of the reaction catalyzed by d-ribulose-5-phosphate 3-epimerase containing an octahedrally coordinated metal (Fe^2+^, Mn^2+^, Co^2+^). The negatively charged *cis*-enediolate intermediate is stabilized by the metal ion in a bidentate manner. Depending on the organism and assay, various metals can activate the enzyme, including Zn^2+^. However, when Zn^2+^ is used to metallate the enzyme, the active site tends to adopt a tetrahedral geometry. In such a case, it has been proposed that the substrate binds as a fifth ligand in a monodentate manner, transiently forming a five-coordinate geometry (Jelakovic et al., 2003; Akana et al., 2006; Caruthers et al., 2006; Liang et al., 2011; Sobota and Imlay, 2011).

Important mononuclear iron enzymes prone to oxidative stress were discovered in an attempt to identify the primary target proteins of H_2_O_2_ toxicity. To this end, an *E. coli* strain was constructed that lacked the H_2_O_2_ scavengers alkyl hydroperoxidase and both catalases (*i.e*., a scavengerless strain) (Seaver and Imlay, 2001). It was confirmed that the [4Fe-4S] enzymes that are sensitive to ${\text{O}}_{2}^{\mathrm{•-}}$ in the *sod*-deficient mutant are also sensitive in the scavengerless mutant (Jang and Imlay, 2007). However, Sobota and Imlay (2011) later found that scavengerless *E. coli* can only grow anaerobically but not aerobically on gluconate if it is also deprived of 6-phosphogluconate dehydratase (encoded by the *edd* gene). Deletion of *edd* gene disables the Entner-Doudoroff pathway, leaving only the pentose-phosphate pathway (PPP) available for gluconate degradation. The PPP can be divided into an oxidative (upper) and a non-oxidative (lower) part. The enzyme that is sensitive to oxidative inactivation is d-ribulose-5-phosphate 3-epimerase (RPE), which operates in the non-oxidative PPP. RPE is a redox-inactive mononuclear enzyme that converts d-ribulose-5-phosphate and d-xylulose-5-phosphate by reversing the chirality of the C-3 carbon on a pentose backbone (**Figure 10**). Human RPE has been shown to utilize iron (Liang et al., 2011), otherwise zinc has been reported as a cognate metal (Jelakovic et al., 2003; Akana et al., 2006; Caruthers et al., 2006). Sobota and Imlay (2011) demonstrated that in *E. coli* iron confers significantly higher catalytic efficiency to the RPE than zinc and that it is the cognate metal *in vivo*. On the other hand, iron in the active site is the cause of RPE susceptibility to oxidative stress. When RPE was loaded with manganese, both *in vitro* and *in vivo* experiments showed that RPE becomes resistant to oxidative damage and supports the growth of the mutant (scavengerless cells lacking *edd* gene) on gluconate even aerobically. Later, it was shown that iron-loaded RPE is also susceptible to ${\text{O}}_{2}^{\mathrm{•-}}$ (Gu and Imlay, 2013). The main function of oxidative PPP is to generate NADPH. During oxidative stress, the demand for NADPH increases rapidly and cells divert carbon flux to the PPP (Christodoulou et al., 2018). The role of the non-oxidative PPP is then to process the sudden rush of d-ribulose-5-phosphate, with the potential option of recycling some of the already consumed carbon for glucose-6-phosphate synthesis, which in turn can enter the oxidative PPP to generate more NADPH (Kuehne et al., 2015). The non-oxidative PPP is also important for redistributing carbon for many cellular purposes by providing d-ribose-5-phosphate and d-erythrose-4-phosphate (Stincone et al., 2015). While oxidative PPP is not ubiquitous and is also absent in several pathogens, non-oxidative PPP is essential (Aono et al., 2015; Richardson et al., 2015; Stincone et al., 2015; Häuslein et al., 2016; Rytter et al., 2021). Therefore, it is reasonable to speculate that the RPE binds manganese rather than iron *in vivo* to keep the non-oxidative PPP functional under oxidative stress.

**Figure 11:**
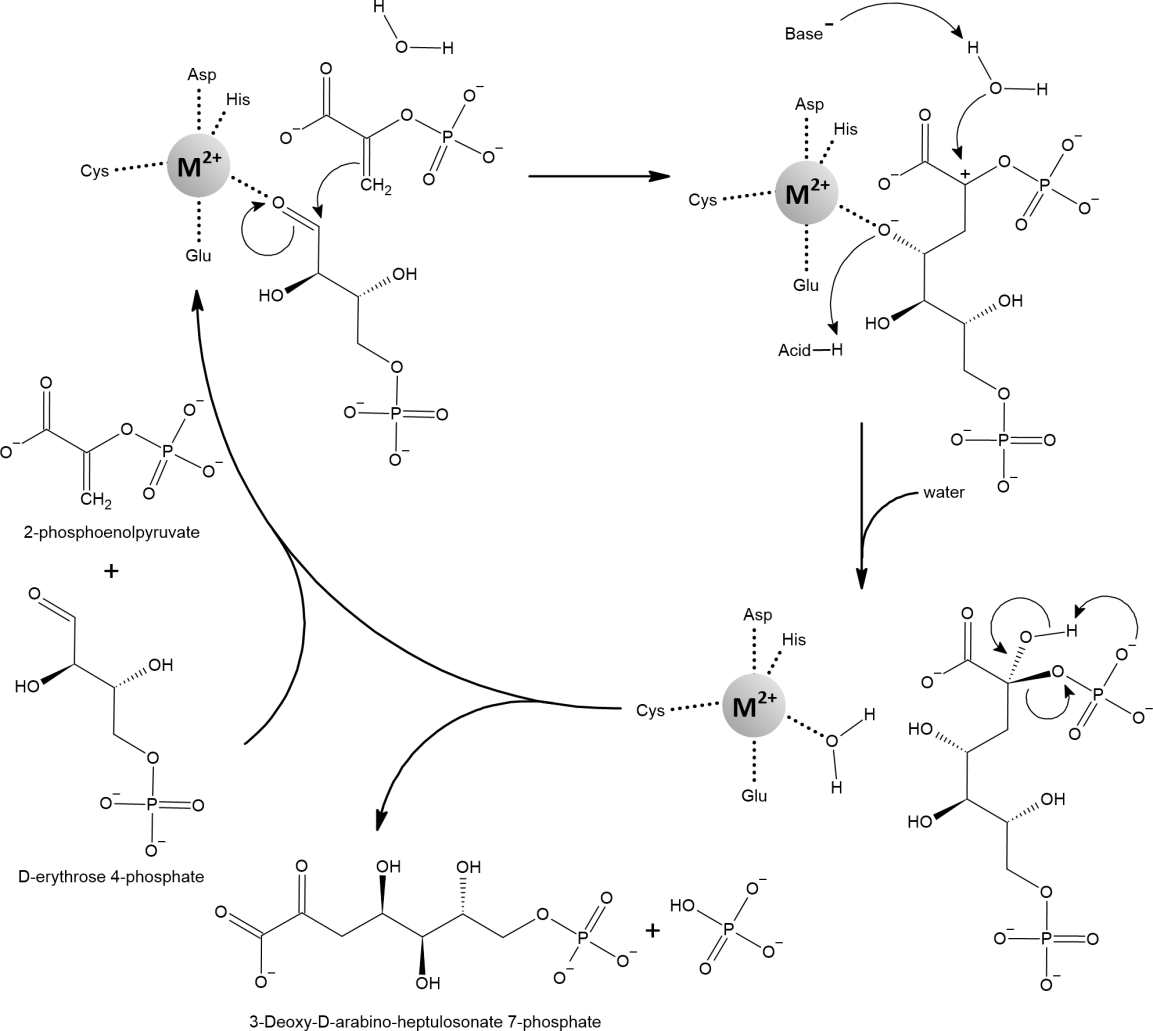
3-deoxy-d-arabinoheptulosonate-7-phosphate synthase (DAHP) catalyzes the aldol-like condensation reaction of 2-phosphoenolpyruvate (PEP) and d-erythrose-4-phosphate (E4P). It has been proposed that the metal ion acts as an electrophile that, by coordinating the oxygen of the aldehyde group of E4P, activates the aldehyde carbon for nucleophilic attack by the enol group of PEP. In the next step, the water molecule attacks the partially positively charged C2 of the former PEP. This is likely facilitated by proton abstraction from the active water molecule by nearby aspartate or glutamate and by concomitant protonation of the metal-coordinated oxygen. Finally, the released product reorganizes to cleave off the phosphate group and to form the ketone group instead. A variety of metals can activate DAHP synthases, including Fe^2+^ and Mn^2+^. Note that the metal-coordinating glutamate can sometimes bind the metal in bidentate mode, changing the overall geometry to an octahedral shape (Shumilin et al., 1999; Furdui et al., 2004; König et al., 2004; Shumilin et al., 2004).

Soon after RPE, other redox-inactive mononuclear iron enzymes were described to benefit from alternate metallation. The cause of the previously observed auxotrophy of aromatic amino acids was attributed to the first enzyme of the shikimate pathway, 3-deoxy-d-arabinoheptulosonate-7-phosphate synthase (DAHP synthase, **Figure 11**). In both *sod*-deficient and scavengerless mutants growing aerobically in a defined glucose medium, the shikimate pathway is defective, but its functionality can be restored by addition of manganese (Sobota et al., 2014). *E. coli* encodes three redox-inactive mononuclear isoenzymes of DAHP synthase that differ in their sensitivity to feedback inhibition by various aromatic amino acids. In the absence of aromatic amino acids, the l-phenylalanine-inhibitable (AroG) isoenzyme is responsible for approximately 80% of DAHP synthase activity (Tribe et al., 1976). Purified AroG showed the highest activity with iron but considerable sensitivity to ${\text{O}}_{2}^{\mathrm{•-}}$ and H_2_O_2_. However, 70% of the activity was retained when AroG was metallated with manganese, while it showed complete resistance to oxidative stress (Sobota et al., 2014). Interestingly, *N. meningitidis* expresses only one DHAP synthase, which reaches the highest activity *in vitro* with manganese (Cross et al., 2013). Significant attenuation of virulence has been reported when *P. aeruginosa* or *B. bronchiseptica* were deprived of DAHP synthase activity (Priebe et al., 2002; McArthur et al., 2003). With respect to pathogens, DAHP synthase is particularly important because it initiates the shikimate pathway that leads to chorismate, which can be used for siderophore synthesis (Dosselaere and Vanderleyden, 2001).

**Figure 12:**
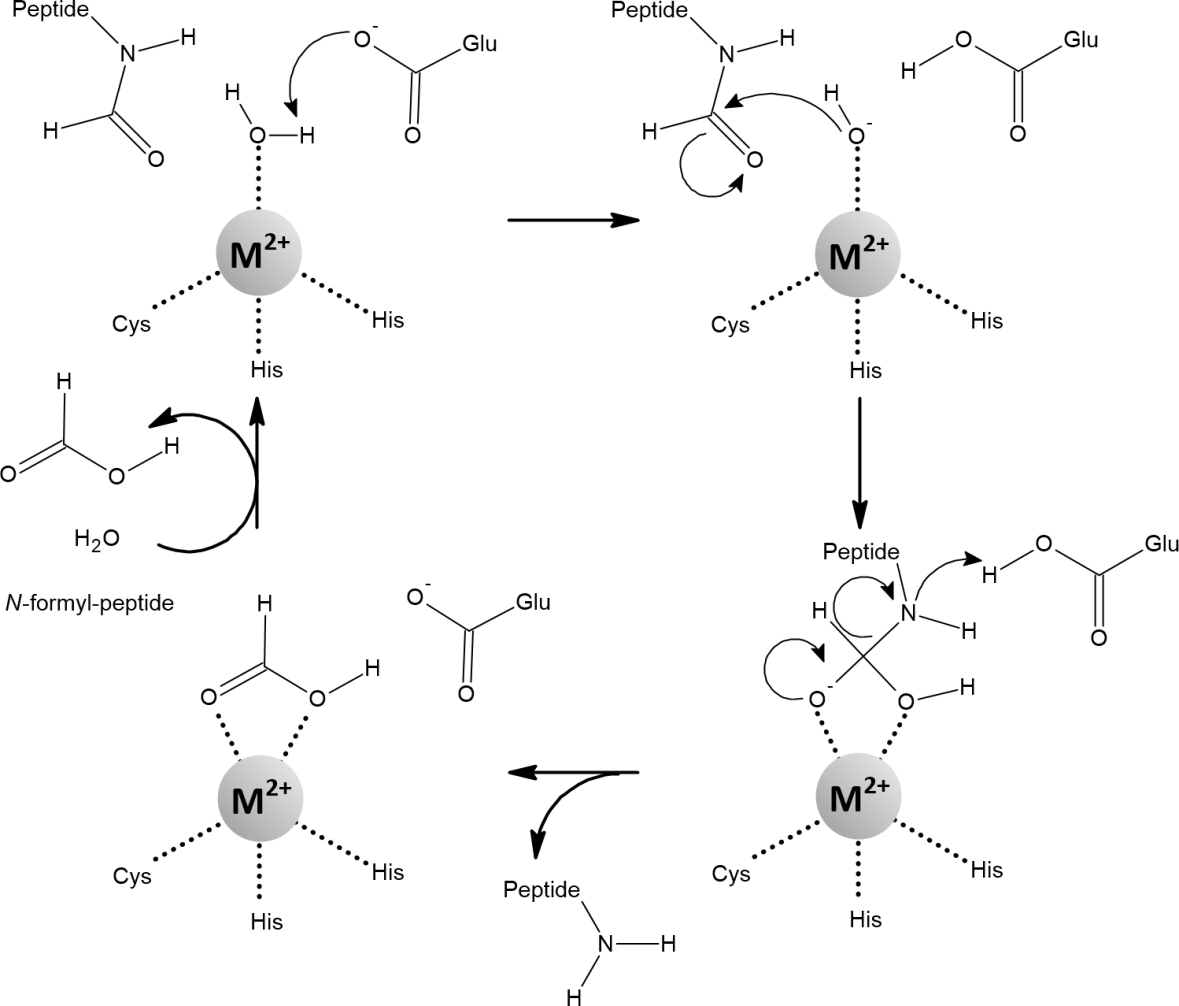
The mechanism of the reaction catalyzed by peptide deformylase (PDF). Coordination of the water molecule by the metal ion allows its deprotonation to form hydroxide. The carbon atom of the *N*-formyl group is subsequently nucleophilically attacked by hydroxide coordinated with the metal. The resulting intermediate is stabilized by a bidentate interaction with the metal ion, which changes its coordination geometry from tetrahedral to five-coordinate. This transition is strongly inhibited when Zn^2+^ is bound in the active site. Fe^2+^, Mn^2+^, and Ni^2+^ are amenable to a flexible change in their coordination geometry, which is the normal function of the PDF (Becker et al., 1998; Groche et al., 1998; Wu et al., 2007; Anjem and Imlay, 2012; Fell et al., 2015).

Peptide deformylase (PDF) is a redox-inactive mononuclear enzyme that was originally thought to be zinc-dependent (**Figure 12**). Confusingly, although it is stable with zinc, its catalytic efficiency is low (Meinnel and Blanquet, 1993; Chan et al., 1997). This was explained by the finding that iron, not zinc, is the native metal. Importantly, the catalytic efficiency of PDF increased at least 100-fold when iron or manganese was added compared to zinc (Rajagopalan et al., 1997; Ragusa et al., 1998; Anjem and Imlay, 2012). Iron in PDF has been shown to be responsible for its sensitivity to H_2_O_2_ and ${\text{O}}_{2}^{\mathrm{•-}}$ , while replacement with manganese protected PDF (Anjem and Imlay, 2012; Gu and Imlay, 2013).

**Figure 13:**
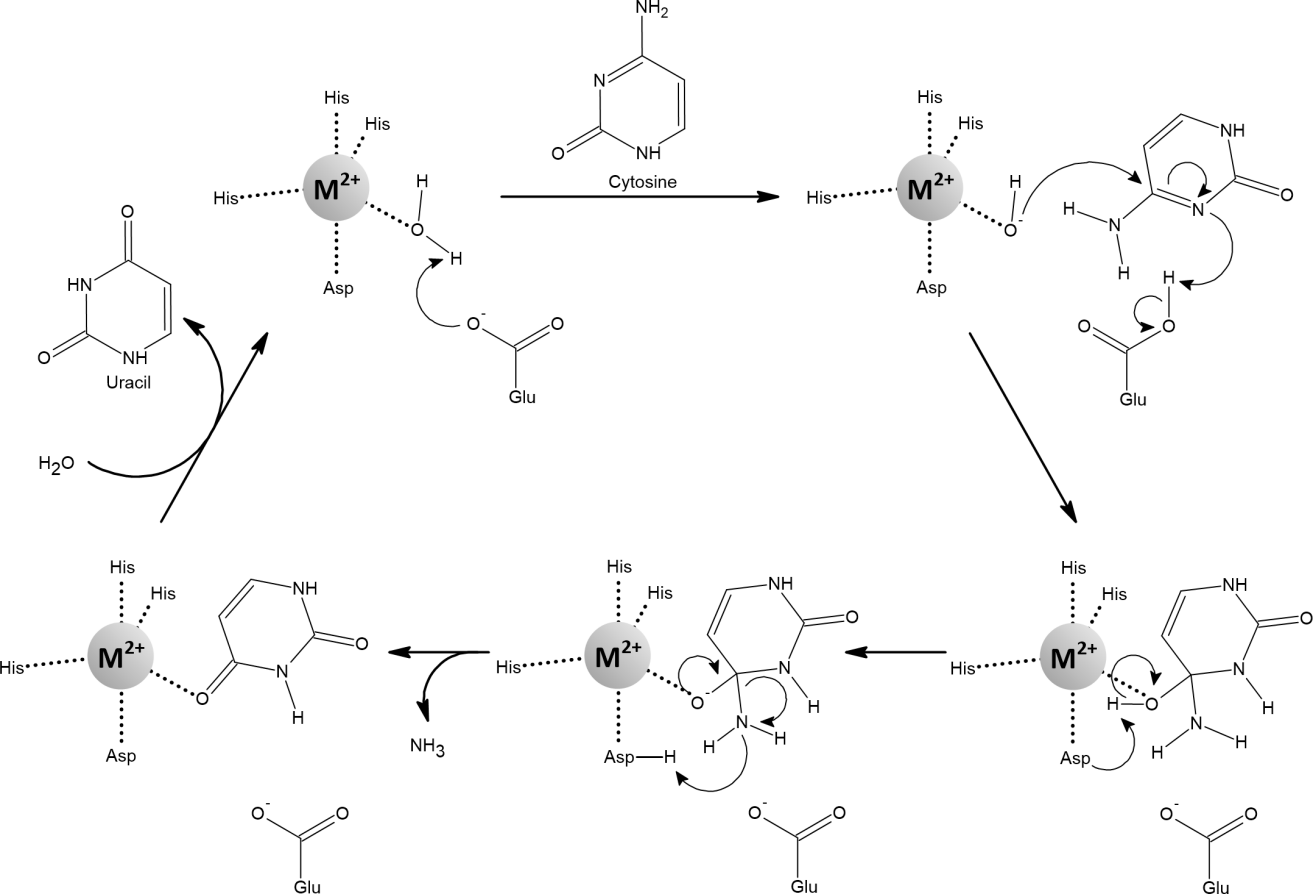
The conversion of cytosine to uracil catalyzed by bacterial cytosine deaminase. The metal ion accommodates a trigonal-bipyramidal geometry with an exchangeable ligand that is a water molecule. A proton is removed from this coordinated water molecule and in the next step the metal-coordinated hydroxide nucleophilically attacks the substrate, simultaneously transferring a proton to the substrate. In the next step, the second proton is transferred from the original water molecule (now hydroxyl group) to the amine group, producing ammonia that leaves the substrate. This step is probably facilitated by the metal-coordinating aspartate. The new water molecule displaces the uracil. Various metals can facilitate this reaction, but iron is most effective (Porter and Austin, 1993; Ireton et al., 2002b; Hall et al., 2011; Anjem and Imlay, 2012; Manta et al., 2014).

**Figure 14:**
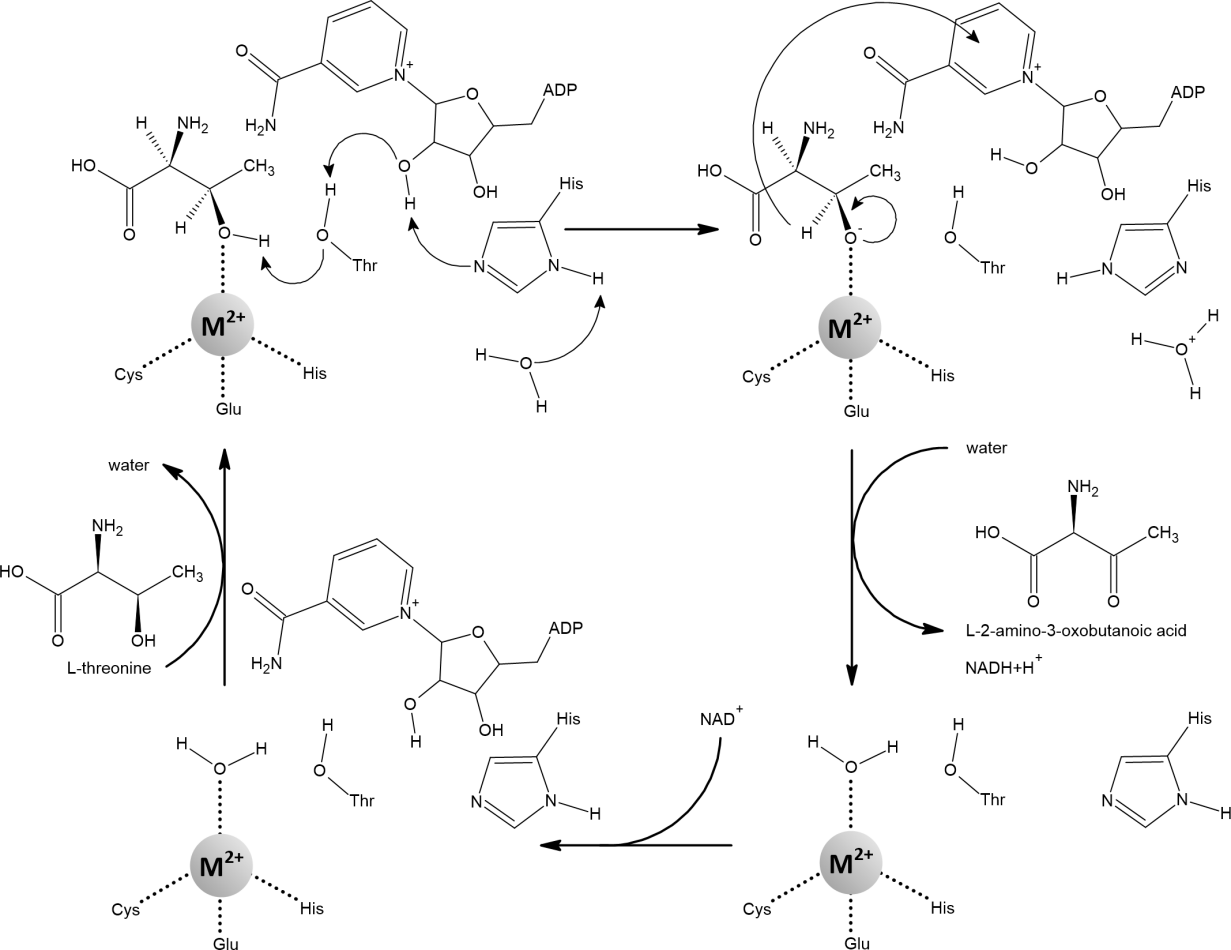
Catalytic mechanism of medium-chain l-threonine dehydrogenase. Only after the binding of NAD+ can l-threonine be accommodated in the active site. During this process, a water molecule is displaced from the coordination shell of the catalytic metal ion. The 3-hydroxyl group of l-threonine is activated by coordination with the metal ion, resulting in the loss of a proton that is released to the external solution. The resulting oxyanion, stabilized by the metal ion, forms a double bond with the third carbon of the substrate and simultaneously transfers a hydride to NAD+. To date, zinc appears to be the most suitable and frequent metal in medium-chain l-threonine dehydrogenases, but there is growing evidence that other metals can also serve this function. When iron is bound, the enzyme becomes sensitive to oxidative stress (Epperly and Dekker, 1991; Machielsen and van der Oost, 2006; Higashi et al., 2008; Bashir et al., 2009; Plapp, 2010; Anjem and Imlay, 2012).

Cytosine deaminase (CD) is found in fungi and bacteria, where it catalyzes the deamination of cytosine to uracil as part of the pyrimidine salvage pathway (**Figure 13**). Fungal and bacterial CDs evolved separately through convergent evolution. While yeast CD is a zinc-dependent enzyme, *E. coli* CD reaches full activity with iron and its activity with zinc is only 15% (Porter and Austin, 1993; Ireton et al., 2002a; Ireton et al., 2003). CD isolated from *E. coli* contains mainly iron and is therefore readily inactivated under aerated conditions and by H_2_O_2_ treatment. However, a substantial portion of the activity is retained when iron is replaced with manganese, and complete protection against oxidative stress is achieved (Porter and Austin, 1993; Anjem and Imlay, 2012).

l-threonine dehydrogenases (TDHs) initiate a two-step catabolic degradation of l-threonine to acetyl-coenzyme A and glycine (**Figure 14**). TDHs are members of the alcohol dehydrogenase superfamily and within this superfamily belong to either the short-chain or medium-chain family (Persson et al., 2008; Adjogatse et al., 2018). TDHs of the medium-chain type have two metal-binding sites, a zinc-dependent structural site and a catalytic site (Auld and Bergman, 2008). Which is the cognate metal for the catalytic site in these TDHs is controversial. Usually, there is another zinc atom in the active site that gives the enzyme the highest activity. However, some publications have reported that other metals can substitute for zinc, with varying degrees of retention of enzymatic activity (Epperly and Dekker, 1991; Johnson et al., 1998; Machielsen and van der Oost, 2006; Higashi et al., 2008; Bashir et al., 2009). Surprisingly, iron has not been thoroughly considered until recently. Anjem and Imlay, (2012) showed that TDH in *E. coli* binds iron *in vitro* and *in vivo*, which in turn led to rapid inactivation of TDH by H_2_O_2_ treatment. Alternate metallation by various metals, including manganese, made TDH significantly more resistant to oxidative damage (Anjem and Imlay, 2012).

As can be seen from the previous information, it is sometimes difficult to determine the native metal in mononuclear enzymes. Iron is unstable during protein purification under aerobic conditions and can be replaced by the more competitive zinc (Irving and Williams, 1948), giving the impression that zinc is the cognate metal. In *E. coli*, the surrogate host of choice for recombinant expression, manganese, unlike iron and zinc, is not imported under standard growth conditions because *mntH* is under the control of Fur and OxyR (Section 3). Altogether, it could be suggested that the extent of alternate metallation by manganese instead of iron has been underestimated so far. Regardless, based solely on the examples published by Imlay’s group, it is clear that manganese is of paramount importance when bacteria are exposed to oxidative stress because it mitigates the pathological effects of iron, a burden passed on by their microbial predecessors.

## 5 Struggle for manganese between human host and pathogens

### 5.1 Host mechanisms leading to manganese deficiency in pathogens

In 1975, Weinberg introduced the term “nutritional immunity”. At that time, nutritional immunity referred to the host’s ability to withhold iron from microbial invaders (Weinberg, 1975). However, it was recognized that other transition metals were also subject to the host-pathogen tug-of-war, particularly copper, zinc, and manganese. In addition, it became clear that elevated concentrations of some transition metals can be used as part of innate immunity for the opposite strategy to intoxicate pathogens.

Ceruloplasmin is the major copper-carrying protein in the blood and also plays a role in iron metabolism. Copper bound to ceruloplasmin catalyzes the oxidation of Fe^2+^ to Fe^3+^, thereby supporting iron loading on transferrin (Roeser et al., 1970). Serum ceruloplasmin levels increase during infection, possibly due to its antioxidant activity and higher consumption of copper by host phagocytic cells, where copper is used for intracellular intoxication of pathogens (Natesha et al., 1992; White et al., 2009; Besold et al., 2016; Ladomersky et al., 2017). Overall, serum copper levels increase during infection, while iron and zinc levels decrease. Chronic infection can even lead to hypoferremia and hypozincemia (Letendre and Holbein, 1984; Alker and Haase, 2018). Zinc is stable and competitive enough to establish serum albumin as its primary carrier (Handing et al., 2016). There is evidence that zinc can also be used for intracellular intoxication of pathogens (Botella et al., 2011). Manganese presents a conundrum: Mn^2+^ is soluble and stable under aerobic conditions, unlike the unstable Fe^2+^. The problem is that Mn^2+^ is not competitive enough (Irving and Williams, 1948) and binds only loosely to some transport proteins such as transferrin, albumin, and α_2_-macroglobulin or to low molecular weight chelates (Rabin et al., 1993; Harris and Chen, 1994; Fanali et al., 2012). It has been proposed that several human transporters of divalent metals import Mn^2+^ in addition to their primary substrate, suggesting the presence of free or loosely bound Mn^2+^ in serum (Gunter et al., 2013). Mn^3+^ is much more competitive than Mn^2+^, but Mn^3+^ is unstable with respect to redox reactions and therefore usually occurs tightly bound to transferrin in serum (Jursa and Smith, 2009). Moreover, Mn^3+^ can be stabilized in solution by various ligands such as citrate (Trouwborst et al., 2006; Topolski, 2011; Gunter et al., 2013). On the other hand, due to the high redox potential of manganese, it is much more difficult to oxidize Mn^2+^ compared to Fe^2+^. It has been suggested that ceruloplasmin may aid in the oxidation of Mn^2+^, which would facilitate the loading of Mn^3+^ onto transferrin similar to Fe^2+^ (Davidsson et al., 1989). However, this idea was refuted in the study by Jursa and Smith (2009), in which ceruloplasmin deficient mice exhibited the same amount of manganese bound to transferrin as wild type mice.

S100 proteins are low-molecular-weight, calcium-binding proteins unique to vertebrates and, although structurally similar, differ significantly in function. Calprotectin (CP) is a heteroligomer of two S100 proteins (S100A8 and S100A9) associated with inflammation in human tissues. CP is produced in some types of myeloid cells, but most notably it accounts for approximately 5% of the total protein in neutrophils. In addition, expression of CP can be induced in epithelial cells and keratinocytes (Fagerhol et al., 1980; Dale et al., 1983; Singh and Ali, 2022). In the cytosol, CP forms a heterodimer (S100A8/S100A9), while release into the extracellular space allows CP to bind calcium, promoting the formation of a heterotetramer (S100A8/S100A9)_2_. The heterodimer binds the first-row transition metals relatively weakly compared to the calcium-bound heterotetramer, which has been reported to have markedly increased affinity for Mn^2+^, Fe^2+^, Ni^2+^, and Zn^2+^ (Zygiel and Nolan, 2018). Originally, it was thought that only the retention of Zn^2+^ by CP in the presence of calcium was responsible for its protective function (Clohessy and Golden, 1995). However, it was later clearly demonstrated in the seminal work of Corbin et al. (2008) that both Zn^2+^ and Mn^2+^ are removed by CP from tissue abscesses caused by *S. aureus* infection. Finally, these studies have recently been refined by Nolan’s group, which found that Fe^2+^ and Ni^2+^ are also tightly sequestered by CP in the presence of calcium (Nakashige et al., 2015; Nakashige et al., 2017). CP binds first-row divalent transition metals at two sites located at the interface between S100A8 and S100A9. Site 1 can coordinate the bound metal in a tetrahedral or trigonal bipyramidal geometry through three histidine and one aspartate residues (aspartate coordinates in a monodentate or bidentate fashion), while site 2 coordinates the bound metal in a perfect octahedral geometry through six histidine residues. In a calcium-dependent manner, only octahedral site 2, which consists of six histidine residues, exhibits high affinity for Mn^2+^, Fe^2+^, and Ni^2+^, while Zn^2+^ can be tightly bound to both sites (Zygiel and Nolan, 2018). The affinities of site 2 for Mn^2+^, Fe^2+^, Ni^2+^, and Zn^2+^ correlate with the Irving-Williams series (Nakashige et al., 2017; Zygiel and Nolan, 2018). In the presence of calcium, site 2 exhibits very high affinity even for less competitive metals such as Mn^2+^ and Fe^2+^ in the nanomolar and picomolar range, respectively. As an explanation, it has been proposed that the flexible C-terminal tail of the S100A9 subunit, which contains two of the six histidine residues required for the assembly of site 2, encapsulates the transition metal during the binding process and thus effectively prevents that the metal escapes (Brophy et al., 2013; Nakashige et al., 2015). Of note, when studying the response of *Candida albicans* to CP, Besold and colleagues observed that CP can also sequester Cu^2+^, which is particularly useful against eukaryotic pathogens that have higher copper requirements compared to prokaryotes (Besold et al., 2018).

Emil Skamene and his colleagues recognized that the phenotype responsible for resistance or susceptibility to some intracellular pathogens, including mycobacterial infections, is controlled by a single dominant autosomal gene. This gene has been shown to be expressed in phagocytic cells, where the protein is recruited to the membrane of the microbe-containing phagosome during the maturation process. The gene was named Natural resistance-associated macrophage protein 1 (Nramp1), and the Nramp1 protein was later demonstrated to be a transporter for divalent metals. Biologically relevant substrates of Nramp1 are Fe^2+^ and Mn^2+^, which are pumped into the cytosol of the phagocytic cell, thereby depriving the phagolysosomal interior of these metals (Vidal et al., 1993; Skamene, 1994; Supek et al., 1996; Skamene et al., 1998; Forbes and Gros, 2001; Forbes and Gros, 2003; Cellier et al., 2007). Homology searches revealed that the Nramp1 protein is not only ubiquitous in eukaryotes but, surprisingly, also highly abundant in bacteria, albeit under a different name: MntH (Cellier et al., 2001). Overall, Nramp1-mediated depletion of Fe^2+^ and Mn^2+^ is a crucial process without which phagosomes can never fully mature and exert their bactericidal effects (Hackam et al., 1998).

Overall, CP sequesters the first-row transition metals in their reduced, divalent state in the extracellular environment at the site of infection. Excessive amounts of released CP (> 1µM) may provide the capacity to sequester more competitive Zn^2+^ and perhaps Fe^2+^ and Ni^2+^ in addition to less competitive Mn^2+^ (Johne et al., 1997). Because Mn^2+^ is difficult to oxidize, the action of CP could be sufficient even during the oxidative burst in an inflamed tissue, and perhaps when some of the manganese is oxidized, transferrin could eventually remove Mn^3+^. When pathogens are trapped in phagolysosomes, Nramp1 removes Mn^2+^ and Fe^2+^ from this compartment. Nevertheless, whereas these scenarios involve a localized immune response, it is unclear how manganese is managed at the systemic level to refrain from pathogen colonization and avoid inflammation-related damage.

### 5.2 Adaptations of pathogens that have evolved to counteract manganese deficiency in their hosts

Manganese is essential for some processes (*e.g*., sporulation) or enzymatic activities, but another conditional role of manganese is to replace iron where possible during oxidative stress or iron starvation. Therefore, host oxidative burst and sequestration of iron would be ineffective if pathogens had access to manganese, as shown by the activities of Nramp1 and CP in the context of nutritional immunity. It is already known that manganese importers are critical for virulence. In bacterial pathogens, the aforementioned MntH, a high capacity, low affinity proton symporter, can be complemented by a low capacity, high affinity ATP-binding cassette transporter (MntABC). Both MntH and MntABC can also be sole manganese importers in individual species. The topic of manganese importers has been discussed in detail elsewhere (Juttukonda and Skaar, 2015; Kelliher and Kehl-Fie, 2016; Neville et al., 2021) and will not be discussed further here. In this review, we would like to highlight two less explored, but all the more intriguing, adaptations that may help pathogens overcome manganese scarcity in the host.

Siderophores have traditionally been associated exclusively with chelation and uptake of iron from the environment and from host iron-sequestering proteins (Golonka et al., 2019). Other metals, long overshadowed by iron, have not been thoroughly considered. The possibility that siderophores could bind manganese seemed unlikely because Mn^2+^ does not readily oxidize (**Figure 2**) and Mn^3+^ was considered an irrelevant chemical species in the environment because of its tendency to disproportionate or act as a strong oxidant. However, Faulkner et al. (1994) analyzed a green complex formed when desferrioxamine B (DFO-B, normally considered an iron siderophore) and Mn^2+^ are mixed in the presence of O_2_ and found that it contains Mn^3+^. This result was confirmed by Duckworth and Sposito (2005) and extended by determining the stability constant, which was remarkably high at log*K* 29.9, approaching the stability constant of the DFO-B complex with iron at log*K* 32.02 (Kraemer, 2004). Harrington et al. (2012) compared the stability constants of various siderophores to iron or manganese and found that two pyoverdins (one produced by *P. aeruginosa* and one by *P. putida*) and rhizoferrin (produced by the fungus *Rhizopus arrhizus* and by the bacterium *Ralstonia picketti*) had significantly higher affinities for Mn^3+^ than for Fe^3+^. Only slightly lower (one to two orders of magnitude) stability constants with manganese compared to iron were found for desferricrocin and triacetylfusarinin C produced by the fungus *Aspergillus fumigatus* (Farkas et al., 2014). The oxidation of Mn^2+^ after complexation by siderophores (DFO-B, DFO-E, and putrebactin) in the presence of O_2_ was studied using the Evans NMR method (Springer and Butler, 2015). Finally, Mn^3+^ was shown to be an abundant ion in suboxic zones, where it is presumably stabilized by as yet unidentified siderophores (Trouwborst et al., 2006; Madison et al., 2013). These observations clearly show that the redox potential of manganese can be drastically lowered after complexation, so that it is even oxidized by O_2_ and Mn^3+^ is subsequently firmly held by the siderophore. Siderophores are normally expressed when bacteria are iron-starved (Hider and Kong, 2010). Oxidative stress induces sequestration of iron in bacteria (Section 3), and therefore in this respect the defense against oxidative stress is similar to iron deficiency. However, import of additional iron during oxidative stress could further undermine the defense against oxidative stress. As far as we know, acquisition of manganese by imported siderophores has never been associated with virulence. However, two recent reports have shown that yersiniabactin, traditionally considered a siderophore, also binds zinc *in vivo*, helping *Y. pestis* and *E. coli* Nissle 1917 to overcome zinc sequestration by CP. In the case of *Y. pestis*, yersiniabactin contributes to establish the disease, but the same activity of yersiniabactin in probiotic *E. coli* Nissle 1917 serves the bacterium to outcompete pathogens such as *S.* Typhimurium (Behnsen et al., 2021; Price et al., 2021).

The first bacterial exporter of manganese was not discovered until 2009, when Rosch and colleagues identified it in *S. pneumoniae* (Rosch et al., 2009). Since then, additional reports have followed showing that manganese exporters are much more diverse compared to manganese importers (summarized in **Table 1**). Recently, we reported that *B. pertussis* encodes a manganese exporter that atypically belongs to the Ca^2+^:cation exchanger superfamily. Unexpectedly, most sequenced *B. pertussis* strains turned out to have a nonfunctional variant of the gene. Accordingly, reference strain Tohama I accumulated significant amounts of manganese, which contributed to its improved growth *in vitro* in the presence of paraquat, an oxidative stress inducer. Surprisingly, after exposure to moderate concentrations of manganese, which are nevertheless toxic if manganese exporter is not functional, *B. pertussis* was able to reactivate manganese export function and restore growth. Reactivation was achieved by excising an inhibitory duplication in the coding sequence of the exporter. Unfortunately, we could not find conditions under which the duplication was reinserted, thereby deactivating manganese export, an event that occurred during the evolution of this pathogen. Furthermore, we found that in the close relative *B. parapertussis*, the orthologous exporter is inactivated by a frameshift mutation, suggesting convergent evolution of *B. pertussis* and *B. parapertussis* toward higher intracellular manganese supplies (Capek et al., 2021). Prior to us, Veyrier et al. (2011) pointed out that in the majority of *N. gonorrhoeae* strains, the gene encoding the manganese exporter, called *mntX*, contains a frameshift mutation associated with higher sensitivity to manganese. In this context, it has long been known that *N. gonorrhoeae* accumulates manganese, which significantly strengthens its defenses against oxidative stress (Tseng et al., 2001). After deletion of the manganese exporter, increased resistance to oxidative stress was observed *in vitro* in *S. pneumoniae* and *V. cholerae* (Rosch et al., 2009; Fisher et al., 2016) whereas *in vivo*, deletion of the manganese exporter led to a decrease in virulence in *S. aureus*, *S. pneumoniae*, *E. faecalis*, *B. abortus*, *N. meningitidis*, and *M. tuberculosis* (Rosch et al., 2009; Veyrier et al., 2011; Grunenwald et al., 2019; Johnsrude et al., 2019; Lam et al., 2020). Thus, it appears that the majority of pathogens require a functional manganese exporter, but the fundamental reason for this observation is elusive, as we believe manganese overload, as suggested by some, is highly unlikely in the host. Clearly, we are missing an important piece of the puzzle that would provide an explanation for why a few pathogens inactivate their manganese exporters while the majority of others do not. We speculate that this may depend on the infectious environment and the extent of manganese requirement during infection of the particular pathogen.

**Table 1:** Overview and classification of bacterial manganase exporters.

Superfamily	Family	Protein	Bacteria	References
CDF	CDF	MntE^1^	*Streptococcus pneumoniae*	(Rosch et al., 2009)
		MntE	*Streptococcus suis*	(Xu et al., 2017)
		MntE	*Streptococcus pyogenes*	(Turner et al., 2015)
		MntE	*Staphylococcus aureus*	(Grunenwald et al., 2019)
		MntE	*Deinococcus radiodurans*	(Sun et al., 2010)
		MntE	*Enterococcus faecalis*	(Lam et al., 2020)
		MneP^2^	*Bacillus subtilis*	(Huang et al., 2017)
		MneS^2^	*Bacillus subtilis*	(Huang et al., 2017)
		EmfA^3^	*Rhizobium etli*	(Cubillas et al., 2014)
		EmfA^3^	*Brucella abortus*	(Johnsrude et al., 2019)
		YiiP^4^	*Salmonella* Typhimurium	(Ouyang et al., 2022)
		SmYiiP	*Sinorhizobium meliloti*	(Raimunda and Elso-Berberián, 2014)
LysE	LysE (DUF204)	MntP	*Escherichia coli*	(Waters et al., 2011)
		YebN	*Xanthomonas oryzae*	(Li et al., 2011)
		MntX	*Neisseria meningitidis*	(Veyrier et al., 2011)
		MntX^5^	*Neisseria gonorrhoeae*	(Veyrier et al., 2011)
		MntP^4^	*Salmonella* Typhimurium	(Ouyang et al., 2022)
	UPF0016	Mnx	*Synechocystis* sp.	(Brandenburg et al., 2017)
		MneA	*Vibrio cholerae*	(Fisher et al., 2016)
		MneA	*Vibrio fischeri*	(Zeinert et al., 2018)
		MneA	*Streptomyces* sp.	(Zeinert et al., 2018)
	TerC	YceF^2^	*Bacillus subtilis*	(Paruthiyil et al., 2020)
		YkoY^2^	*Bacillus subtilis*	(Paruthiyil et al., 2020)
P-type ATPase	P-type ATPase	CtpC	*Mycobacterium tuberculosis*	(Padilla-Benavides et al., 2013)
	P_II_-type ATPase	MgtA^1^	*Streptococcus pneumoniae*	(Martin et al., 2019)
Ca^2+^:cation exchanger	YRBG	BP3410^6^	*Bordetella pertussis*	(Capek et al., 2021)
		BPP3560^7^	*Bordetella parapertussis*	(Capek et al., 2021)

^1^The export of Mn^2+^ is primarily controlled by MntE. MgtA exports both Ca^2+^ and Mn^2+^ under conditions of extreme Mn^2+^ toxicity induced by a specific genetic background.

^2^MneP and MneS are primary exporters. The effect on suppression of manganese toxicity of YceF and YkoY is detectable only in the Δ*mneP* and Δ*mneS* double deletion mutant. Intriguingly, Alx, a TerC family protein from *E. coli*, does not appear to act as an exporter, although it is under the control of the Mn-sensing riboswitch. Indeed, expression of Alx in the presence of manganese leads to increased levels of intracellular manganese (Zeinert et al., 2018).

^3^EmfA represent a transporter responsible for efflux of Mn^2+^ and Fe^2+^. While *R. etli* EmfA exports both Mn^2+^ and Fe^2+^, *B. abortus* EmfA exports only Mn^2+^.

^4^MntP was induced by iron and manganese, whereas YiiP levels remained unchanged. Deletion of both genes had additive effects when S. Typhimurium was challenged by excessive manganese, suggesting a scalable response.

^5^In the case of *N. gonorrhoeae*, most strains carry a frameshift mutation in the *mntX* gene.

^6^98% of fully sequenced isolates of *B. pertussis* carry an inhibitory duplication that prevents the export activity of the Mn exporter BP3410.

^7^All but one isolate Bpp5 carry a frameshift mutation in the Mn exporter BPP3560.

## 6 Conclusions

Regarding its use in the biosphere, we believe that manganese was strongly displaced by iron due to its favorable low redox potential and much higher concentration in primordial anoxic oceans (more than 2.5 billion years ago). However, with the use of manganese in oxygenic photosynthesis, the opportunities for general use of manganese in the biosphere changed dramatically. Accumulated oxygen leveled the differences in bioavailable concentrations of iron and manganese and made the redox cycling of manganese largely accessible to the biosphere. On the other hand, iron has become a major contributor to oxidative stress, which has put pressure on the development of mechanisms to control its free cytosolic concentration, accompanied by efforts to replace it. By this time, however, the fundamentals of cellular metabolism with irreversibly incorporated iron had been firmly established. Nevertheless, at least some enzymes with relatively simple active sites, mononuclear and dinuclear, were able to adapt to using manganese in place of iron. An iron-dependent enzyme catalyzing a redox reaction can be replaced by a manganese-dependent isoenzyme, provided it has evolved an adaptation that allows it to overcome the higher redox potential of manganese. In the case of redox-inactive enzymes, iron can be replaced directly within the individual enzyme, and the metal used depends largely on the free cytosolic Fe:Mn ratio. Particularly in the case of pathogens, the second mechanism of direct exchange has not yet been considered and may be a promising avenue to explore. It is now clear that bacterial pathogens use manganese as an important element of defense against host innate immunity and that host immune cells have evolved intricate mechanisms to deprive pathogens of manganese. Despite recent advances, manganese homeostasis in both pathogens and their hosts is far from fully understood. In humans, for example, it is largely unknown how manganese is handled at the systemic level in the healthy state or during infection.

## Author contributions

JČ: Writing of the original draft, JČ and BV: Writing of the manuscript, editing and conceptualization. JČ: Preparation of tables and figures. All authors contributed to the article and approved the submitted version.

## References

[B1] Abbouni B. Oehlmann W. Stolle P. Pierik A. J. Auling G. (2009). Electron paramagnetic resonance (EPR) spectroscopy of the stable-free radical in the native metallo-cofactor of the manganese-ribonucleotide reductase (Mn-RNR) of *Corynebacterium glutamicum* . Free Radic. Res. 43 (10), 943–950. doi: 10.1080/10715760903140568 19707921

[B2] Adjogatse E. Erskine P. Wells S. A. Kelly J. M. Wilden J. D. Chan A. W. E.. (2018). Structure and function of L-threonine-3-dehydrogenase from the parasitic protozoan *Trypanosoma brucei* revealed by X-ray crystallography and geometric simulations. Acta Crystallogr. D Struct. Biol. 74 (Pt 9), 861–876. doi: 10.1107/s2059798318009208 30198897

[B3] Afonyushkin T. Vecerek B. Moll I. Blasi U. Kaberdin V. R. (2005). Both RNase E and RNase III control the stability of sodB mRNA upon translational inhibition by the small regulatory RNA RyhB. Nucleic Acids Res. 33 (5), 1678–1689. doi: 10.1093/nar/gki313 15781494 PMC1069011

[B4] Aguirre J. D. Clark H. M. McIlvin M. Vazquez C. Palmere S. L. Grab D. J.. (2013). A manganese-rich environment supports superoxide dismutase activity in a Lyme disease pathogen, *Borrelia burgdorferi* . J. Biol. Chem. 288 (12), 8468–8478. doi: 10.1074/jbc.M112.433540 23376276 PMC3605662

[B5] Aguirre J. D. Culotta V. C. (2012). Battles with iron: manganese in oxidative stress protection. J. Biol. Chem. 287 (17), 13541–13548. doi: 10.1074/jbc.R111.312181 22247543 PMC3340200

[B6] Akana J. Fedorov A. A. Fedorov E. Novak W. R. Babbitt P. C. Almo S. C.. (2006). D-ribulose 5-phosphate 3-epimerase: functional and structural relationships to members of the ribulose-phosphate binding (beta/alpha)8-barrel superfamily. Biochemistry 45 (8), 2493–2503. doi: 10.1021/bi052474m 16489742

[B7] Alker W. Haase H. (2018). Zinc and sepsis. Nutrients 10 (8), 976. doi: 10.3390/nu10080976 30060473 PMC6115943

[B8] Al-Maghrebi M. Fridovich I. Benov L. (2002). Manganese supplementation relieves the phenotypic deficits seen in superoxide-dismutase-null *Escherichia coli* . Arch. Biochem. Biophys. 402 (1), 104–109. doi: 10.1016/S0003-9861(02)00065-6 12051688

[B9] Alqurashi A. Alfs L. Swann J. Butt J. N. Kelly D. J. (2021). The flavodoxin FldA activates the class Ia ribonucleotide reductase of *Campylobacter jejuni* . Mol. Microbiol. 116 (1), 343–358. doi: 10.1111/mmi.14715 33721378

[B10] Altuvia S. Almiron M. Huisman G. Kolter R. Storz G. (1994). The dps promoter is activated by OxyR during growth and by IHF and σ S in stationary phase. Mol. Microbiol. 13 (2), 265–272. doi: 10.1111/j.1365-2958.1994.tb00421.x 7984106

[B11] Anbar A. D. (2008). Oceans. elements and evolution. Science 322 (5907), 1481–1483. doi: 10.1126/science.1163100 19056967

[B12] Andersson C. S. Hogbom M. (2009). A *Mycobacterium tuberculosis* ligand-binding Mn/Fe protein reveals a new cofactor in a remodeled R2-protein scaffold. Proc. Natl. Acad. Sci. U.S.A. 106 (14), 5633–5638. doi: 10.1073/pnas.0812971106 19321420 PMC2667070

[B13] Andreini C. Bertini I. Cavallaro G. Holliday G. L. Thornton J. M. (2008). Metal ions in biological catalysis: from enzyme databases to general principles. JBIC J. Biol. Inorganic Chem. 13 (8), 1205–1218. doi: 10.1007/s00775-008-0404-5 18604568

[B14] Anjem A. Imlay J. A. (2012). Mononuclear iron enzymes are primary targets of hydrogen peroxide stress. J. Biol. Chem. 287 (19), 15544–15556. doi: 10.1074/jbc.M111.330365 22411989 PMC3346116

[B15] Anjem A. Varghese S. Imlay J. A. (2009). Manganese import is a key element of the OxyR response to hydrogen peroxide in *Escherichia coli* . Mol. Microbiol. 72 (4), 844–858. doi: 10.1111/j.1365-2958.2009.06699.x 19400769 PMC2776087

[B16] Aono R. Sato T. Imanaka T. Atomi H. (2015). A pentose bisphosphate pathway for nucleoside degradation in archaea. Nat. Chem. Biol. 11 (5), 355–360. doi: 10.1038/nchembio.1786 25822915

[B17] Archibald F. S. Duong M. N. (1984). Manganese acquisition by *Lactobacillus plantarum* . J. Bacteriol 158 (1), 1–8. doi: 10.1128/jb.158.1.1-8.1984 6715278 PMC215370

[B18] Archibald F. S. Duong M. N. (1986). Superoxide dismutase and oxygen toxicity defenses in the genus *Neisseria* . Infect. Immun. 51 (2), 631–641. doi: 10.1128/iai.51.2.631-641.1986 3943903 PMC262393

[B19] Archibald F. S. Fridovich I. (1981). Manganese, superoxide dismutase, and oxygen tolerance in some lactic acid bacteria. J. Bacteriol 146 (3), 928–936. doi: 10.1128/jb.146.3.928-936.1981 6263860 PMC216946

[B20] Atack J. M. Kelly D. J. (2009). Oxidative stress in *Campylobacter jejuni*: responses, resistance and regulation. Future Microbiol. 4 (6), 677–690. doi: 10.2217/fmb.09.44 19659424

[B21] Atta M. Nordlund P. Aberg A. Eklund H. Fontecave M. (1992). Substitution of manganese for iron in ribonucleotide reductase from *Escherichia coli*. Spectroscopic and crystallographic characterization. J. Biol. Chem. 267 (29), 20682–20688. doi: 10.1016/S0021-9258(19)36739-0 1328209

[B22] Auld D. S. Bergman T. (2008). Medium- and short-chain dehydrogenase/reductase gene and protein families : The role of zinc for alcohol dehydrogenase structure and function. Cell Mol. Life Sci. 65 (24), 3961–3970. doi: 10.1007/s00018-008-8593-1 19011745 PMC11131767

[B23] Babcock G. T. Wikstrom M. (1992). Oxygen activation and the conservation of energy in cell respiration. Nature 356 (6367), 301–309. doi: 10.1038/356301a0 1312679

[B24] Bakshi C. S. Malik M. Regan K. Melendez J. A. Metzger D. W. Pavlov V. M.. (2006). Superoxide dismutase B gene (sodB)-deficient mutants of *Francisella tularensis* demonstrate hypersensitivity to oxidative stress and attenuated virulence. J. Bacteriol 188 (17), 6443–6448. doi: 10.1128/JB.00266-06 16923916 PMC1595384

[B25] Banci L. Bertini I. Calderone V. Cramaro F. Del Conte R. Fantoni A.. (2005). A prokaryotic superoxide dismutase paralog lacking two Cu ligands: from largely unstructured in solution to ordered in the crystal. Proc. Natl. Acad. Sci. U.S.A. 102 (21), 7541–7546. doi: 10.1073/pnas.0502450102 15897454 PMC1140445

[B26] Barber J. (2017). A mechanism for water splitting and oxygen production in photosynthesis. Nat. Plants 3, 17041. doi: 10.1038/nplants.2017.41 28368386

[B27] Barnese K. Gralla E. B. Valentine J. S. Cabelli D. E. (2012). Biologically relevant mechanism for catalytic superoxide removal by simple manganese compounds. Proc. Natl. Acad. Sci. U.S.A. 109 (18), 6892–6897. doi: 10.1073/pnas.1203051109 22505740 PMC3344976

[B28] Barwinska-Sendra A. Garcia Y. M. Sendra K. M. Basle A. Mackenzie E. S. Tarrant E.. (2020). An evolutionary path to altered cofactor specificity in a metalloenzyme. Nat. Commun. 11 (1). doi: 10.1038/s41467-020-16478-0 PMC7264356 32483131

[B29] Bashir Q. Rashid N. Jamil F. Imanaka T. Akhtar M. (2009). Highly thermostable L-threonine dehydrogenase from the hyperthermophilic archaeon *Thermococcus kodakaraensis* . J. Biochem. 146 (1), 95–102. doi: 10.1093/jb/mvp051 19307254

[B30] Beaman B. L. Scates S. M. Moring S. E. Deem R. Misra H. P. (1983). Purification and properties of a unique superoxide dismutase from *Nocardia asteroides* . J. Biol. Chem. 258 (1), 91–96. doi: 10.1016/S0021-9258(18)33224-1 6336758

[B31] Beauchene N. A. Mettert E. L. Moore L. J. Keles S. Willey E. R. Kiley P. J. (2017). O_2_ availability impacts iron homeostasis in *Escherichia coli* . Proc. Natl. Acad. Sci. U.S.A. 114 (46), 12261–12266. doi: 10.1073/pnas.1707189114 29087312 PMC5699043

[B32] Becker A. Schlichting I. Kabsch W. Groche D. Schultz S. Wagner A. (1998). Iron center, substrate recognition and mechanism of peptide deformylase. Nat. Struct. Biol. 5 (12), 1053–1058. doi: 10.1038/4162 9846875

[B33] Beckman J. S. Koppenol W. H. (1996). Nitric oxide, superoxide, and peroxynitrite: the good, the bad, and ugly. Am. J. Physiol. 271 (5 Pt 1), C1424–C1437. doi: 10.1152/ajpcell.1996.271.5.C1424 8944624

[B34] Behnsen J. Zhi H. Aron A. T. Subramanian V. Santus W. Lee M. H.. (2021). Siderophore-mediated zinc acquisition enhances enterobacterial colonization of the inflamed gut. Nat. Commun. 12 (1), 7016. doi: 10.1038/s41467-021-27297-2 34853318 PMC8636617

[B35] Bellapadrona G. Ardini M. Ceci P. Stefanini S. Chiancone E. (2010). Dps proteins prevent Fenton-mediated oxidative damage by trapping hydroxyl radicals within the protein shell. Free Radical Biol. Med. 48 (2), 292–297. doi: 10.1016/j.freeradbiomed.2009.10.053 19892013

[B36] Benov L. (2001). How superoxide radical damages the cell. Protoplasma 217 (1-3), 33–36. doi: 10.1007/BF01289410 11732335

[B37] Besold A. N. Culbertson E. M. Culotta V. C. (2016). The yin and yang of copper during infection. J. Biol. Inorg Chem. 21 (2), 137–144. doi: 10.1007/s00775-016-1335-1 26790881 PMC5535265

[B38] Besold A. N. Gilston B. A. Radin J. N. Ramsoomair C. Culbertson E. M. Li C. X.. (2018). Role of calprotectin in withholding zinc and copper from *Candida albicans* . Infect. Immun. 86 (2). doi: 10.1128/IAI.00779-17 PMC5778358 29133349

[B39] Beyer W. F. Fridovich I. (1991). *In vivo* competition between iron and manganese for occupancy of the active site region of the manganese-superoxide dismutase of *Escherichia coli* . J. Biol. Chem. 266 (1), 303–308. doi: 10.1016/S0021-9258(18)52435-2 1985901

[B40] Blaesi E. J. Palowitch G. M. Hu K. Kim A. J. Rose H. R. Alapati R.. (2018). Metal-free class Ie ribonucleotide reductase from pathogens initiates catalysis with a tyrosine-derived dihydroxyphenylalanine radical. Proc. Natl. Acad. Sci. 115 (40), 10022. doi: 10.1073/pnas.1811993115 30224458 PMC6176560

[B41] Boal A. K. Cotruvo J. A. Jr. Stubbe J. Rosenzweig A. C. (2010). Structural basis for activation of class Ib ribonucleotide reductase. Science 329 (5998), 1526–1530. doi: 10.1126/science.1190187 20688982 PMC3020666

[B42] Bollinger J. M. Chen S. Parkin S. E. Mangravite L. M. Ley B. A. Edmondson D. E.. (1997). Differential iron (II) affinity of the sites of the diiron cluster in protein R2 of *Escherichia coli* ribonucleotide reductase: Tracking the individual sites through the O_2_ activation sequence. J. Am. Chem. Soc. 119 (25), 5976–5977. doi: 10.1021/ja970319s

[B43] Bosma E. F. Rau M. H. van Gijtenbeek L. A. Siedler S. (2021). Regulation and distinct physiological roles of manganese in bacteria. FEMS Microbiol. Rev. 45 (6). doi: 10.1093/femsre/fuab028 PMC8632737 34037759

[B44] Botella H. Peyron P. Levillain F. Poincloux R. Poquet Y. Brandli I.. (2011). Mycobacterial P_1_-type ATPases mediate resistance to zinc poisoning in human macrophages. Cell Host Microbe 10 (3), 248–259. doi: 10.1016/j.chom.2011.08.006 21925112 PMC3221041

[B45] Boyer E. Bergevin I. Malo D. Gros P. Cellier M. F. (2002). Acquisition of Mn(II) in addition to Fe(II) is required for full virulence of *Salmonella enterica* serovar Typhimurium. Infect. Immun. 70 (11), 6032–6042. doi: 10.1128/IAI.70.11.6032-6042.2002 12379679 PMC130432

[B46] Bradley J. M. Svistunenko D. A. Wilson M. T. Hemmings A. M. Moore G. R. Le Brun N. E. (2020). Bacterial iron detoxification at the molecular level. J. Biol. Chem. 295 (51), 17602–17623. doi: 10.1074/jbc.REV120.007746 33454001 PMC7762939

[B47] Brandenburg F. Schoffman H. Kurz S. Krämer U. Keren N. Weber A. P. M.. (2017). The *Synechocystis* manganese exporter Mnx is essential for manganese homeostasis in cyanobacteria. Plant Physiol. 173 (3), 1798–1810. doi: 10.1104/pp.16.01895 28153926 PMC5338678

[B48] Breckau D. Mahlitz E. Sauerwald A. Layer G. Jahn D. (2003). Oxygen-dependent coproporphyrinogen III oxidase (HemF) from *Escherichia coli* is stimulated by manganese. J. Biol. Chem. 278 (47), 46625–46631. doi: 10.1074/jbc.M308553200 12975365

[B49] Britton L. Malinowski D. P. Fridovich I. (1978). Superoxide dismutase and oxygen metabolism in *Streptococcus faecalis* and comparisons with other organisms. J. Bacteriol 134 (1), 229–236. doi: 10.1128/jb.134.1.229-236.1978 206536 PMC222239

[B50] Brophy M. B. Nakashige T. G. Gaillard A. Nolan E. M. (2013). Contributions of the S100A9 C-terminal tail to high-affinity Mn(II) chelation by the host-defense protein human calprotectin. J. Am. Chem. Soc. 135 (47), 17804–17817. doi: 10.1021/ja407147d 24245608 PMC3892207

[B51] Bunting K. Cooper J. B. Badasso M. O. Tickle I. J. Newton M. Wood S. P.. (1998). Engineering a change in metal-ion specificity of the iron-dependent superoxide dismutase from *Mycobacterium tuberculosis*– X-ray structure analysis of site-directed mutants. Eur. J. Biochem. 251 (3), 795–803. doi: 10.1046/j.1432-1327.1998.2510795.x 9490054

[B52] Capek J. Prochazkova I. Matousek T. Hot D. Vecerek B. (2021). A unique reverse adaptation mechanism assists *Bordetella pertussis* in resistance to both scarcity and toxicity of manganese. mBio 12 (5), e0190221. doi: 10.1128/mBio.01902-21 34700381 PMC8546581

[B53] Carlioz A. Touati D. (1986). Isolation of superoxide dismutase mutants in *Escherichia coli*: is superoxide dismutase necessary for aerobic life? EMBO J. 5 (3), 623–630. doi: 10.1002/j.1460-2075.1986.tb04256.x 3011417 PMC1166808

[B54] Caruthers J. Bosch J. Buckner F. Van Voorhis W. Myler P. Worthey E.. (2006). Structure of a ribulose 5-phosphate 3-epimerase from *Plasmodium falciparum* . Proteins 62 (2), 338–342. doi: 10.1002/prot.20764 16304640

[B55] Case A. J. (2017). On the origin of superoxide dismutase: An evolutionary perspective of superoxide-mediated redox signaling. Antioxidants (Basel) 6 (4). doi: 10.3390/antiox6040082 PMC5745492 29084153

[B56] Cellier M. F. Bergevin I. Boyer E. Richer E. (2001). Polyphyletic origins of bacterial Nramp transporters. Trends Genet. 17 (7), 365–370. doi: 10.1016/s0168-9525(01)02364-2 11418195

[B57] Cellier M. F. Courville P. Campion C. (2007). Nramp1 phagocyte intracellular metal withdrawal defense. Microbes infection 9 (14-15), 1662–1670. doi: 10.1016/j.micinf.2007.09.006 18024118

[B58] Chan M. K. Gong W. Rajagopalan P. T. Hao B. Tsai C. M. Pei D. (1997). Crystal structure of the *Escherichia coli* peptide deformylase. Biochemistry 36 (45), 13904–13909. doi: 10.1021/bi9711543 9374869

[B59] Charney J. Fisher W. P. Hegarty C. P. (1951). Managanese as an essential element for sporulation in the genus *Bacillus* . J. bacteriology 62 (2), 145–148. doi: 10.1128/jb.62.2.145-148.1951 PMC386101 14861172

[B60] Chen L. Helmann J. D. (1995). *Bacillus subtilis* MrgA is a Dps(PexB) homologue: evidence for metalloregulation of an oxidative-stress gene. Mol. Microbiol. 18 (2), 295–300. doi: 10.1111/j.1365-2958.1995.mmi_18020295.x 8709848

[B61] Christodoulou D. Link H. Fuhrer T. Kochanowski K. Gerosa L. Sauer U. (2018). Reserve flux capacity in the pentose phosphate pathway enables *Escherichia coli*'s rapid response to oxidative stress. Cell Syst. 6 (5), 569–578.e567. doi: 10.1016/j.cels.2018.04.009 29753645

[B62] Clohessy P. A. Golden B. E. (1995). Calprotectin-mediated zinc chelation as a biostatic mechanism in host defence. Scand. J. Immunol. 42 (5), 551–556. doi: 10.1111/j.1365-3083.1995.tb03695.x 7481561

[B63] Corbin B. D. Seeley E. H. Raab A. Feldmann J. Miller M. R. Torres V. J.. (2008). Metal chelation and inhibition of bacterial growth in tissue abscesses. Science 319 (5865), 962–965. doi: 10.1126/science.1152449 18276893

[B64] Cotruvo J. A. Jr. Stich T. A. Britt R. D. Stubbe J. (2013). Mechanism of assembly of the dimanganese-tyrosyl radical cofactor of class Ib ribonucleotide reductase: enzymatic generation of superoxide is required for tyrosine oxidation *via* a Mn(III)Mn(IV) intermediate. J. Am. Chem. Soc. 135 (10), 4027–4039. doi: 10.1021/ja312457t 23402532 PMC3739481

[B65] Cotruvo J. A. Jr. Stubbe J. (2010). An active dimanganese(III)-tyrosyl radical cofactor in *Escherichia coli* class Ib ribonucleotide reductase. Biochemistry 49 (6), 1297–1309. doi: 10.1021/bi902106n 20070127 PMC3190568

[B66] Cotruvo J. A. Stubbe J. (2011). *Escherichia coli* class Ib ribonucleotide reductase contains a Dimanganese(III)-tyrosyl radical cofactor in vivo. Biochemistry 50 (10), 1672–1681. doi: 10.1021/bi101881d 21250660 PMC3076206

[B67] Cotruvo J. A. Jr. Stubbe J. (2012). Metallation and mismetallation of iron and manganese proteins *in vitro* and *in vivo*: the class I ribonucleotide reductases as a case study. Metallomics 4 (10), 1020–1036. doi: 10.1039/c2mt20142a 22991063 PMC3488304

[B68] Cox N. Ogata H. Stolle P. Reijerse E. Auling G. Lubitz W. (2010). A tyrosyl–dimanganese coupled spin system is the native metalloradical cofactor of the R2F subunit of the ribonucleotide reductase of *Corynebacterium ammoniagenes* . J. Am. Chem. Soc. 132 (32), 11197–11213. doi: 10.1021/ja1036995 20698687

[B69] Cross P. J. Pietersma A. L. Allison T. M. Wilson-Coutts S. M. Cochrane F. C. Parker E. J. (2013). *Neisseria meningitidis* expresses a single 3-deoxy-D-arabino-heptulosonate 7-phosphate synthase that is inhibited primarily by phenylalanine. Protein Sci. 22 (8), 1087–1099. doi: 10.1002/pro.2293 23754471 PMC3832045

[B70] Cubillas C. Vinuesa P. Luisa Tabche M. Dávalos A. Vázquez A. Hernández-Lucas I.. (2014). The cation diffusion facilitator protein EmfA of *Rhizobium etli* belongs to a novel subfamily of Mn^2+^/Fe^2+^ transporters conserved in α-proteobacteria. Metallomics 6 (10), 1808–1815. doi: 10.1039/C4MT00135D 25054342

[B71] Cybulski R. J. Jr. Sanz P. Alem F. Stibitz S. Bull R. L. O'Brien A. D. (2009). Four superoxide dismutases contribute to *Bacillus anthracis* virulence and provide spores with redundant protection from oxidative stress. Infect. Immun. 77 (1), 274–285. doi: 10.1128/IAI.00515-08 18955476 PMC2612278

[B72] Dailey H. A. (1987). Metal inhibition of ferrochelatase. Ann. N Y Acad. Sci. 514, 81–86. doi: 10.1111/j.1749-6632.1987.tb48763.x 3442391

[B73] Dale I. Fagerhol M. K. Naesgaard I. (1983). Purification and partial characterization of a highly immunogenic human leukocyte protein, the L1 antigen. Eur. J. Biochem. 134 (1), 1–6. doi: 10.1111/j.1432-1033.1983.tb07522.x 6861753

[B74] Daly M. J. Gaidamakova E. K. Matrosova V. Y. Kiang J. G. Fukumoto R. Lee D. Y.. (2010). Small-molecule antioxidant proteome-shields in *Deinococcus radiodurans* . PloS One 5 (9), e12570. doi: 10.1371/journal.pone.0012570 20838443 PMC2933237

[B75] Daly M. J. Gaidamakova E. K. Matrosova V. Y. Vasilenko A. Zhai M. Venkateswaran A.. (2004). Accumulation of Mn(II) in *Deinococcus radiodurans* facilitates gamma-radiation resistance. Science 306 (5698), 1025–1028. doi: 10.1126/science.1103185 15459345

[B76] Davidsson L. Lönnerdal B. Sandström B. Kunz C. Keen C. L. (1989). Identification of transferrin as the major plasma carrier protein for manganese introduced orally or intravenously or after *in vitro* addition in the rat. J. Nutr. 119 (10), 1461–1464. doi: 10.1093/jn/119.10.1461 2585137

[B77] DeShazer D. Barnnan J. D. Moran M. J. Friedman R. L. (1994). Characterization of the gene encoding superoxide dismutase of *Bordetella pertussis* and construction of a SOD-deficient mutant. Gene 142 (1), 85–89. doi: 10.1016/0378-1119(94)90359-X 8181762

[B78] De Vendittis A. Amato M. Mickniewicz A. Parlato G. De Angelis A. Castellano I.. (2010). Regulation of the properties of superoxide dismutase from the dental pathogenic microorganism *Streptococcus mutans* by iron- and manganese-bound co-factor. Mol. Biosyst. 6 (10), 1973–1982. doi: 10.1039/c003557b 20672178

[B79] Diaz-Ochoa V. E. Lam D. Lee C. S. Klaus S. Behnsen J. Liu J. Z.. (2016). Salmonella mitigates oxidative stress and thrives in the inflamed gut by evading calprotectin-mediated manganese sequestration. Cell Host Microbe 19 (6), 814–825. doi: 10.1016/j.chom.2016.05.005 27281571 PMC4901528

[B80] Dosselaere F. Vanderleyden J. (2001). A metabolic node in action: Chorismate-utilizing enzymes in microorganisms. Crit. Rev. Microbiol. 27 (2), 75–131. doi: 10.1080/20014091096710 11450855

[B81] Duckworth O. W. Sposito G. (2005). Siderophore–Manganese(III) interactions. i. air-oxidation of Manganese(II) promoted by desferrioxamine B. Environ. Sci. Technol. 39 (16), 6037–6044. doi: 10.1021/es050275k 16173561

[B82] Dudev T. Lim C. (2014). Competition among metal ions for protein binding sites: determinants of metal ion selectivity in proteins. Chem. Rev. 114 (1), 538–556. doi: 10.1021/cr4004665 24040963

[B83] Edge R. Truscott T. G. (2018). Singlet oxygen and free radical reactions of retinoids and carotenoids-A review. Antioxidants (Basel) 7 (1). doi: 10.3390/antiox7010005 PMC5789315 29301252

[B84] Eijkelkamp B. A. Morey J. R. Ween M. P. Ong C. L. McEwan A. G. Paton J. C.. (2014). Extracellular zinc competitively inhibits manganese uptake and compromises oxidative stress management in *Streptococcus pneumoniae* . PloS One 9 (2), e89427. doi: 10.1371/journal.pone.0089427 24558498 PMC3928430

[B85] El Shafey H. M. Ghanem S. Merkamm M. Guyonvarch A. (2008). *Corynebacterium glutamicum* superoxide dismutase is a manganese-strict non-cambialistic enzyme *in vitro* . Microbiol. Res. 163 (1), 80–86. doi: 10.1016/j.micres.2006.05.005 16809027

[B86] Epperly B. R. Dekker E. E. (1991). L-threonine dehydrogenase from *Escherichia coli*. Identification of an active site cysteine residue and metal ion studies. J. Biol. Chem. 266 (10), 6086–6092. doi: 10.1016/S0021-9258(18)38087-6 2007567

[B87] Eriksson M. Jordan A. Eklund H. (1998). Structure of *Salmonella* Typhimurium nrdF ribonucleotide reductase in its oxidized and reduced forms. Biochemistry 37 (38), 13359–13369. doi: 10.1021/bi981380s 9748343

[B88] Ernst F. D. Homuth G. Stoof J. Mader U. Waidner B. Kuipers E. J.. (2005). Iron-responsive regulation of the *Helicobacter pylori* iron-cofactored superoxide dismutase SodB is mediated by Fur. J. Bacteriol 187 (11), 3687–3692. doi: 10.1128/JB.187.11.3687-3692.2005 15901691 PMC1112043

[B89] Fagerhol M. K. Dale I. Anderson T. (1980). Release and quantitation of a leucocyte derived protein (L1). Scandinavian J. Haematology 24 (5), 393–398. doi: 10.1111/j.1600-0609.1980.tb02754.x

[B90] Fanali G. Cao Y. Ascenzi P. Fasano M. (2012). Mn(II) binding to human serum albumin: A 1H-NMR relaxometric study. J. Inorganic Biochem. 117, 198–203. doi: 10.1016/j.jinorgbio.2012.08.013 23099538

[B91] Farkas E. Szabo O. Parajdi-Losonczi P. L. Balla G. Pocsi I. (2014). Mn(II)/Mn(III) and Fe(III) binding capability of two *Aspergillus fumigatus* siderophores, desferricrocin and N', N'', N'''-triacetylfusarinine C. J. Inorg Biochem. 139, 30–37. doi: 10.1016/j.jinorgbio.2014.06.005 24959697

[B92] Faulkner K. M. Stevens R. D. Fridovich I. (1994). Characterization of Mn(III) complexes of linear and cyclic desferrioxamines as mimics of superoxide dismutase activity. Arch. Biochem. Biophysics 310 (2), 341–346. doi: 10.1006/abbi.1994.1176 8179317

[B93] Fell J. S. Steele D. M. Hatcher T. C. Gherman B. F. (2015). Electronic effects on the reaction mechanism of the metalloenzyme peptide deformylase. Theor. Chem. Accounts 134 (5), 1–7. doi: 10.1007/s00214-015-1674-y

[B94] Fisher C. R. Wyckoff E. E. Peng E. D. Payne S. M. (2016). Identification and characterization of a putative manganese export protein in *Vibrio cholerae* . J. bacteriology 198 (20), 2810–2817. doi: 10.1128/JB.00215-16 PMC5038002 27481926

[B95] Fitsanakis V. A. Zhang N. Garcia S. Aschner M. (2010). Manganese (Mn) and iron (Fe): interdependency of transport and regulation. Neurotox Res. 18 (2), 124–131. doi: 10.1007/s12640-009-9130-1 19921534 PMC7909719

[B96] Flint D. H. Tuminello J. F. Emptage M. H. (1993). The inactivation of Fe-S cluster containing hydro-lyases by superoxide. J. Biol. Chem. 268 (30), 22369–22376. doi: 10.1016/S0021-9258(18)41538-4 8226748

[B97] Forbes J. R. Gros P. (2001). Divalent-metal transport by NRAMP proteins at the interface of host-pathogen interactions. Trends Microbiol. 9 (8), 397–403. doi: 10.1016/s0966-842x(01)02098-4 11514223

[B98] Forbes J. R. Gros P. (2003). Iron, manganese, and cobalt transport by Nramp1 (Slc11a1) and Nramp2 (Slc11a2) expressed at the plasma membrane. Blood 102 (5), 1884–1892. doi: 10.1182/blood-2003-02-0425 12750164

[B99] Fridovich I. (2013). Oxygen: How do we stand it? Med. Principles Pract. 22 (2), 131–137. doi: 10.1159/000339212 PMC5685332 22759590

[B100] Frye K. A. Sendra K. M. Waldron K. J. Kehl-Fie T. E. (2022). Old dogs, new tricks: New insights into the iron/manganese superoxide dismutase family. J. Inorganic Biochem. 230, 111748. doi: 10.1016/j.jinorgbio.2022.111748 PMC9112591 35151099

[B101] Furdui C. Zhou L. Woodard R. W. Anderson K. S. (2004). Insights into the mechanism of 3-deoxy-D-arabino-heptulosonate 7-phosphate synthase (Phe) from *Escherichia coli* using a transient kinetic analysis. J. Biol. Chem. 279 (44), 45618–45625. doi: 10.1074/jbc.M404753200 15326172

[B102] Gardner P. R. Fridovich I. (1991). Superoxide sensitivity of the *Escherichia coli* aconitase. J. Biol. Chem. 266 (29), 19328–19333. doi: 10.1016/S0021-9258(18)55001-8 1655783

[B103] Gerlach D. Reichardt W. Vettermann S. (1998). Extracellular superoxide dismutase from *Streptococcus pyogenes* type 12 strain is manganese-dependent. FEMS Microbiol. Lett. 160 (2), 217–224. doi: 10.1111/j.1574-6968.1998.tb12914.x 9532741

[B104] Girotti A. W. (1990). Photodynamic lipid peroxidation in biological systems. Photochem. Photobiol. 51 (4), 497–509. doi: 10.1111/j.1751-1097.1990.tb01744.x 2188273

[B105] Golonka R. Yeoh B. S. Vijay-Kumar M. (2019). The iron tug-of-war between bacterial siderophores and innate immunity. J. Innate Immun. 11 (3), 249–262. doi: 10.1159/000494627 30605903 PMC6487204

[B106] Graeff-Wohlleben H. Killat S. Banemann A. Guiso N. Gross R. (1997). Cloning and characterization of an Mn-containing superoxide dismutase (SodA) of *Bordetella pertussis* . J. Bacteriol 179 (7), 2194–2201. doi: 10.1128/jb.179.7.2194-2201.1997 9079904 PMC178955

[B107] Grage K. (2005). “Oxygen-independent coproporphyrinogen III oxidase,” in Characterization of *Escherichia coli* HemN and investigation of proposed functional analogs Dissertation (Braunschweig, Germany: Universität Carolo-Wilhelmina).

[B108] Grāve K. Griese J. J. Berggren G. Bennett M. D. Högbom M. (2020). The *Bacillus anthracis* class Ib ribonucleotide reductase subunit NrdF intrinsically selects manganese over iron. JBIC J. Biol. Inorganic Chem. 25 (4), 571–582. doi: 10.1007/s00775-020-01782-3 PMC7239806 32296998

[B109] Griese J. J. Roos K. Cox N. Shafaat H. S. Branca R. M. Lehtio J.. (2013). Direct observation of structurally encoded metal discrimination and ether bond formation in a heterodinuclear metalloprotein. Proc. Natl. Acad. Sci. U.S.A. 110 (43), 17189–17194. doi: 10.1073/pnas.1304368110 24101498 PMC3808653

[B110] Groche D. Becker A. Schlichting I. Kabsch W. Schultz S. Wagner A. V. (1998). Isolation and crystallization of functionally competent *Escherichia coli* peptide deformylase forms containing either iron or nickel in the active site. Biochem. Biophys. Res. Commun. 246 (2), 342–346. doi: 10.1006/bbrc.1998.8616 9610360

[B111] Grunenwald C. M. Choby J. E. Juttukonda L. J. Beavers W. N. Weiss A. Torres V. J.. (2019). Manganese detoxification by MntE is critical for resistance to oxidative stress and virulence of *Staphylococcus aureus* . mBio 10 (1), e02915–e02918. doi: 10.1128/mBio.02915-18 30808698 PMC6391924

[B112] Guedon E. Helmann J. D. (2003). Origins of metal ion selectivity in the DtxR/MntR family of metalloregulators. Mol. Microbiol. 48 (2), 495–506. doi: 10.1046/j.1365-2958.2003.03445.x 12675807

[B113] Gu M. Imlay J. A. (2013). Superoxide poisons mononuclear iron enzymes by causing mismetallation. Mol. Microbiol. 89 (1), 123–134. doi: 10.1111/mmi.12263 23678969 PMC3731988

[B114] Gunter T. E. Gerstner B. Gunter K. K. Malecki J. Gelein R. Valentine W. M.. (2013). Manganese transport *via* the transferrin mechanism. NeuroToxicology 34, 118–127. doi: 10.1016/j.neuro.2012.10.018 23146871 PMC3576891

[B115] Haber F. Weiss J. Pope W. J. (1934). The catalytic decomposition of hydrogen peroxide by iron salts. Proc. R. Soc. London. Ser. A - Math. Phys. Sci. 147 (861), 332–351. doi: 10.1098/rspa.1934.0221

[B116] Hackam D. J. Rotstein O. D. Zhang W. Gruenheid S. Gros P. Grinstein S. (1998). Host resistance to intracellular infection: mutation of natural resistance-associated macrophage protein 1 (Nramp1) impairs phagosomal acidification. J. Exp. Med. 188 (2), 351–364. doi: 10.1084/jem.188.2.351 9670047 PMC2212455

[B117] Hall R. S. Fedorov A. A. Xu C. Fedorov E. V. Almo S. C. Raushel F. M. (2011). Three-dimensional structure and catalytic mechanism of cytosine deaminase. Biochemistry 50 (22), 5077–5085. doi: 10.1021/bi200483k 21545144 PMC3107989

[B118] Halliwell B. Gutteridge J. M. (1992). Biologically relevant metal ion-dependent hydroxyl radical generation. An update. FEBS Lett. 307 (1), 108–112. doi: 10.1016/0014-5793(92)80911-y 1322323

[B119] Hammerstad M. Røhr Å.K. Andersen N. H. Gräslund A. Högbom M. Andersson K. K. (2014). The class Ib ribonucleotide reductase from *Mycobacterium tuberculosis* has two active R2F subunits. JBIC J. Biol. Inorganic Chem. 19 (6), 893–902. doi: 10.1007/s00775-014-1121-x 24585102

[B120] Handing K. B. Shabalin I. G. Kassaar O. Khazaipoul S. Blindauer C. A. Stewart A. J.. (2016). Circulatory zinc transport is controlled by distinct interdomain sites on mammalian albumins. Chem. Sci. 7 (11), 6635–6648. doi: 10.1039/c6sc02267g 28567254 PMC5450522

[B121] Hantke K. (1987). Selection procedure for deregulated iron transport mutants (fur) in *Escherichia coli* K 12: fur not only affects iron metabolism. Mol. Gen. Genet. 210 (1), 135–139. doi: 10.1007/BF00337769 3323834

[B122] Harrington J. M. Parker D. L. Bargar J. R. Jarzecki A. A. Tebo B. M. Sposito G.. (2012). Structural dependence of Mn complexation by siderophores: donor group dependence on complex stability and reactivity. Geochimica Cosmochimica Acta 88, 106–119. doi: 10.1016/j.gca.2012.04.006

[B123] Harris W. R. Chen Y. (1994). Electron paramagnetic resonance and difference ultraviolet studies of Mn^2+^ binding to serum transferrin. J. Inorganic Biochem. 54 (1), 1–19. doi: 10.1016/0162-0134(94)85119-0 8151309

[B124] Harvie D. R. Vílchez S. Steggles J. R. Ellar D. J. (2005). *Bacillus cereus* fur regulates iron metabolism and is required for full virulence. Microbiol. (Reading) 151 (Pt 2), 569–577. doi: 10.1099/mic.0.27744-0 15699205

[B125] Hassett D. J. Howell M. L. Ochsner U. A. Vasil M. L. Johnson Z. Dean G. E. (1997). An operon containing fumC and sodA encoding fumarase C and manganese superoxide dismutase is controlled by the ferric uptake regulator in *Pseudomonas aeruginosa*: fur mutants produce elevated alginate levels. J. Bacteriol 179 (5), 1452–1459. doi: 10.1128/jb.179.5.1452-1459.1997 9045799 PMC178852

[B126] Häuslein I. Manske C. Goebel W. Eisenreich W. Hilbi H. (2016). Pathway analysis using 13C-glycerol and other carbon tracers reveals a bipartite metabolism of *Legionella pneumophila* . Mol. Microbiol. 100 (2), 229–246. doi: 10.1111/mmi.13313 26691313

[B127] Heinzen R. A. Frazier M. E. Mallavia L. P. (1992). *Coxiella burnetii* superoxide dismutase gene: cloning, sequencing, and expression in *Escherichia coli* . Infect. Immun. 60 (9), 3814–3823. doi: 10.1128/iai.60.9.3814-3823.1992 1500190 PMC257394

[B128] Henriques A. O. Melsen L. R. Moran C. P. Jr (1998). Involvement of superoxide dismutase in spore coat assembly in *Bacillus subtilis* . J. Bacteriol 180 (9), 2285–2291. doi: 10.1128/JB.180.9.2285-2291.1998 9573176 PMC107166

[B129] Hider R. C. Kong X. (2010). Chemistry and biology of siderophores. Nat. Prod Rep. 27 (5), 637–657. doi: 10.1039/b906679a 20376388

[B130] Higashi N. Tanimoto K. Nishioka M. Ishikawa K. Taya M. (2008). Investigating a catalytic mechanism of hyperthermophilic L-threonine dehydrogenase from *Pyrococcus horikoshii* . J. Biochem. 144 (1), 77–85. doi: 10.1093/jb/mvn041 18390572

[B131] Hiraoka B. Y. Yamakura F. Sugio S. Nakayama K. (2000). A change of the metal-specific activity of a cambialistic superoxide dismutase from *Porphyromonas gingivalis* by a double mutation of gln-70 to gly and ala-142 to gln. Biochem. J. 345 Pt 2, 345–350. doi: 10.1042/bj3450345 10620511 PMC1220763

[B132] Hogbom M. Galander M. Andersson M. Kolberg M. Hofbauer W. Lassmann G.. (2003). Displacement of the tyrosyl radical cofactor in ribonucleotide reductase obtained by single-crystal high-field EPR and 1.4-Å x-ray data. Proc. Natl. Acad. Sci. U.S.A. 100 (6), 3209–3214. doi: 10.1073/pnas.0536684100 12624184 PMC404301

[B133] Hogbom M. Stenmark P. Voevodskaya N. McClarty G. Graslund A. Nordlund P. (2004). The radical site in chlamydial ribonucleotide reductase defines a new R2 subclass. Science 305 (5681), 245–248. doi: 10.1126/science.1098419 15247479

[B134] Honarmand Ebrahimi K. Hagedoorn P. L. Hagen W. R. (2015). Unity in the biochemistry of the iron-storage proteins ferritin and bacterioferritin. Chem. Rev. 115 (1), 295–326. doi: 10.1021/cr5004908 25418839

[B135] Hosseinzadeh P. Lu Y. (2016). Design and fine-tuning redox potentials of metalloproteins involved in electron transfer in bioenergetics. Biochim. Biophys. Acta (BBA) - Bioenergetics 1857 (5), 557–581. doi: 10.1016/j.bbabio.2015.08.006 26301482 PMC4761536

[B136] Huang X. Shin J.-H. Pinochet-Barros A. Su T. T. Helmann J. D. (2017). *Bacillus subtilis* MntR coordinates the transcriptional regulation of manganese uptake and efflux systems. Mol. Microbiol. 103 (2), 253–268. doi: 10.1111/mmi.13554 27748968 PMC5218975

[B137] Huang S.-H. Wang C.-K. Peng H.-L. Wu C.-C. Chen Y.-T. Hong Y.-M.. (2012). Role of the small RNA RyhB in the fur regulon in mediating the capsular polysaccharide biosynthesis and iron acquisition systems in *Klebsiella pneumoniae* . BMC Microbiol. 12 (1), 148. doi: 10.1186/1471-2180-12-148 22827802 PMC3423075

[B138] Imlay J. A. (2006). Iron-sulphur clusters and the problem with oxygen. Mol. Microbiol. 59 (4), 1073–1082. doi: 10.1111/j.1365-2958.2006.05028.x 16430685

[B139] Imlay J. A. (2013). The molecular mechanisms and physiological consequences of oxidative stress: lessons from a model bacterium. Nat. Rev. Microbiol. 11 (7), 443–454. doi: 10.1038/nrmicro3032 23712352 PMC4018742

[B140] Imlay J. A. (2014). The mismetallation of enzymes during oxidative stress. J. Biol. Chem. 289 (41), 28121–28128. doi: 10.1074/jbc.R114.588814 25160623 PMC4192467

[B141] Inaoka T. Matsumura Y. Tsuchido T. (1999). SodA and manganese are essential for resistance to oxidative stress in growing and sporulating cells of *Bacillus subtilis* . J. Bacteriol 181 (6), 1939–1943. doi: 10.1128/JB.181.6.1939-1943.1999 10074093 PMC93599

[B142] Ireton G. C. Black M. E. Stoddard B. L. (2003). The 1.14 Å crystal structure of yeast cytosine deaminase: Evolution of nucleotide salvage enzymes and implications for genetic chemotherapy. Structure 11 (8), 961–972. doi: 10.1016/S0969-2126(03)00153-9 12906827

[B143] Ireton G. C. McDermott G. Black M. E. Stoddard B. L. (2002a). The structure of *Escherichia coli* cytosine deaminase. J. Mol. Biol. 315 (4), 687–697. doi: 10.1006/jmbi.2001.5277 11812140

[B144] Ireton G. C. McDermott G. Black M. E. Stoddard B. L. (2002b). The structure of *Escherichia coli* cytosine deaminase. J. Mol. Biol. 315 (4), 687–697. doi: 10.1006/jmbi.2001.5277 11812140

[B145] Irving H. Williams R. J. P. (1948). Order of stability of metal complexes. Nature 162, 746–747. doi: 10.1038/162746a0

[B146] Jacques J.-F. Jang S. Prévost K. Desnoyers G. Desmarais M. Imlay J.. (2006). RyhB small RNA modulates the free intracellular iron pool and is essential for normal growth during iron limitation in *Escherichia coli* . Mol. Microbiol. 62 (4), 1181–1190. doi: 10.1111/j.1365-2958.2006.05439.x 17078818

[B147] Jang S. Imlay J. A. (2007). Micromolar intracellular hydrogen peroxide disrupts metabolism by damaging iron-sulfur enzymes. J. Biol. Chem. 282 (2), 929–937. doi: 10.1074/jbc.M607646200 17102132 PMC5136138

[B148] Jang S. Imlay J. A. (2010). Hydrogen peroxide inactivates the *Escherichia coli* Isc iron-sulphur assembly system, and OxyR induces the Suf system to compensate. Mol. Microbiol. 78 (6), 1448–1467. doi: 10.1111/j.1365-2958.2010.07418.x 21143317 PMC3051806

[B149] Jayachandran M. Yoon J. Wu J. Cipurko D. Quon J. Makhlynets O. (2021). Mechanistic studies of the cofactor assembly in class Ib ribonucleotide reductases and protein affinity for MnII and FeII. Metallomics 13 (11). doi: 10.1093/mtomcs/mfab062 34718709

[B150] Jelakovic S. Kopriva S. Süss K.-H. Schulz G. E. (2003). Structure and catalytic mechanism of the cytosolic D-Ribulose-5-phosphate 3-epimerase from rice. J. Mol. Biol. 326 (1), 127–135. doi: 10.1016/S0022-2836(02)01374-8 12547196

[B151] Jensen W. B. (1978). The Lewis acid-base definitions: a status report. Chemistry 78 (1), 1–22. doi: 10.1021/cr60311a002

[B152] Jiang W. Yun D. Saleh L. Barr E. W. Xing G. Hoffart L. M.. (2007). A manganese(IV)/iron(III) cofactor in *Chlamydia trachomatis* ribonucleotide reductase. Science 316 (5828), 1188–1191. doi: 10.1126/science.1141179 17525338

[B153] John J. Aurelius O. Srinivas V. Saura P. Kim I. S. Bhowmick A.. (2022). Redox-controlled reorganization and flavin strain within the ribonucleotide reductase R2b-NrdI complex monitored by serial femtosecond crystallography. Elife 11. doi: 10.7554/eLife.79226 PMC9462851 36083619

[B154] Johne B. Fagerhol M. K. Lyberg T. Prydz H. Brandtzaeg P. Naess-Andresen C. F.. (1997). Functional and clinical aspects of the myelomonocyte protein calprotectin. Mol. Pathol. 50 (3), 113–123. doi: 10.1136/mp.50.3.113 9292145 PMC379605

[B155] Johnson A. R. Chen Y. W. Dekker E. E. (1998). Investigation of a catalytic zinc binding site in *Escherichia coli* L-threonine dehydrogenase by site-directed mutagenesis of cysteine-38. Arch. Biochem. Biophys. 358 (2), 211–221. doi: 10.1006/abbi.1998.0845 9784233

[B156] Johnsrude M. J. Pitzer J. E. Martin D. W. Roop R. M. (2019). The cation diffusion facilitator family protein EmfA confers resistance to manganese toxicity in *Brucella abortus* 2308 and is an essential virulence determinant in mice. J. Bacteriol 202 (1), e00357–e00319. doi: 10.1128/JB.00357-19 31591273 PMC6932235

[B157] Jursa T. Smith D. R. (2009). Ceruloplasmin alters the tissue disposition and neurotoxicity of manganese, but not its loading onto transferrin. Toxicol. Sci. 107 (1), 182–193. doi: 10.1093/toxsci/kfn231 19005224 PMC2735423

[B158] Juttukonda L. J. Skaar E. P. (2015). Manganese homeostasis and utilization in pathogenic bacteria. Mol. Microbiol. 97 (2), 216–228. doi: 10.1111/mmi.13034 25898914 PMC4631260

[B159] Kehres D. G. Janakiraman A. Slauch J. M. Maguire M. E. (2002). Regulation of *Salmonella enterica* serovar Typhimurium mntH transcription by H(2)O(2), Fe(2+), and Mn(2+). J. Bacteriol 184 (12), 3151–3158. doi: 10.1128/JB.184.12.3151-3158.2002 12029030 PMC135095

[B160] Kehres D. G. Zaharik M. L. Finlay B. B. Maguire M. E. (2000). The NRAMP proteins of *Salmonella* Typhimurium and *Escherichia coli* are selective manganese transporters involved in the response to reactive oxygen. Mol. Microbiol. 36 (5), 1085–1100. doi: 10.1046/j.1365-2958.2000.01922.x 10844693

[B161] Kelliher J. Kehl-Fie T. (2016). Competition for manganese at the host–pathogen interface. Prog. Mol. Biol. Trans. Sci. 142, 1–25. doi: 10.1016/bs.pmbts.2016.05.002 27571690

[B162] Keyer K. Imlay J. A. (1996). Superoxide accelerates DNA damage by elevating free-iron levels. Proc. Natl. Acad. Sci. U.S.A. 93 (24), 13635–13640. doi: 10.1073/pnas.93.24.13635 8942986 PMC19375

[B163] Kimoto R. Funahashi T. Yamamoto N. Miyoshi S.-i. Narimatsu S. Yamamoto S. (2001). Identification and characterization of the sodA genes encoding manganese superoxide dismutases in *Vibrio parahaemolyticus, Vibrio mimicus*, and *Vibrio vulnificus* . Microbiol. Immunol. 45 (2), 135–142. doi: 10.1111/j.1348-0421.2001.tb01281.x 11293479

[B164] Kisgeropoulos E. C. Griese J. J. Smith Z. R. Branca R. M. M. Schneider C. R. Hogbom M.. (2020). Key structural motifs balance metal binding and oxidative reactivity in a heterobimetallic Mn/Fe protein. J. Am. Chem. Soc. 142 (11), 5338–5354. doi: 10.1021/jacs.0c00333 32062969 PMC7390604

[B165] König V. Pfeil A. Braus G. H. Schneider T. R. (2004). Substrate and metal complexes of 3-Deoxy-D-arabino-heptulosonate-7-phosphate synthase from *Saccharomyces cerevisiae* provide new insights into the catalytic mechanism. J. Mol. Biol. 337 (3), 675–690. doi: 10.1016/j.jmb.2004.01.055 15019786

[B166] Koppenol W. H. (1993). The centennial of the Fenton reaction. Free Radical Biol. Med. 15 (6), 645–651. doi: 10.1016/0891-5849(93)90168-T 8138191

[B167] Koppenol W. H. Butler J. (1977). Mechanism of reactions involving singlet oxygen and the superoxide anion. FEBS Lett. 83 (1), 1–6. doi: 10.1016/0014-5793(77)80628-5 200483

[B168] Koppenol W. H. Stanbury D. M. Bounds P. L. (2010). Electrode potentials of partially reduced oxygen species, from dioxygen to water. Free Radic. Biol. Med. 49 (3), 317–322. doi: 10.1016/j.freeradbiomed.2010.04.011 20406682

[B169] Kraemer S. M. (2004). Iron oxide dissolution and solubility in the presence of siderophores. Aquat. Sci. 66 (1), 3–18. doi: 10.1007/s00027-003-0690-5

[B170] Krauss I. R. Merlino A. Pica A. Rullo R. Bertoni A. Capasso A.. (2015). Fine tuning of metal-specific activity in the Mn-like group of cambialistic superoxide dismutases. RSC Adv. 5 (107), 87876–87887. doi: 10.1039/C5RA13559A

[B171] Kroll J. S. Wilks K. E. Farrant J. L. Langford P. R. (1998). Natural genetic exchange between *Haemophilus* and *Neisseria*: intergeneric transfer of chromosomal genes between major human pathogens. Proc. Natl. Acad. Sci. U.S.A. 95 (21), 12381–12385. doi: 10.1073/pnas.95.21.12381 9770495 PMC22840

[B172] Kuehne A. Emmert H. Soehle J. Winnefeld M. Fischer F. Wenck H.. (2015). Acute activation of oxidative pentose phosphate pathway as first-line response to oxidative stress in human skin cells. Mol. Cell 59 (3), 359–371. doi: 10.1016/j.molcel.2015.06.017 26190262

[B173] Kuo C. F. Mashino T. Fridovich I. (1987). Alpha, beta-dihydroxyisovalerate dehydratase. a superoxide-sensitive enzyme. J. Biol. Chem. 262 (10), 4724–4727. doi: 10.1016/S0021-9258(18)61255-4 3031031

[B174] Kwak Y. Jiang W. Dassama L. M. Park K. Bell C. B. Liu L. V.. (2013). Geometric and electronic structure of the Mn(IV)Fe(III) cofactor in class Ic ribonucleotide reductase: correlation to the class Ia binuclear non-heme iron enzyme. J. Am. Chem. Soc. 135 (46), 17573–17584. doi: 10.1021/ja409510d 24131208 PMC3882272

[B175] Labbé R. F. Vreman H. J. Stevenson D. K. (1999). Zinc protoporphyrin: A metabolite with a mission. Clin. Chem. 45 (12), 2060–2072. doi: 10.1093/clinchem/45.12.2060 10585337

[B176] Ladomersky E. Khan A. Shanbhag V. Cavet J. S. Chan J. Weisman G. A.. (2017). Host and pathogen copper-transporting P-type ATPases function antagonistically during *Salmonella* infection. Infect. Immun. 85 (9), e00351–e00317. doi: 10.1128/IAI.00351-17 28652309 PMC5563570

[B177] Lalaouna D. Baude J. Wu Z. Tomasini A. Chicher J. Marzi S.. (2019). RsaC sRNA modulates the oxidative stress response of *Staphylococcus aureus* during manganese starvation. Nucleic Acids Res. 47 (18), 9871–9887. doi: 10.1093/nar/gkz728 31504767 PMC6765141

[B178] Lam L. N. Wong J. J. Chong K. K. L. Kline K. A. (2020). *Enterococcus faecalis* manganese exporter MntE alleviates manganese toxicity and is required for mouse gastrointestinal colonization. Infect. Immun. 88 (6), e00058–e00020. doi: 10.1128/IAI.00058-20 32229614 PMC7240088

[B179] Lang P. F. Smith B. C. (2003). Ionization energies of atoms and atomic ions. J. Chem. Educ. 80 (8), 938–946. doi: 10.1021/ed080p938

[B180] Lapinskas P. J. Cunningham K. W. Liu X. F. Fink G. R. Culotta V. C. (1995). Mutations in PMR1 suppress oxidative damage in yeast cells lacking superoxide dismutase. Mol. Cell Biol. 15 (3), 1382–1388. doi: 10.1128/MCB.15.3.1382 7862131 PMC230362

[B181] Letendre E. D. Holbein B. E. (1984). Ceruloplasmin and regulation of transferrin iron during *Neisseria meningitidis* infection in mice. Infection Immun. 45 (1), 133–138. doi: 10.1128/iai.45.1.133-138.1984 PMC263289 6429041

[B182] Liang W. Ouyang S. Shaw N. Joachimiak A. Zhang R. Liu Z.-J. (2011). Conversion of D-ribulose 5-phosphate to D-xylulose 5-phosphate: new insights from structural and biochemical studies on human RPE. FASEB J. Off. Publ. Fed. Am. Societies Exp. Biol. 25 (2), 497–504. doi: 10.1096/fj.10-171207 PMC6188353 20923965

[B183] Lingappa U. F. Monteverde D. R. Magyar J. S. Valentine J. S. Fischer W. W. (2019). How manganese empowered life with dioxygen (and vice versa). Free Radical Biol. Med. 140, 113–125. doi: 10.1016/j.freeradbiomed.2019.01.036 30738765

[B184] Li C. Tao J. Mao D. He C. (2011). A novel manganese efflux system, YebN, is required for virulence by *Xanthomonas oryzae* pv. oryzae. PloS One 6 (7), e21983. doi: 10.1371/journal.pone.0021983 21789199 PMC3136493

[B185] Li W. Wang H. Lei C. Ying T. Tan X. (2015). Manganese superoxide dismutase from human pathogen *Clostridium difficile* . Amino Acids 47 (5), 987–995. doi: 10.1007/s00726-015-1927-z 25655385

[B186] Li W. Wang H. Wang Q. Tan X. (2014). Structural, spectroscopic and functional investigation into Fe-substituted MnSOD from human pathogen *Clostridium difficile* . Metallomics 6 (8), 1540–1548. doi: 10.1039/c4mt00090k 24915901

[B187] Loprasert S. Sallabhan R. Whangsuk W. Mongkolsuk S. (2000). Characterization and mutagenesis of fur gene from *Burkholderia pseudomallei* . Gene 254 (1-2), 129–137. doi: 10.1016/s0378-1119(00)00279-1 10974543

[B188] Machielsen R. van der Oost J. (2006). Production and characterization of a thermostable L-threonine dehydrogenase from the hyperthermophilic archaeon *Pyrococcus furiosus* . FEBS J. 273 (12), 2722–2729. doi: 10.1111/j.1742-4658.2006.05290.x 16817900

[B189] Madison A. S. Tebo B. M. Mucci A. Sundby B. Luther G. W. 3rd (2013). Abundant porewater Mn(III) is a major component of the sedimentary redox system. Science 341, 875–878. doi: 10.1126/science.1241396 23970696

[B190] Majtan T. Frerman F. E. Kraus J. P. (2011). Effect of cobalt on *Escherichia coli* metabolism and metalloporphyrin formation. Biometals 24 (2), 335–347. doi: 10.1007/s10534-010-9400-7 21184140 PMC3071258

[B191] Makhlynets O. Boal A. K. Rhodes D. V. Kitten T. Rosenzweig A. C. Stubbe J. (2014). *Streptococcus sanguinis* class Ib ribonucleotide reductase: high activity with both iron and manganese cofactors and structural insights. J. Biol. Chem. 289 (9), 6259–6272. doi: 10.1074/jbc.M113.533554 24381172 PMC3937692

[B192] Mancini S. Imlay J. A. (2015). The induction of two biosynthetic enzymes helps *Escherichia coli* sustain heme synthesis and activate catalase during hydrogen peroxide stress. Mol. Microbiol. 96 (4), 744–763. doi: 10.1111/mmi.12967 25664592 PMC4430354

[B193] Manley O. M. Phan H. N. Stewart A. K. Mosley D. A. Xue S. Cha L.. (2022). Self-sacrificial tyrosine cleavage by an Fe:Mn oxygenase for the biosynthesis of para-aminobenzoate in *Chlamydia trachomatis* . Proc. Natl. Acad. Sci. U.S.A. 119 (39), e2210908119. doi: 10.1073/pnas.2210908119 36122239 PMC9522330

[B194] Manta B. Raushel F. M. Himo F. (2014). Reaction mechanism of zinc-dependent cytosine deaminase from *Escherichia coli*: A quantum-chemical study. J. Phys. Chem. B 118 (21), 5644–5652. doi: 10.1021/jp501228s 24833316

[B195] Martin J. E. Imlay J. A. (2011). The alternative aerobic ribonucleotide reductase of *Escherichia coli*, NrdEF, is a manganese-dependent enzyme that enables cell replication during periods of iron starvation. Mol. Microbiol. 80 (2), 319–334. doi: 10.1111/j.1365-2958.2011.07593.x 21338418 PMC3097424

[B196] Martin J. E. Le M. T. Bhattarai N. Capdevila D. A. Shen J. Winkler M. E.. (2019). A Mn-sensing riboswitch activates expression of a Mn^2+^/Ca^2+^ ATPase transporter in *Streptococcus* . Nucleic Acids Res. 47 (13), 6885–6899. doi: 10.1093/nar/gkz494 31165873 PMC6649816

[B197] Martins M. C. Romão C. V. Folgosa F. Borges P. T. Frazão C. Teixeira M. (2019). How superoxide reductases and flavodiiron proteins combat oxidative stress in anaerobes. Free Radical Biol. Med. 140, 36–60. doi: 10.1016/j.freeradbiomed.2019.01.051 30735841

[B198] Masse E. Gottesman S. (2002). A small RNA regulates the expression of genes involved in iron metabolism in *Escherichia coli* . Proc. Natl. Acad. Sci. U.S.A. 99 (7), 4620–4625. doi: 10.1073/pnas.032066599 11917098 PMC123697

[B199] Masse E. Vanderpool C. K. Gottesman S. (2005). Effect of RyhB small RNA on global iron use in *Escherichia coli* . J. Bacteriol 187 (20), 6962–6971. doi: 10.1128/JB.187.20.6962-6971.2005 16199566 PMC1251601

[B200] McArthur J. D. West N. P. Cole J. N. Jungnitz H. Guzman C. A. Chin J.. (2003). An aromatic amino acid auxotrophic mutant of *Bordetella bronchiseptica* is attenuated and immunogenic in a mouse model of infection. FEMS Microbiol. Lett. 221 (1), 7–16. doi: 10.1016/S0378-1097(03)00162-9 12694904

[B201] McGuire A. M. Cuthbert B. J. Ma Z. Grauer-Gray K. D. Brunjes Brophy M. Spear K. A.. (2013). Roles of the A and C sites in the manganese-specific activation of MntR. Biochemistry 52 (4), 701–713. doi: 10.1021/bi301550t 23298157 PMC3562352

[B202] McLeod M. P. Qin X. Karpathy S. E. Gioia J. Highlander S. K. Fox G. E.. (2004). Complete genome sequence of *Rickettsia typhi* and comparison with sequences of other rickettsiae. J. Bacteriol 186 (17), 5842–5855. doi: 10.1128/JB.186.17.5842-5855.2004 15317790 PMC516817

[B203] McNaughton R. L. Reddi A. R. Clement M. H. Sharma A. Barnese K. Rosenfeld L.. (2010). Probing *in vivo* Mn^2+^ speciation and oxidative stress resistance in yeast cells with electron-nuclear double resonance spectroscopy. Proc. Natl. Acad. Sci. U.S.A. 107 (35), 15335–15339. doi: 10.1073/pnas.1009648107 20702768 PMC2932569

[B204] Medlock A. E. Carter M. Dailey T. A. Dailey H. A. Lanzilotta W. N. (2009). Product release rather than chelation determines metal specificity for ferrochelatase. J. Mol. Biol. 393 (2), 308–319. doi: 10.1016/j.jmb.2009.08.042 19703464 PMC2771925

[B205] Meinnel T. Blanquet S. (1993). Evidence that peptide deformylase and methionyl-tRNA(fMet) formyltransferase are encoded within the same operon in *Escherichia coli* . J. Bacteriol 175 (23), 7737–7740. doi: 10.1128/jb.175.23.7737-7740.1993 8244948 PMC206938

[B206] Mey A. R. Craig S. A. Payne S. M. (2005). Characterization of *Vibrio cholerae* RyhB: the RyhB regulon and role of ryhB in biofilm formation. Infect. Immun. 73 (9), 5706–5719. doi: 10.1128/IAI.73.9.5706-5719.2005 16113288 PMC1231101

[B207] Miller A. F. (2008). Redox tuning over almost 1 V in a structurally conserved active site: lessons from Fe-containing superoxide dismutase. Acc Chem. Res. 41 (4), 501–510. doi: 10.1021/ar700237u 18376853

[B208] Miller A. F. (2012). Superoxide dismutases: ancient enzymes and new insights. FEBS Lett. 586 (5), 585–595. doi: 10.1016/j.febslet.2011.10.048 22079668 PMC5443681

[B209] Mizuno K. Whittaker M. M. Bächinger H. P. Whittaker J. W. (2004). Calorimetric studies on the tight binding metal interactions of *Escherichia coli* manganese superoxide dismutase. J. Biol. Chem. 279 (26), 27339–27344. doi: 10.1074/jbc.M400813200 15082717

[B210] Murphy E. R. Payne S. M. (2007). RyhB, an iron-responsive small RNA molecule, regulates *Shigella dysenteriae* virulence. Infection Immun. 75 (7), 3470–3477. doi: 10.1128/IAI.00112-07 PMC1932958 17438026

[B211] Najmuldeen H. Alghamdi R. Alghofaili F. Yesilkaya H. (2019). Functional assessment of microbial superoxide dismutase isozymes suggests a differential role for each isozyme. Free Radical Biol. Med. 134, 215–228. doi: 10.1016/j.freeradbiomed.2019.01.018 30658083

[B212] Nakashige T. G. Zhang B. Krebs C. Nolan E. M. (2015). Human calprotectin is an iron-sequestering host-defense protein. Nat. Chem. Biol. 11 (10), 765–771. doi: 10.1038/nchembio.1891 26302479 PMC4575267

[B213] Nakashige T. G. Zygiel E. M. Drennan C. L. Nolan E. M. (2017). Nickel sequestration by the host-defense protein human calprotectin. J. Am. Chem. Soc. 139 (26), 8828–8836. doi: 10.1021/jacs.7b01212 28573847 PMC5754018

[B214] Narasimhan J. Letinski S. Jung S. P. Gerasyuto A. Wang J. Arnold M.. (2022). Ribonucleotide reductase, a novel drug target for gonorrhea. Elife 11. doi: 10.7554/eLife.67447 PMC8865847 35137690

[B215] Natesha R. K. Natesha R. Victory D. Barnwell S. P. Hoover E. L. (1992). A prognostic role for ceruloplasmin in the diagnosis of indolent and recurrent inflammation. J. Natl. Med. Assoc. 84 (9), 781–784. 1404475 PMC2571777

[B216] Neville S. L. Sjöhamn J. Watts J. A. MacDermott-Opeskin H. Fairweather S. J. Ganio K.. (2021). The structural basis of bacterial manganese import. Sci. Adv. 7 (32). doi: 10.1126/sciadv.abg3980. PMC8346216 34362732

[B217] Oglesby A. G. Murphy E. R. Iyer V. R. Payne S. M. (2005). Fur regulates acid resistance in *Shigella flexneri via* RyhB and ydeP. Mol. Microbiol. 58 (5), 1354–1367. doi: 10.1111/j.1365-2958.2005.04920.x 16313621

[B218] Orban K. Finkel S. E. Maupin-Furlow J. A. (2022). Dps is a universally conserved dual-action DNA-binding and ferritin protein. J. Bacteriology 204 (5), e00036–e00022. doi: 10.1128/jb.00036-22 PMC9112962 35380871

[B219] Ouyang A. Gasner K. M. Neville S. L. McDevitt C. A. Frawley E. R. (2022). MntP and YiiP contribute to manganese efflux in *Salmonella enterica* serovar Typhimurium under conditions of manganese overload and nitrosative stress. Microbiol. Spectr. 10 (1), e0131621. doi: 10.1128/spectrum.01316-21 35019706 PMC8754126

[B220] Padilla-Benavides T. Long J. E. Raimunda D. Sassetti C. M. Argüello J. M. (2013). A novel P(1B)-type Mn^2+^-transporting ATPase is required for secreted protein metallation in mycobacteria. J. Biol. Chem. 288 (16), 11334–11347. doi: 10.1074/jbc.M112.448175 23482562 PMC3630897

[B221] Panosa A. Roca I. Gibert I. (2010). Ribonucleotide reductases of *Salmonella* Typhimurium: transcriptional regulation and differential role in pathogenesis. PloS One 5 (6), e11328. doi: 10.1371/journal.pone.0011328 20593029 PMC2892513

[B222] Paruthiyil S. Pinochet-Barros A. Huang X. Helmann J. D. (2020). *Bacillus subtilis* TerC family proteins help prevent manganese intoxication. J. Bacteriol 202 (2), e00624–e00619. doi: 10.1128/JB.00624-19 31685536 PMC6941523

[B223] Passalacqua K. D. Bergman N. H. Herring-Palmer A. Hanna P. (2006). The superoxide dismutases of *Bacillus anthracis* do not cooperatively protect against endogenous superoxide stress. J. Bacteriol 188 (11), 3837–3848. doi: 10.1128/JB.00239-06 16707676 PMC1482891

[B224] Pearson R. G. (1963). Hard and soft acids and bases. J. Am. Chem. Soc. 85 (22), 3533–3539. doi: 10.1021/ja00905a001

[B225] Peng E. D. Lyman L. R. Schmitt M. P. (2021). Analysis of the manganese and MntR regulon in *Corynebacterium diphtheriae* . J. Bacteriol 203 (20), e0027421. doi: 10.1128/JB.00274-21 34370555 PMC8459757

[B226] Permpoonpattana P. Phetcharaburanin J. Mikelsone A. Dembek M. Tan S. Brisson M. C.. (2013). Functional characterization of *Clostridium difficile* spore coat proteins. J. Bacteriol 195 (7), 1492–1503. doi: 10.1128/jb.02104-12 23335421 PMC3624542

[B227] Persson B. Hedlund J. Jörnvall H. (2008). Medium- and short-chain dehydrogenase/reductase gene and protein families. Cell. Mol. Life Sci. 65 (24), 3879–3894. doi: 10.1007/s00018-008-8587-z 19011751 PMC2792335

[B228] Pieper R. Huang S.-T. Parmar P. P. Clark D. J. Alami H. Fleischmann R. D.. (2010). Proteomic analysis of iron acquisition, metabolic and regulatory responses of *Yersinia pestis* to iron starvation. BMC Microbiol. 10 (1), 30. doi: 10.1186/1471-2180-10-30 20113483 PMC2835676

[B229] Plapp B. V. (2010). Conformational changes and catalysis by alcohol dehydrogenase. Arch. Biochem. Biophys. 493 (1), 3–12. doi: 10.1016/j.abb.2009.07.001 19583966 PMC2812590

[B230] Porter D. J. Austin E. A. (1993). Cytosine deaminase. The roles of divalent metal ions in catalysis. J. Biol. Chem. 268 (32), 24005–24011. doi: 10.1016/S0021-9258(20)80485-2 8226944

[B231] Price S. L. Vadyvaloo V. DeMarco J. K. Brady A. Gray P. A. Kehl-Fie T. E.. (2021). Yersiniabactin contributes to overcoming zinc restriction during *Yersinia pestis* infection of mammalian and insect hosts. Proc. Natl. Acad. Sci. U.S.A. 118 (44). doi: 10.1073/pnas.2104073118 PMC8612365 34716262

[B232] Priebe G. P. Brinig M. M. Hatano K. Grout M. Coleman F. T. Pier G. B.. (2002). Construction and characterization of a live, attenuated aroA deletion mutant of *Pseudomonas aeruginosa* as a candidate intranasal vaccine. Infect. Immun. 70 (3), 1507–1517. doi: 10.1128/IAI.70.3.1507-1517.2002 11854239 PMC127764

[B233] Rabin O. Hegedus L. Bourre J.-M. Smith Q. R. (1993). Rapid brain uptake of Manganese(II) across the blood-brain barrier. J. Neurochemistry 61 (2), 509–517. doi: 10.1111/j.1471-4159.1993.tb02153.x 7687654

[B234] Rada B. Leto T. L. (2008). Oxidative innate immune defenses by Nox/Duox family NADPH oxidases. Contributions to Microbiol. 15, 164–187. doi: 10.1159/000136357 PMC2776633 18511861

[B235] Ragusa S. Blanquet S. Meinnel T. (1998). Control of peptide deformylase activity by metal cations. J. Mol. Biol. 280 (3), 515–523. doi: 10.1006/jmbi.1998.1883 9665853

[B236] Raimunda D. Elso-Berberián G. (2014). Functional characterization of the CDF transporter SMc02724 (SmYiiP) in *Sinorhizobium meliloti*: Roles in manganese homeostasis and nodulation. Biochim. Biophys. Acta (BBA) - Biomembranes 1838 (12), 3203–3211. doi: 10.1016/j.bbamem.2014.09.005 25242380

[B237] Rajagopalan P. T. Pei D. (1998). Oxygen-mediated inactivation of peptide deformylase. J. Biol. Chem. 273 (35), 22305–22310. doi: 10.1074/jbc.273.35.22305 9712848

[B238] Rajagopalan P. T. R. Yu X. C. Pei D. (1997). Peptide deformylase: a new type of mononuclear iron protein. J. Am. Chem. Soc. 119 (50), 12418–12419. doi: 10.1021/ja9734096

[B239] Reinhart A. A. Powell D. A. Nguyen A. T. O'Neill M. Djapgne L. Wilks A.. (2015). The prrF-encoded small regulatory RNAs are required for iron homeostasis and virulence of *Pseudomonas aeruginosa* . Infect. Immun. 83 (3), 863–875. doi: 10.1128/IAI.02707-14 25510881 PMC4333466

[B240] Rhodes D. V. Crump K. E. Makhlynets O. Snyder M. Ge X. Xu P.. (2014). Genetic characterization and role in virulence of the ribonucleotide reductases of *Streptococcus sanguinis* . J. Biol. Chem. 289 (9), 6273–6287. doi: 10.1074/jbc.M113.533620 24381171 PMC3937693

[B241] Richardson A. R. Somerville G. A. Sonenshein A. L. (2015). Regulating the intersection of metabolism and pathogenesis in Gram-positive bacteria. Microbiol. Spectr. 3 (3), 3.3.11. doi: 10.1128/microbiolspec.MBP-0004-2014 PMC4540601 26185086

[B242] Robinett N. G. Peterson R. L. Culotta V. C. (2018). Eukaryotic copper-only superoxide dismutases (SODs): A new class of SOD enzymes and SOD-like protein domains. J. Biol. Chem. 293 (13), 4636–4643. doi: 10.1074/jbc.TM117.000182 29259135 PMC5880121

[B243] Robinson N. J. Glasfeld A. (2020). Metalation: nature's challenge in bioinorganic chemistry. J. Biol. Inorg Chem. 25 (4), 543–545. doi: 10.1007/s00775-020-01790-3 32333210 PMC7239837

[B244] Roca I. Torrents E. Sahlin M. Gibert I. Sjoberg B. M. (2008). NrdI essentiality for class Ib ribonucleotide reduction in *Streptococcus pyogenes* . J. Bacteriol 190 (14), 4849–4858. doi: 10.1128/JB.00185-08 18502861 PMC2447006

[B245] Roeser H. P. Lee G. R. Nacht S. Cartwright G. E. (1970). The role of ceruloplasmin in iron metabolism. J. Clin. Invest. 49 (12), 2408–2417. doi: 10.1172/JCI106460 5480864 PMC322742

[B246] Roos K. Siegbahn P. E. (2009). Density functional theory study of the manganese-containing ribonucleotide reductase from *Chlamydia trachomatis*: why manganese is needed in the active complex. Biochemistry 48 (9), 1878–1887. doi: 10.1021/bi801695d 19220003

[B247] Roos K. Siegbahn P. E. (2011). Oxygen cleavage with manganese and iron in ribonucleotide reductase from *Chlamydia trachomatis* . J. Biol. Inorg Chem. 16 (4), 553–565. doi: 10.1007/s00775-011-0755-1 21258828

[B248] Rosch J. W. Gao G. Ridout G. Wang Y.-D. Tuomanen E. I. (2009). Role of the manganese efflux system mntE for signalling and pathogenesis in *Streptococcus pneumoniae* . Mol. Microbiol. 72 (1), 12–25. doi: 10.1111/j.1365-2958.2009.06638.x 19226324 PMC2706702

[B249] Rose H. R. Ghosh M. K. Maggiolo A. O. Pollock C. J. Blaesi E. J. Hajj V.. (2018). Structural basis for superoxide activation of *Flavobacterium johnsoniae* class I ribonucleotide reductase and for radical initiation by its dimanganese cofactor. Biochemistry 57 (18), 2679–2693. doi: 10.1021/acs.biochem.8b00247 29609464 PMC6488936

[B250] Rose H. R. Maggiolo A. O. McBride M. J. Palowitch G. M. Pandelia M. E. Davis K. M.. (2019). Structures of class Id ribonucleotide reductase catalytic subunits reveal a minimal architecture for deoxynucleotide biosynthesis. Biochemistry 58 (14), 1845–1860. doi: 10.1021/acs.biochem.8b01252 30855138 PMC6456427

[B251] Runyen-Janecky L. Dazenski E. Hawkins S. Warner L. (2006). Role and regulation of the *Shigella flexneri* Sit and MntH systems. Infect. Immun. 74 (8), 4666–4672. doi: 10.1128/IAI.00562-06 16861654 PMC1539580

[B252] Ruskoski T. B. Boal A. K. (2021). The periodic table of ribonucleotide reductases. J. Biol. Chem. 297 (4), 101137. doi: 10.1016/j.jbc.2021.101137 34461093 PMC8463856

[B253] Rytter H. Jamet A. Ziveri J. Ramond E. Coureuil M. Lagouge-Roussey P.. (2021). The pentose phosphate pathway constitutes a major metabolic hub in pathogenic *Francisella* . PloS Pathog. 17 (8), e1009326. doi: 10.1371/journal.ppat.1009326 34339477 PMC8360588

[B254] Sadosky A. B. Wilson J. W. Steinman H. M. Shuman H. A. (1994). The iron superoxide dismutase of *Legionella pneumophila* is essential for viability. J. Bacteriol 176 (12), 3790–3799. doi: 10.1128/jb.176.12.3790-3799.1994 8206858 PMC205569

[B255] Sánchez-Baracaldo P. Cardona T. (2020). On the origin of oxygenic photosynthesis and cyanobacteria. New Phytol. 225 (4), 1440–1446. doi: 10.1111/nph.16249 31598981

[B256] Schmidt F. Donahoe S. Hagens K. Mattow J. Schaible U. E. Kaufmann S. H.. (2004). Complementary analysis of the *Mycobacterium tuberculosis* proteome by two-dimensional electrophoresis and isotope-coded affinity tag technology. Mol. Cell Proteomics 3 (1), 24–42. doi: 10.1074/mcp.M300074-MCP200 14557599

[B257] Seaver L. C. Imlay J. A. (2001). Alkyl hydroperoxide reductase is the primary scavenger of endogenous hydrogen peroxide in *Escherichia coli* . J. Bacteriol 183 (24), 7173–7181. doi: 10.1128/JB.183.24.7173-7181.2001 11717276 PMC95566

[B258] Seib K. L. Tseng H. J. McEwan A. G. Apicella M. A. Jennings M. P. (2004). Defenses against oxidative stress in *Neisseria gonorrhoeae* and *Neisseria meningitidis*: distinctive systems for different lifestyles. J. Infect. Dis. 190 (1), 136–147. doi: 10.1086/421299 15195253

[B259] Sen A. Zhou Y. Imlay J. A. (2020). During oxidative stress the clp proteins of *Escherichia coli* ensure that iron pools remain sufficient to reactivate oxidized metalloenzymes. J. Bacteriol 202 (18). doi: 10.1128/JB.00235-20 PMC7925084 32601069

[B260] Seo S. W. Kim D. Szubin R. Palsson Bernhard O. (2015). Genome-wide reconstruction of OxyR and SoxRS transcriptional regulatory networks under oxidative stress in *Escherichia coli* K-12 MG1655. Cell Rep. 12 (8), 1289–1299. doi: 10.1016/j.celrep.2015.07.043 26279566

[B261] Sheng Y. Abreu I. A. Cabelli D. E. Maroney M. J. Miller A. F. Teixeira M.. (2014). Superoxide dismutases and superoxide reductases. Chem. Rev. 114 (7), 3854–3918. doi: 10.1021/cr4005296 24684599 PMC4317059

[B262] Shumilin I. A. Bauerle R. Wu J. Woodard R. W. Kretsinger R. H. (2004). Crystal structure of the reaction complex of 3-Deoxy-D-arabino-heptulosonate-7-phosphate synthase from *Thermotoga maritima* refines the catalytic mechanism and indicates a new mechanism of allosteric regulation. J. Mol. Biol. 341 (2), 455–466. doi: 10.1016/j.jmb.2004.05.077 15276836

[B263] Shumilin I. A. Kretsinger R. H. Bauerle R. H. (1999). Crystal structure of phenylalanine-regulated 3-deoxy-D-arabino-heptulosonate-7-phosphate synthase from *Escherichia coli* . Structure 7 (7), 865–875. doi: 10.1016/s0969-2126(99)80109-9 10425687

[B264] Singh P. Ali S. A. (2022). Multifunctional role of S100 protein family in the immune system: An update. Cells 11 (15). doi: 10.3390/cells11152274 PMC9332480 35892571

[B265] Skamene E. (1994). The bcg gene story. Immunobiology 191 (4), 451–460. doi: 10.1016/S0171-2985(11)80451-1 7713559

[B266] Skamene E. Schurr E. Gros P. (1998). Infection genomics: Nramp1 as a major determinant of natural resistance to intracellular infections. Annu. Rev. Med. 49 (1), 275–287. doi: 10.1146/annurev.med.49.1.275 9509263

[B267] Sobota J. M. Gu M. Imlay J. A. (2014). Intracellular hydrogen peroxide and superoxide poison 3-deoxy-D-arabinoheptulosonate 7-phosphate synthase, the first committed enzyme in the aromatic biosynthetic pathway of *Escherichia coli* . J. bacteriology 196 (11), 1980–1991. doi: 10.1128/JB.01573-14 PMC4010980 24659765

[B268] Sobota J. M. Imlay J. A. (2011). Iron enzyme ribulose-5-phosphate 3-epimerase in *Escherichia coli* is rapidly damaged by hydrogen peroxide but can be protected by manganese. Proc. Natl. Acad. Sci. U.S.A. 108 (13), 5402–5407. doi: 10.1073/pnas.1100410108 21402925 PMC3069151

[B269] Springer S. D. Butler A. (2015). Magnetic susceptibility of Mn(III) complexes of hydroxamate siderophores. J. Inorg Biochem. 148, 22–26. doi: 10.1016/j.jinorgbio.2015.04.015 25978931

[B270] Srinivas V. Lebrette H. Lundin D. Kutin Y. Sahlin M. Lerche M.. (2018). Metal-free ribonucleotide reduction powered by a DOPA radical in *Mycoplasma* pathogens. Nature 563 (7731), 416–420. doi: 10.1038/s41586-018-0653-6 30429545 PMC6317698

[B271] Sriranganathan N. Boyle S. M. Schurig G. Misra H. (1991). Superoxide dismutases of virulent and avirulent strains of *Brucella abortus* . Vet. Microbiol. 26 (4), 359–366. doi: 10.1016/0378-1135(91)90029-f 1903225

[B272] Stincone A. Prigione A. Cramer T. Wamelink M. M. C. Campbell K. Cheung E.. (2015). The return of metabolism: biochemistry and physiology of the pentose phosphate pathway. Biol. Rev. 90 (3), 927–963. doi: 10.1111/brv.12140 25243985 PMC4470864

[B273] Sun H. Xu G. Zhan H. Chen H. Sun Z. Tian B.. (2010). Identification and evaluation of the role of the manganese efflux protein in *Deinococcus radiodurans* . BMC Microbiol. 10 (1), 319. doi: 10.1186/1471-2180-10-319 21156049 PMC3016326

[B274] Supek F. Supekova L. Nelson H. Nelson N. (1996). A yeast manganese transporter related to the macrophage protein involved in conferring resistance to mycobacteria. Proc. Natl. Acad. Sci. U.S.A. 93 (10), 5105–5110. doi: 10.1073/pnas.93.10.5105 8643535 PMC39414

[B275] Sutherland K. M. Ward L. M. Colombero C. R. Johnston D. T. (2021). Inter-domain horizontal gene transfer of nickel-binding superoxide dismutase. Geobiology 19 (5), 450–459. doi: 10.1111/gbi.12448 33989454

[B276] Topolski A. (2011). Insight into the degradation of a manganese(III)-citrate complex in aqueous solutions. Chem. Papers 65 (3), 389–392. doi: 10.2478/s11696-010-0101-z

[B277] Touati D. Jacques M. Tardat B. Bouchard L. Despied S. (1995). Lethal oxidative damage and mutagenesis are generated by iron in delta fur mutants of *Escherichia coli*: protective role of superoxide dismutase. J. Bacteriol 177 (9), 2305–2314. doi: 10.1128/jb.177.9.2305-2314.1995 7730258 PMC176885

[B278] Tribe D. E. Camakaris H. Pittard J. (1976). Constitutive and repressible enzymes of the common pathway of aromatic biosynthesis in *Escherichia coli* K-12: regulation of enzyme synthesis at different growth rates. J. Bacteriol 127 (3), 1085–1097. doi: 10.1128/jb.127.3.1085-1097.1976 8426 PMC232899

[B279] Trouwborst R. E. Clement B. G. Tebo B. M. Glazer B. T. Luther G. W. 3rd (2006). Soluble Mn(III) in suboxic zones. Science 313, 1955–1957. doi: 10.1126/science.1132876 17008530

[B280] Tseng H.-J. Srikhanta Y. McEwan A. G. Jennings M. P. (2001). Accumulation of manganese in *Neisseria gonorrhoeae* correlates with resistance to oxidative killing by superoxide anion and is independent of superoxide dismutase activity. Mol. Microbiol. 40 (5), 1175–1186. doi: 10.1046/j.1365-2958.2001.02460.x 11401721

[B281] Tsolis R. M. Baumler A. J. Heffron F. (1995). Role of *Salmonella* Typhimurium Mn-superoxide dismutase (SodA) in protection against early killing by J774 macrophages. Infect. Immun. 63 (5), 1739–1744. doi: 10.1128/iai.63.5.1739-1744.1995 7729880 PMC173218

[B282] Tu W. Y. Pohl S. Gray J. Robinson N. J. Harwood C. R. Waldron K. J. (2012). Cellular iron distribution in *Bacillus anthracis* . J. Bacteriol 194 (5), 932–940. doi: 10.1128/JB.06195-11 22178968 PMC3294808

[B283] Turner A. G. Ong C.-L. Y. Gillen C. M. Davies M. R. West N. P. McEwan A. G.. (2015). Manganese homeostasis in group A *Streptococcus* is critical for resistance to oxidative stress and virulence. mBio 6 (2), e00278–e00215. doi: 10.1128/mBio.00278-15 25805729 PMC4453566

[B284] Vance C. K. Miller A.-F. (2001). Novel insights into the basis for *Escherichia coli* superoxide dismutase's metal ion specificity from Mn-substituted FeSOD and its very high *E* _m_ . Biochemistry 40 (43), 13079–13087. doi: 10.1021/bi0113317 11669646

[B285] Vasantha N. Freese E. (1979). The role of manganese in growth and sporulation of *Bacillus subtilis* . Microbiology 112 (2), 329–336. doi: 10.1099/00221287-112-2-329 225409

[B286] Vecerek B. Moll I. Afonyushkin T. Kaberdin V. Blasi U. (2003). Interaction of the RNA chaperone hfq with mRNAs: direct and indirect roles of hfq in iron metabolism of *Escherichia coli* . Mol. Microbiol. 50 (3), 897–909. doi: 10.1046/j.1365-2958.2003.03727.x 14617150

[B287] Verneuil N. Maze A. Sanguinetti M. Laplace J. M. Benachour A. Auffray Y.. (2006). Implication of (Mn)superoxide dismutase of *Enterococcus faecalis* in oxidative stress responses and survival inside macrophages. Microbiol. (Reading) 152 (Pt 9), 2579–2589. doi: 10.1099/mic.0.28922-0 16946253

[B288] Veyrier F. J. Boneca I. G. Cellier M. F. Taha M.-K. (2011). A novel metal transporter mediating manganese export (MntX) regulates the Mn to Fe intracellular ratio and *Neisseria meningitidis* virulence. PloS Pathog. 7 (9), e1002261. doi: 10.1371/journal.ppat.1002261 21980287 PMC3182930

[B289] Vidal S. M. Malo D. Vogan K. Skamene E. Gros P. (1993). Natural resistance to infection with intracellular parasites: Isolation of a candidate for bcg. Cell 73 (3), 469–485. doi: 10.1016/0092-8674(93)90135-D 8490962

[B290] Voegtli W. C. Sommerhalter M. Saleh L. Baldwin J. Bollinger J. M. Jr. Rosenzweig A. C. (2003). Variable coordination geometries at the diiron(II) active site of ribonucleotide reductase R2. J. Am. Chem. Soc. 125 (51), 15822–15830. doi: 10.1021/ja0370387 14677973

[B291] Wang Y. Mo X. Zhang L. Wang Q. (2011). Four superoxide dismutase (isozymes) genes of *Bacillus cereus* . Ann. Microbiol. 61 (2), 355–360. doi: 10.1007/s13213-010-0149-6

[B292] Wang Y. Wang H. Yang C. H. Wang Q. Mei R. (2007). Two distinct manganese-containing superoxide dismutase genes in *Bacillus cereus*: their physiological characterizations and roles in surviving in wheat rhizosphere. FEMS Microbiol. Lett. 272 (2), 206–213. doi: 10.1111/j.1574-6968.2007.00759.x 17521361

[B293] Wan B. Zhang Q. Ni J. Li S. Wen D. Li J.. (2017). Type VI secretion system contributes to enterohemorrhagic *Escherichia coli* virulence by secreting catalase against host reactive oxygen species (ROS). PloS Pathog. 13 (3), e1006246. doi: 10.1371/journal.ppat.1006246 28288207 PMC5363993

[B294] Wasselin V. Staerck C. Rince I. Leger L. Budin-Verneuil A. Hartke A.. (2021). Characterisation of the manganese superoxide dismutase of *Enterococcus faecium* . Res. Microbiol. 172 (6), 103876. doi: 10.1016/j.resmic.2021.103876 34474124

[B295] Waters L. S. Sandoval M. Storz G. (2011). The *Escherichia coli* MntR miniregulon includes genes encoding a small protein and an efflux pump required for manganese homeostasis. J. Bacteriol 193 (21), 5887–5897. doi: 10.1128/JB.05872-11 21908668 PMC3194919

[B296] Weinberg E. D. (1975). Nutritional immunity: Host's attempt to withhold iron from microbial invaders. JAMA 231 (1), 39–41. doi: 10.1001/jama.1975.03240130021018 1243565

[B297] White C. Lee J. Kambe T. Fritsche K. Petris M. J. (2009). A role for the ATP7A copper-transporting ATPase in macrophage bactericidal activity. J. Biol. Chem. 284 (49), 33949–33956. doi: 10.1074/jbc.M109.070201 19808669 PMC2797165

[B298] Whittaker M. M. Mizuno K. Bachinger H. P. Whittaker J. W. (2006). Kinetic analysis of the metal binding mechanism of *Escherichia coli* manganese superoxide dismutase. Biophys. J. 90 (2), 598–607. doi: 10.1529/biophysj.105.071308 16258041 PMC1367064

[B299] Winterbourn C. C. (2020). Biological chemistry of superoxide radicals. ChemTexts 6 (1), 7. doi: 10.1007/s40828-019-0101-8

[B300] Wu X. H. Quan J. M. Wu Y. D. (2007). Theoretical study of the catalytic mechanism and metal-ion dependence of peptide deformylase. J. Phys. Chem. B 111 (22), 6236–6244. doi: 10.1021/jp068611m 17497768

[B301] Wu C. H. H. Tsai-Wu J.-J. Huang Y.-T. Lin C.-Y. Lioua G.-G. Lee F.-J. S. (1998). Identification and subcellular localization of a novel Cu,Zn superoxide dismutase of *Mycobacterium tuberculosis* . FEBS Lett. 439 (1), 192–196. doi: 10.1016/S0014-5793(98)01373-8 9849904

[B302] Xu J. Zheng C. Cao M. Zeng T. Zhao X. Shi G.. (2017). The manganese efflux system MntE contributes to the virulence of *Streptococcus suis* serotype 2. Microbial Pathogenesis 110, 23–30. doi: 10.1016/j.micpath.2017.06.022 28629722

[B303] Yamakura F. Kobayashi K. Tagawa S. Morita A. Imai T. Ohmori D.. (1995). pH-dependent activity change of superoxide dismutase from *Mycobacterium smegmatis* . Biochem. Mol. Biol. Int. 36 (2), 233–240. 7663426

[B304] Yang J. Bitoun J. P. Ding H. (2006). Interplay of IscA and IscU in biogenesis of iron-sulfur clusters. J. Biol. Chem. 281 (38), 27956–27963. doi: 10.1074/jbc.M601356200 16877383

[B305] Yun Y. S. Lee Y. N. (2004). Purification and some properties of superoxide dismutase from *Deinococcus radiophilus*, the UV-resistant bacterium. Extremophiles 8 (3), 237–242. doi: 10.1007/s00792-004-0383-6 15106001

[B306] Yu J. Yu X. Liu J. (2004). A thermostable manganese-containing superoxide dismutase from pathogen *Chlamydia pneumoniae* . FEBS Lett. 562 (1-3), 22–26. doi: 10.1016/S0014-5793(04)00170-X 15043996

[B307] Zeinert R. Martinez E. Schmitz J. Senn K. Usman B. Anantharaman V.. (2018). Structure-function analysis of manganese exporter proteins across bacteria. J. Biol. Chem. 293 (15), 5715–5730. doi: 10.1074/jbc.M117.790717 29440394 PMC5900781

[B308] Zhang Y. Stubbe J. (2011). *Bacillus subtilis* class Ib ribonucleotide reductase is a dimanganese(III)-tyrosyl radical enzyme. Biochemistry 50 (25), 5615–5623. doi: 10.1021/bi200348q 21561096 PMC3130199

[B309] Zhang J. Wang H. Huang Q. Zhang Y. Zhao L. Liu F.. (2020). Four superoxide dismutases of *Bacillus cereus* 0–9 are non-redundant and perform different functions in diverse living conditions. World J. Microbiol. Biotechnol. 36 (1), 12. doi: 10.1007/s11274-019-2786-7 31897767

[B310] Zhao G. Ceci P. Ilari A. Giangiacomo L. Laue T. M. Chiancone E.. (2002). Iron and hydrogen peroxide detoxification properties of DNA-binding protein from starved cells. a ferritin-like DNA-binding protein of *Escherichia coli* . J. Biol. Chem. 277 (31), 27689–27696. doi: 10.1074/jbc.M202094200 12016214

[B311] Zheng H. Chruszcz M. Lasota P. Lebioda L. Minor W. (2008). Data mining of metal ion environments present in protein structures. J. Inorganic Biochem. 102 (9), 1765–1776. doi: 10.1016/j.jinorgbio.2008.05.006 PMC2872550 18614239

[B312] Zhu W. Richards N. G. J. (2017). Biological functions controlled by manganese redox changes in mononuclear Mn-dependent enzymes. Essays Biochem. 61 (2), 259–270. doi: 10.1042/EBC20160070 28487402

[B313] Zygiel E. M. Nolan E. M. (2018). Transition metal sequestration by the host-defense protein calprotectin. Annu. Rev. Biochem. 87, 621–643. doi: 10.1146/annurev-biochem-062917-012312 29925260 PMC6066180

